# Transcription Factor EB Controls Metabolic Flexibility during Exercise

**DOI:** 10.1016/j.cmet.2016.11.003

**Published:** 2017-01-10

**Authors:** Gelsomina Mansueto, Andrea Armani, Carlo Viscomi, Luca D’Orsi, Rossella De Cegli, Elena V. Polishchuk, Costanza Lamperti, Ivano Di Meo, Vanina Romanello, Silvia Marchet, Pradip K. Saha, Haihong Zong, Bert Blaauw, Francesca Solagna, Caterina Tezze, Paolo Grumati, Paolo Bonaldo, Jeffrey E. Pessin, Massimo Zeviani, Marco Sandri, Andrea Ballabio

**Affiliations:** 1 Telethon Institute of Genetics and Medicine (TIGEM), Via Campi Flegrei 34, 80078 Pozzuoli, Naples, Italy; 2 Department of Biomedical Science, University of Padova, Padova 35121, Italy; 3 MRC Mitochondrial Biology Unit, Cambridge CB2 0XY, UK; 4 Fondazione IRCCS Istituto Neurologico “C. Besta,” 20133 Milan, Italy; 5 Venetian Institute of Molecular Medicine, Padova 35129, Italy; 6 Department of Molecular and Cellular Biology, Baylor College of Medicine, Houston, TX 77030, USA; 7 Hepatobiliary, Pancreatic, and Intestinal Research Institute, North Sichuan Medical College, Nanchong, Sichuan 637000, China; 8 Departments of Medicine and Molecular Pharmacology, Albert Einstein College of Medicine, Bronx, NY 10461, USA; 9 Department of Molecular Medicine, University of Padova, Padova 35121, Italy; 10 Medical Genetics, Department of Pediatrics, Federico II University, Via Pansini 5, 80131 Naples, Italy; 11 Department of Molecular and Human Genetics, Baylor College of Medicine, Houston, TX 77030, USA; 12 Jan and Dan Duncan Neurological Research Institute, Texas Children’s Hospital, Houston, TX 77030, USA

**Keywords:** TFEB, autophagy, exercise, glucose, mitochondria, diabetes, mitochondrial fusion, PGC1alpha, metabolic flexibility, insulin

## Abstract

The transcription factor EB (TFEB) is an essential component of lysosomal biogenesis and autophagy for the adaptive response to food deprivation. To address the physiological function of TFEB in skeletal muscle, we have used muscle-specific gain- and loss-of-function approaches. Here, we show that TFEB controls metabolic flexibility in muscle during exercise and that this action is independent of peroxisome proliferator-activated receptor-γ coactivator1α (PGC1α). Indeed, TFEB translocates into the myonuclei during physical activity and regulates glucose uptake and glycogen content by controlling expression of glucose transporters, glycolytic enzymes, and pathways related to glucose homeostasis. In addition, TFEB induces the expression of genes involved in mitochondrial biogenesis, fatty acid oxidation, and oxidative phosphorylation. This coordinated action optimizes mitochondrial substrate utilization, thus enhancing ATP production and exercise capacity. These findings identify TFEB as a critical mediator of the beneficial effects of exercise on metabolism.

## Highlights

• TFEB regulates mitochondrial biogenesis and function in muscle
• Glucose homeostasis in skeletal muscle requires TFEB
• The effects of TFEB on muscle metabolism are independent from PGC1α
• TFEB coordinates metabolic flexibility during physical exercise

## Graphical Abstract

**Figure:**
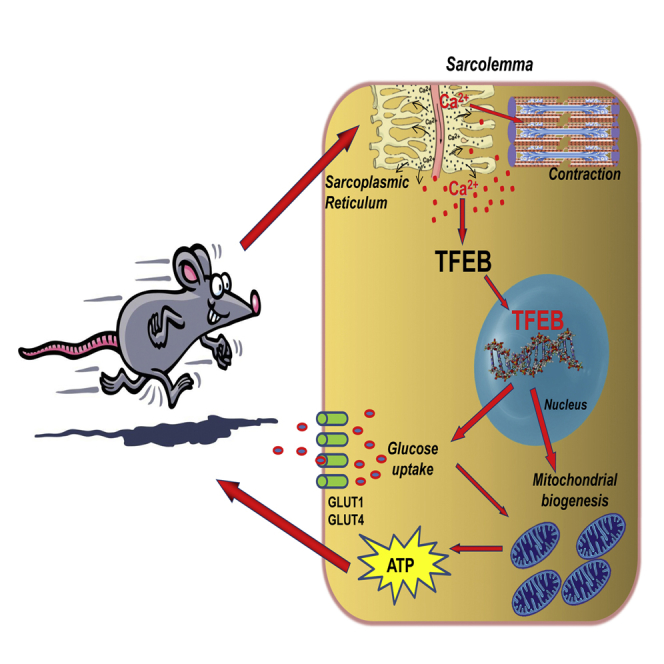

## Teaser

Mansueto et al. show that TFEB acts as a central coordinator of skeletal muscle insulin sensitivity, glucose homeostasis, lipid oxidation, and mitochondrial function in the adaptive metabolic response to physical exercise in a PGC1α-independent manner.

## Introduction

Exercise elicits several beneficial effects by acting on mitochondrial content/function, fatty acid oxidation, and glucose homeostasis (Hawley, 2002, Holloszy and Coyle, 1984, Holloszy et al., 1998). Indeed, muscle activity is important to counteract disease progression in diabetes, obesity, and metabolic syndrome. The signaling pathways that control the contraction-mediated beneficial effects on mitochondria and glucose/lipid homeostasis are distinct from insulin signaling and mainly rely on AMPK and PGC1α. We have recently found that exercise leads to nuclear translocation of the helix-loop-helix leucine zipper transcription factor EB (TFEB) (Medina et al., 2015), an important regulator of lysosomal biogenesis and autophagy (Sardiello et al., 2009, Settembre et al., 2011). Upregulation of TFEB has been found in several tissues after food deprivation, including liver and skeletal muscle. We have previously shown that in liver, TFEB regulates genes involved in lipid catabolism, fatty acid oxidation, and ketogenesis (Settembre et al., 2013). Some of these effects are elicited by TFEB-mediated induction of PGC1α (Settembre et al., 2013), a transcriptional coactivator, which interacts with and enhances the activity of transcription factors involved in mitochondrial biogenesis, glucose homeostasis, and lipid oxidation (Kelly and Scarpulla, 2004, Puigserver et al., 1998).

In the presence of nutrients, TFEB is sequestered in the cytoplasm by mTORC1-mediated phosphorylation, whereas in nutrient-depleted conditions, mTORC1 is inactive and dephosphorylated TFEB translocates to the nucleus, where it induces the transcription of target genes (Martina et al., 2012, Roczniak-Ferguson et al., 2012, Settembre et al., 2012). The dephosphorylation of TFEB is mediated by the calcium-dependent phosphatase, calcineurin, which is necessary for TFEB activation (Medina et al., 2015). Importantly, exercise-dependent calcium influx activates calcineurin, which dephosphorylates TFEB, leading to nuclear localization. The calcineurin-mediated induction of TFEB is independent from mTORC1 activity, indicating that calcium-dependent signaling is a rate-limiting step of TFEB activation (Medina et al., 2015).

Previous studies implicated calcineurin in a variety of physiological processes, in particular in skeletal muscle adaptation to exercise (Gehlert et al., 2015). Muscle-specific transgenic mice that overexpress an activated form of calcineurin show increased glucose uptake, glycogen accumulation, and lipid oxidation (Long et al., 2007). Interestingly, calcineurin promotes the nuclear translocation of another family of transcription factors, NFAT, which, depending on the type of physical activity, modulate the expression of the different myosin isoforms (Calabria et al., 2009, Long et al., 2007, McCullagh et al., 2004).

Here we show that the calcineurin-TFEB axis plays a major role in the metabolic adaptations that occur during physical exercise. By using gain- and loss-of-function approaches, we show that TFEB regulates mitochondrial biogenesis and glucose uptake independently of PGC1α. Indeed, TFEB controls genes involved in glucose metabolism such as GLUT1 and GLUT4, hexokinase I and II, TBC1 domain family member 1 (TBC1D1), and glycogen synthase (GYS), leading to glycogen accumulation to sustain energy production during exercise.

## Results

### Genome-wide Analyses Identified Glucose-Related and Mitochondrial Genes as Downstream Targets of TFEB

We previously demonstrated that TFEB promotes lipid catabolism in the liver and protects against diet-induced weight gain and insulin resistance (Settembre et al., 2013). Here we studied the physiological relevance of TFEB in skeletal muscle, an important insulin- and autophagy-dependent tissue (Grumati and Bonaldo, 2012, Mammucari et al., 2007, Masiero et al., 2009). Transcriptome analysis was performed by whole-genome gene expression profiling experiments (SuperSeries-GSE62980) in skeletal muscle from both *TFEB*-overexpressing and *TFEB* knockout (KO) mice. Overexpression of *Tcfeb*, the murine homolog of human *TFEB*, in muscle was achieved by means of intramuscular viral-mediated gene transfer using the adeno-associated virus (AAV) system. Adult mice were injected intramuscularly with either AAV2.1-CMV-*TFEB* or AAV2.1-CMV-*GFP* control vector and animals were sacrificed after 21 days, a time that allows efficient TFEB expression (Figure S1A, available online). Muscle-specific conditional *TFEB* KO mice were generated by crossing *Tcfeb* floxed (Settembre et al., 2013) with MLC1f-Cre transgenic mice (Bothe et al., 2000). Efficiency and specificity of the gene deletion were confirmed by quantitative real-time PCR analysis on multiple tissues (Figure S1B).

**Figure 1:**
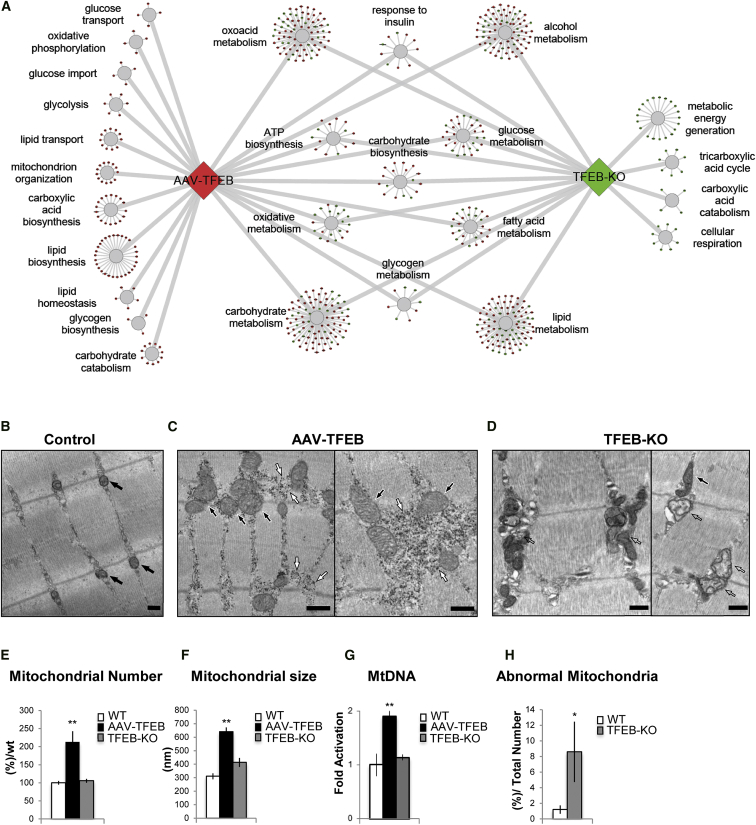
TFEB Induces Mitochondrial Biogenesis (A) Genes involved in lipid and glucose metabolism that were affected by *TFEB* expression are shown in colored circles. The upregulated (red circles) and downregulated genes (green circles) are shown in *TFEB*-overexpressing and *TFEB* KO, respectively. The genes were divided into functional sub-categories. (B–D) EM analysis of skeletal muscles from WT mice transfected with AAV2.1-*GFP* (B) and AAV2.1-*TFEB* (C), and from *TFEB* KO mice (D). Normal mitochondria are indicated by black arrows (B and C), while abnormal mitochondria in *TFEB* KO muscle (D) are indicated by empty arrows. Accumulation of glycogen is shown by white arrows in (C). The scale bars represent 500 nm. (E and F) Morphometrical analyses of mitochondrial number (E) and size (F) in *TFEB*-overexpressing and *TFEB* KO muscles compared to controls. Error bars represent mean ± SE, n = 3; ^∗∗^p < 0.01. (G) mtDNA analysis of muscles infected with AAV2.1-*TFEB* and from *TFEB* KO compared to WT mice. Quantitative real-time PCR of mtDNA copy numbers. Data are shown as mean ± SE, n = 3; ^∗∗^p < 0.01. (H) Quantification of abnormal mitochondria in *TFEB* KO gastrocnemius (GCN) muscles compared to WT muscles. Data are expressed as percentage of abnormal mitochondria on total mitochondria of 16 pictures (for detailed description of the criteria, see Experimental Procedures). Error bars represent mean ± SE; ^∗^p < 0.05.

Overexpression of *TFEB* in muscle resulted in the upregulation of 1,514 genes and the downregulation of 1,109 genes (GSE62975), while genetic ablation of *TFEB* increased 496 genes and suppressed 458 genes (GSE62976). The up- or downregulated genes are highlighted in red and green, respectively, in Tables S1 and S2. To identify the main cellular compartments (CCs) and the principal biological process (BPs) for which the TFEB-dependent genes were enriched, we performed a gene ontology enrichment analysis (GOEA). The GOEA was performed on the lists of genes whose expression was either increased or decreased in transfected muscle or in the *TFEB* KO mice. Interestingly, several gene categories related to cellular metabolism, including lipid and glucose homeostasis, were found upregulated in *TFEB*-overexpressing muscle and downregulated in *TFEB* KO (Figure 1A; Table S3). Strikingly, genes involved in mitochondrial biogenesis were oppositely regulated by gain- and loss-of-function approaches. Indeed, 38 genes involved in mitochondrial function were induced in AAV2.1-*TFEB*-infected muscles (Table S4), while 73 genes were inhibited in *TFEB* KO muscles (Table S5).

To better identify the network of genes regulated by TFEB in muscle, we performed sequence analysis to identify putative TFEB target sites, previously referred to as CLEAR sites (coordinated lysosomal expression and regulation) (Palmieri et al., 2011), in the promoter regions of the downregulated genes in *TFEB* KO mice. Interestingly, we found that 79% of these genes contain a CLEAR sequence and are, therefore, potential direct targets of TFEB (Table S6).

### TFEB Regulates Mitochondrial Biogenesis in Muscle

To examine potential effects of TFEB in mitochondrial function, we analyzed mitochondrial morphology in muscles overexpressing or lacking *TFEB*. Electron microscopy (EM) analyses showed a striking increase of mitochondrial density and volume in *TFEB*-overexpressing muscles (Figures 1B, 1C, 1E, and 1F). Interestingly, mitochondrial density and size were normal in the *TFEB* KO muscles (Figures 1E and 1F). Consistent with the EM data, increase of mitochondrial DNA (mtDNA) was found in TFEB transgenic muscles, while no differences were observed in *TFEB* KO muscles (Figure 1G). However, while the cristae shape, matrix density, and outer membrane morphology were normal in *TFEB*-overexpressing muscles (Figure 1C), abnormalities were found in approximately 10% of the mitochondria from *TFEB* KO muscles (Figures 1D and 1H). An increase in the number of mitochondria was also observed in C2C12 muscle cells transfected with *TFEB*-GFP, as detected by immunofluorescence confocal and confirmed by EM analyses (Figures S2A and S2B). Quantitative real-time PCR also revealed an increase of mtDNA content in *TFEB*-overexpressing cells (Figure S2C).

**Figure 2:**
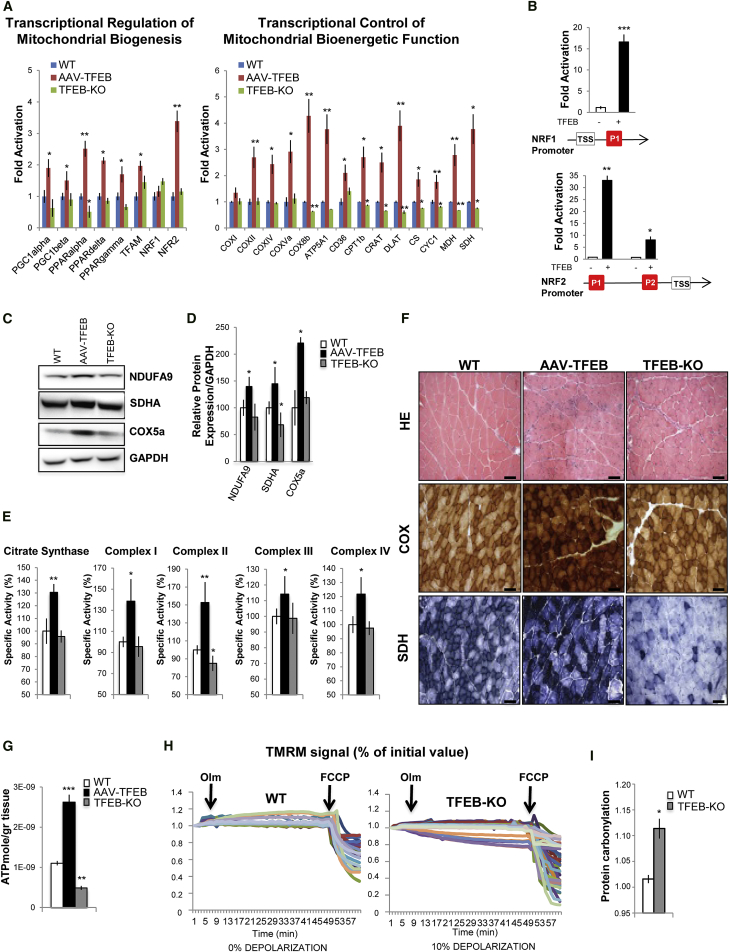
TFEB Regulates Mitochondrial Function (A) Quantitative real-time PCR of genes related to mitochondrial biogenesis and function in muscles of WT (blue bar), *TFEB*-overexpressing (red bar), and *TFEB* KO (green bar) mice. Data were normalized for GAPDH and expressed as fold induction relative to the WT. Data are expressed as mean ± SE, n = 3; ^∗^p < 0.05, ^∗∗^p < 0.01. (B) ChIP analysis in muscles from *TFEB*-overexpressing and WT mice. The CLEAR elements of NRF1 and NRF2 promoters are depicted as red boxes. TSS (transcriptional start site) indicates the first codon. The histograms show the amount of immunoprecipitated DNA as detected by quantitative real-time PCR assay. Data represent mean ± SE of three independent experiments; ^∗^p < 0.05, ^∗∗^p < 0.01, ^∗∗∗^p < 0.001. (C) Western blots of mitochondrial respiratory chain proteins. Blot is representative of four independent experiments. (D) Densitometric quantification of blots shown in (B). Data are shown as means ± SE (n = 4); ^∗^p < 0.05. (E) Mitochondrial respiratory chain activity in muscles of WT, AAV2.1-*TFEB* transfected, and *TFEB* KO mice. Data are expressed as a percentage relative to the WT. Data are expressed as mean ± SE, n = 4; ^∗^p < 0.05, ^∗∗^p < 0.01. (F) H&E, succinate dehydrogenase (*SDH*), and cytochrome oxidase (*COX*) staining of AAV2.1-GFP, AAV2.1-*TFEB* transfected, and muscles from *TFEB* KO mice. The scale bars represent 100 μm. Magnification, 20×. (G) ATP production rate per gram of tissue from *TFEB*-overexpressing and *TFEB* KO mice compared to controls. Data are shown as means ± SE (n = 5); ^∗∗^p < 0.01, ^∗∗∗^p < 0.001. (H) Mitochondrial membrane potential of myofibers isolated from FDB muscles of WT and *TFEB* KO mice. Where indicated, 6 μM oligomycin or 4 μM FCCP were added. Each trace represents the tetramethylrhodamine methyl ester (TMRM) fluorescence of a single fiber. The fraction of myofibers with depolarizing mitochondria is indicated for each condition. (I) Densitometric quantification of the carbonylated proteins extracted from WT and *TFEB* KO mice. Values are mean ± SEM (n = 4; ^∗^p < 0.05). A representative immunoblot for carbonylated proteins is shown in Figure S3C.

Importantly, quantitative real-time PCR analysis revealed that *TFEB* overexpression in muscle and in C2C12 cells induces the expression of many genes involved in mitochondrial biogenesis and function, including the master gene of mitochondrial biogenesis, PGC1α, a known direct target of TFEB (Settembre et al., 2013) (Figures 2A and S2D). Moreover, another PGC-1 family member, PGC1β, was also upregulated by *TFEB* overexpression. Consistently, we found a significant induction of peroxisome proliferator-activated receptor α (PPARα), PPARβ/δ, and PPARγ in *TFEB*-overexpressing muscles. However, *TFEB* deletion did not affect the expression of PGC1α/β and PPAR genes, with the exception of PPARα, which was downregulated. In order to elucidate the possible mechanisms underlying the induction of mitochondrial biogenesis observed in *TFEB*-transfected muscles, we examined the expression of nuclear respiratory factors 1 and 2 (NRF1and NRF2). The mRNA levels of NRF2 were increased, as well as NRF downstream genes, including mitochondrial transcription factor A (TFAM). Chromatin immunoprecipitation (ChIP) experiments confirmed the direct recruitment of TFEB on NRF1 and NRF2 promoters (Figure 2B), but not on TFAM promoter (data not shown). Finally, overexpression of *TFEB* in skeletal muscle increased the expression of mitochondrial enzymes. Subunits of the four respiratory chain complexes and the ATP synthase, as well as genes encoding electron transport and tricarboxylic acid cycle proteins, were induced by *TFEB* overexpression and were reduced by *TFEB* deletion (Figure 2A). Importantly, immunoblotting analyses confirmed the increase of complex I (NDUFA9), complex II (SDHA), and complex IV (COX5a) proteins in *TFEB*-transfected muscles (Figures 2C and 2D). *TFEB* deletion did not alter NDUFA9 and COX5a expression but significantly reduced the level of SDHA protein (Figures 2C and 2D). To better characterize the involvement of TFEB in mitochondrial respiration, we analyzed the specific activities of enzymes involved in oxidative phosphorylation. Biochemical analysis of muscle samples infected with AAV2.1-*TFEB* compared to wild-type (WT) muscles showed increase of citrate synthase, mitochondrial respiratory chain complex I (CI), CII, CIII, and CIV activities (Figure 2E). Consistent with the western blot analyses, *TFEB* deletion led to decrease of CII activity, the complex that contains the SDHA flavoprotein, while the other respiratory complexes had normal activities (Figure 2E). The changes in respiratory chain activities were corroborated by histochemical analyses for COX and SDH activity in AAV2.1-*TFEB*-transfected and *TFEB* KO muscles. Indeed, only SDH activity was greatly reduced in the absence of *TFEB*, while both COX and SDH were increased in *TFEB*-overexpressing muscles (Figure 2F). To further investigate the role of TFEB in mitochondrial function, we generated an inducible muscle-specific transgenic mouse line. Acute activation of TFEB by tamoxifen treatment in adult mice recapitulated the phenotype of AAV2.1-*TFEB* overexpression on mitochondria biogenesis (Figure S3). Consistent with the increase of SDH and respiratory chain complex activity, mitochondrial respiration was significantly enhanced by *TFEB* expression in adult transgenic muscles (Figures S3A and S3B).

We next assessed whether these TFEB-mediated changes of mitochondrial morphology and function had any impact on energy production. ATP levels were higher in *TFEB*-transfected muscles and lower in *TFEB*-deficient muscles compared to controls (Figure 2G). To understand the mechanisms underlying the significant decrease of ATP in *TFEB* KO muscles, we checked mitochondrial function in these animals. Fluorescent dyes, like tetramethylrhodamine methyl ester (TMRM), monitor the mitochondrial membrane potential (Δψm), the critical parameter that drives ATP production. Therefore, we checked the status of Δψm in isolated adult fibers of WT and *TFEB* KO muscles. As expected, in control mice oligomycin-dependent inhibition of ATP synthase did not alter Δψm (Figure 2H), and mitochondrial depolarization was achieved after membrane permeabilization by the protonophore carbonylcyanide-p-trifluoromethoxy phenylhydrazone (FCCP). Conversely, mitochondria of *TFEB* null fibers underwent a significant depolarization after oligomycin treatment (Figure 2G), suggesting that these fibers were at least in part relying on reverse activity of ATP synthase to preserve their membrane potential as a consequence of proton membrane leak. In addition, oxidative stress, revealed by protein carbonylation, was significantly higher in *TFEB* KO mice than controls (Figures 2I and S3C).

### TFEB Regulates Mitochondrial Biogenesis in Skeletal Muscle through a PGC1α- and PGC1β-Independent Mechanism

**Figure 3:**
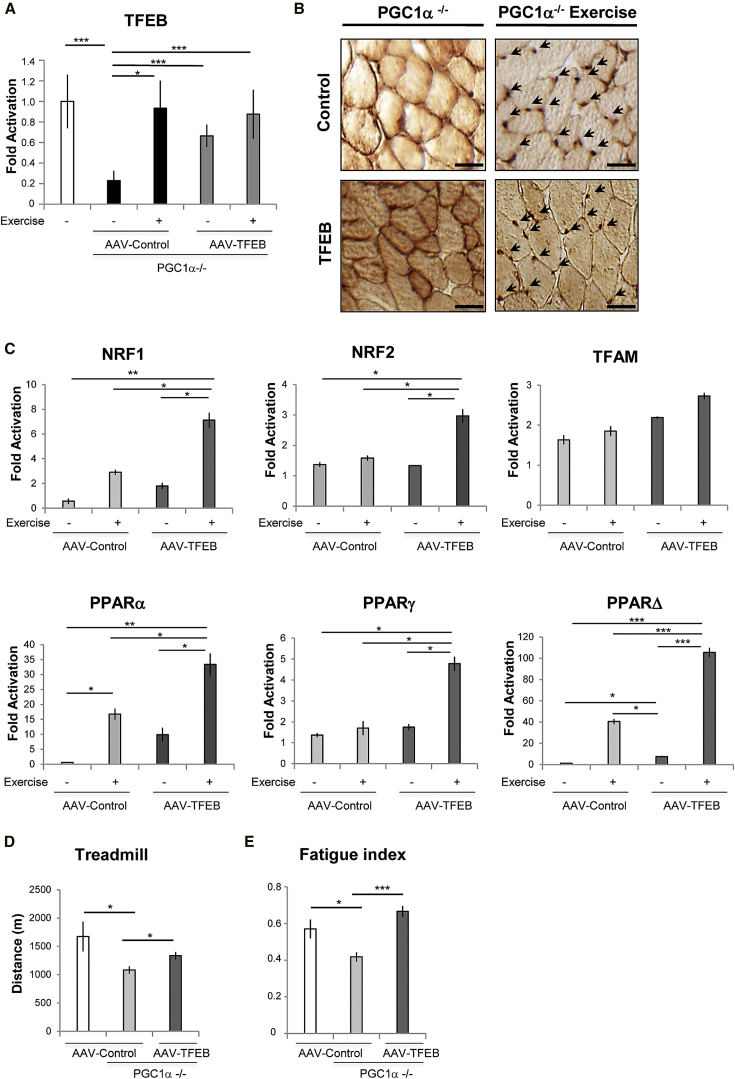
Exercise Induces TFEB Expression and Nuclear Localization in *PGC1α*^−/−^ Muscle (A) TFEB mRNA levels of *PGC1α*^−/−^ muscles infected with AAV2.9 control virus (light gray) or AAV2.9-*TFEB* (dark gray). Data were compared with TFEB mRNA level of WT mice (white). The levels of TFEB were measured in sedentary condition and post-exercise as indicated. Error bars represent mean ± SE for n = 3; ^∗^p < 0.05, ^∗∗∗^p < 0.001. (B) TFEB immunohistochemical analysis of *PGC1α*^−/−^ muscles from sedentary and exercised mice. GCN muscle cryosections were immunostained by using anti-TFEB antibody. Control means endogenous TFEB in muscles infected with AAV2.9 control virus. TFEB means the transgene TFEB in muscles infected with AAV2.9-*TFEB*. Arrows indicate exercise-induced TFEB nuclear localization. The scale bars represent 50 μm. (C) Analysis of genes related to mitochondrial biogenesis in *PGC1α*^−/−^ skeletal muscle infected with AAV2.9 control or with AAV2.9-*TFEB* virus. The mRNA levels were measured before and after acute exercise, as indicated. Data are shown as mean ± SE, n = 3; ^∗^p < 0.05, ^∗∗^p < 0.01, ^∗∗∗^p < 0.001. (D and E) Muscle fatigue and physical performance are improved by TFEB expression in PGC1α-deficient muscle. (D) The mice were systemically injected with AAV2.9-*TFEB* or AAV2.9 control virus. After 3 weeks from the virus injection, mice were subjected to run until exhaustion, as described in the Experimental Procedures. Data are shown as mean ± SE, n = 6; ^∗^p < 0.05. (E) Soleus muscles were transected by intramuscular injection of AAV2.1-*TFEB* or AAV2.1 control virus, which resulted in 100% transfection efficacy. Index for fatigue means tetanic muscle force generated after 100 s of stimulation divided by the maximal tetanic tension produced during the fatigue protocol. Data are shown as mean ± SE, n = 4; ^∗^p < 0.05, ^∗∗∗^p < 0.001.

Both PGC1α and PGC1β are master regulators of mitochondrial biogenesis and oxidative metabolism. However, recent findings suggest the presence of an independent pathway that regulates mitochondrial biogenesis during exercise (Rowe et al., 2012). Therefore, we examined whether TFEB is the missing sensor of physical activity that coordinates the metabolic responses independently of PGC1α. First, we checked expression and localization of endogenous TFEB in PGC1α KO mice before and after exercise. TFEB was expressed at lower levels and more cytosolic in *PGC1α* KO mice when compared to controls (Figures 3A and 3B). Importantly, exercise restored a normal TFEB expression, induced TFEB nuclear translocation, and triggered upregulation of genes related to mitochondrial biogenesis (Figures 3C and S4A).

EM analysis of overexpressed *TFEB* in *PGC1*α KO mice revealed increased mitochondrial volume and density (Figure S4B). *TFEB* overexpression in *PGC1*α KO muscle also resulted in increased COX and SDH activity along with increased activities of CI, II, III, and IV (Figures S4C and S4D). The levels of PGC1α targets such as TFAM, NRF1, and NRF2 were also significantly upregulated by *TFEB* overexpression, even in the absence of PGC1α (Figure S5A). Importantly, systemic delivery of TFEB in muscle-specific *PGC1α* KO mice improved their exercise tolerance (Figure 3D). Indeed, *TFEB* expression was able to restore normal fatigue index when expressed in *PGC1α* KO muscle (Figure 3E). Altogether, these findings suggest that the induction of mitochondrial biogenesis in *TFEB*-overexpressing muscles does not depend on the presence of PGC1α.

To determine whether PGC1β may compensate for the lack of PGC1α, we measured the effect of *TFEB* overexpression on mitochondrial biogenesis in cells that were silenced for PGC1α and PGC1β. Importantly, inhibition of both PGC1 factors did not prevent or reduce TFEB-mediated induction of genes related to mitochondrial biogenesis and mitochondrial respiratory chain activity (Figure S5B).

### TFEB Controls Energy Balance in Skeletal Muscle during Exercise

**Figure 4:**
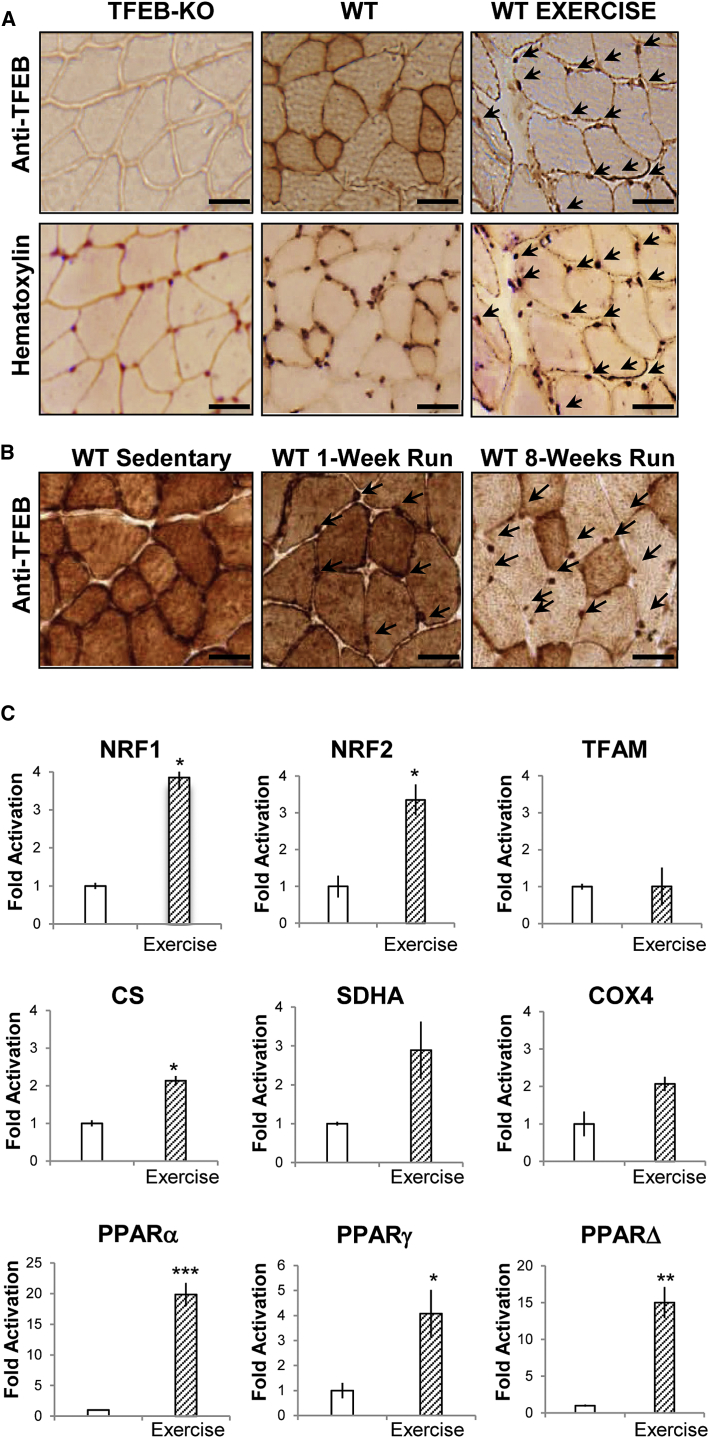
Exercise Induces TFEB Nuclear Translocation (A) GCN tissue sections of *TFEB* KO and WT mice were immunostained with anti-TFEB antibody and counter-stained with hematotoxylin as indicated. Arrows indicate nuclear localization of endogenous TFEB in WT mice after an acute bout of exercise. The scale bars represent 50 μm. (B) Endogenous TFEB was immunostained in muscle cryosections of sedentary, 4 days mild exercised, and 7 weeks intense trained WT mice. Arrows indicate TFEB nuclear localization. (C) Expression analysis of genes related to mitochondrial biogenesis and mitochondrial respiratory chain in WT skeletal muscle before and after acute exercise. Data are shown as mean ± SE, n = 3; ^∗^p < 0.05, ^∗∗^p < 0.01.

Physical activity has a major impact on glucose homeostasis and mitochondrial biogenesis and function. Therefore, we checked whether acute exhausting versus mild and chronic exercise regiments are equally able to activate TFEB. As a readout of TFEB activation, we monitored its nuclear localization. While acute exhausting contraction led to nuclear translocation of TFEB (Figure 4A), mild exercise did not (Figure 4B). However, 7 weeks of training with progressive increase of intensity without reaching exhaustion induced a massive TFEB nuclear translocation with concomitant cytosolic depletion (Figure 4B). Therefore, intensity and duration of training are critical factors that affect TFEB nuclear translocation and transcriptional regulation of genes related to mitochondrial biogenesis and function (Figure 4C).

**Figure 5:**
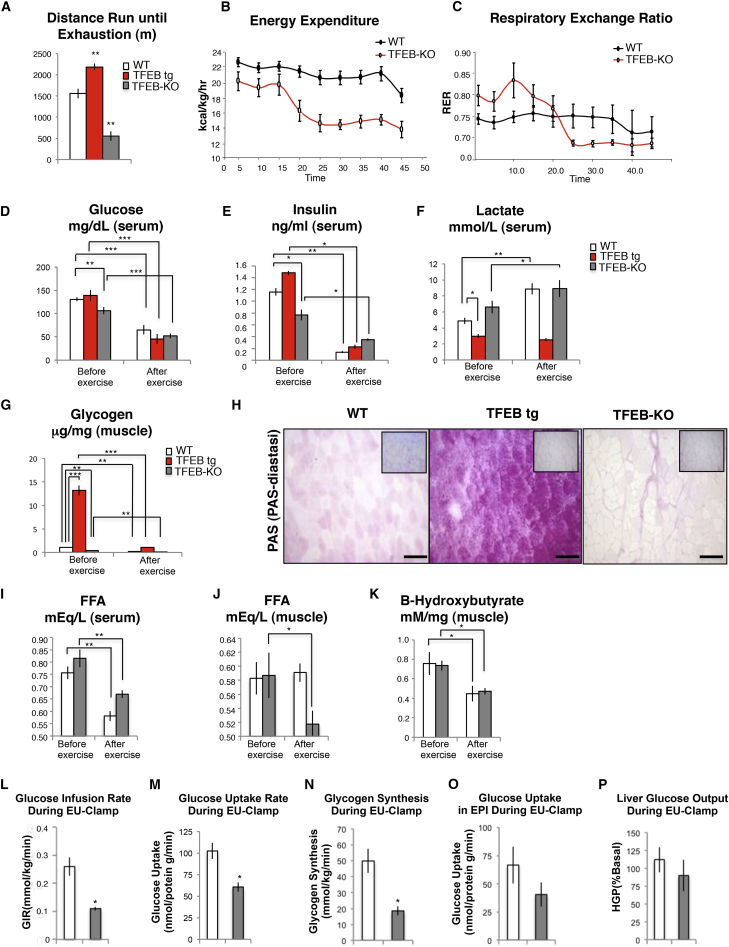
TFEB Controls Energy Balance in Skeletal Muscle (A) High-intensity exhaustive exercise. To determine exercise capacity, mice were run on a treadmill. During high-intensity exercise, transgenic mice (red) ran more, while *TFEB* KO mice (gray) ran half as much as WT mice (white). Data are shown as mean ± SE, n = 10; ^∗∗^p < 0.01. (B and C) Energy expenditure (B) and RER (C) were determined during exercise in *TFEB* KO and WT mice. The mice ran at a fixed speed of 10 m/min and an incline of 20°. The figure shows the mean RER measured at peak oxygen consumption. Data were transformed by Blom’s method to obtain both normally distributed data and normally distributed residual. Two-way ANOVA was used for comparison of RERs in the two groups during the entire resting period, whereas t test was used for comparison of individual time points, n = 8. (D–F) Blood and muscle metabolites before and after high-intensity exercise in WT (white), TFEB transgenic (red), and *TFEB* KO mice (gray). Data are shown as mean ± SE, n = 8; ^∗^p < 0.05, ^∗∗^p < 0.01, ^∗∗∗^p < 0.001. (G) Enzymatic quantification of muscle glycogen before and after high-intensity exercise in WT (white), TFEB transgenic (red), and *TFEB* KO mice (gray). Data are shown as mean ± SE, n = 8; ^∗∗^p < 0.01. (H) Periodic acid-Schiff (PAS) staining of cryosections from AAV2.1-*GFP*, AAV2.1-*TFEB* transfected, and *TFEB* KO mice. Inserts show PAS staining after glycogen breakdown. The scale bars represent 100 μm. (I and J) Quantitative analysis of serum (I) and muscle (J) NEFA before and after high-intensity exercise in *TFEB* KO (gray) and WT mice (white). Data are shown as mean ± SE, n = 8; ^∗^p < 0.05, ^∗∗^p < 0.01. (K) Enzymatic quantification of B-hydroxybutyrate before and after high-intensity exercise in *TFEB* KO (gray box) and WT mice (white box). Data are shown as mean ± SE, n = 8; ^∗^p < 0.05. (L) TFEB ablation results in a decreased rate of insulin-stimulated GIR. EU clamps were used to assess whole-body insulin sensitivity by determining the GIR required to maintain euglycemia in WT (white) and *TFEB* KO (gray) mice. Data represent the means ± SE from five to six individual mice per group; ^∗^p < 0.05. (M) TFEB deletion results in a reduction of insulin-stimulated glucose uptake in muscle. Insulin-stimulated glucose uptake into muscle tissues was determined by 2-deoxy-d-[1-^14^C]glucose injection during the last 35 min of insulin infusion during the EU clamp. These data represent the means ± SE of five to six mice per group; ^∗^p < 0.05. WT, white; *TFEB* KO, gray. (N) The amount of glucose conversion to glycogen in GCN muscles from WT (white) and *TFEB* KO (gray) mice was determined during the EU clamp by infusion of [3-^3^H]glucose. Data represent the means ± SE from five to six mice per group; ^∗^p < 0.05. (O and P) *TFEB* KO mice do not show any significant difference in glucose homeostasis of liver or adipose tissue. (O) Glucose uptake into adipose tissue (EPI) and (P) insulin suppression of hepatic glucose output (HGP, liver) were determined during the EU clamp by infusion of 2-deoxy-d-[1-^14^C]glucose or [3-^3^H]glucose infusion into WT mice (white) and *TFEB* KO (gray) mice. These data represent the means ± SE from five to six mice per group.

To determine the physiological consequences of TFEB translocation during physical activity, we examined exercise performance in both *TFEB* KO and inducible muscle-specific TFEB transgenic mice. High-intensity exercise revealed significant training intolerance of *TFEB* KO mice compared to controls (Figure 5A). Conversely, acute muscle-specific activation of TFEB enhanced physical performance (Figure 5A). To better understand the exercise intolerance of the *TFEB* KO mice, we examined energy expenditure during treadmill running. While WT mice maintained constant levels of energy expenditure during physical activity, the *TFEB* KO mice displayed a drop after 15 min of physical exercise (Figure 5B). Metabolic analyses revealed that in basal condition, *TFEB* KO mice have a higher respiratory exchange rate (RER) than controls (Figure 5C). These data suggest that *TFEB* KO mice depend on glucose oxidation more than controls. In addition, while WT mice maintained a relatively constant RER during running period, *TFEB* KO mice showed a drop in RER after 20 min (Figure 5C). This decrease indicates a shift in substrate usage from glucose to fat metabolism. Finally, we measured glucose and fatty acid levels in muscle and blood from *TFEB* KO and TFEB transgenic mice before and after exercise. *TFEB* KO mice showed lower blood glucose levels compared to WT mice in basal condition (Figure 5D). Exercise caused a 50% reduction of blood glucose in *TFEB* KO, TFEB transgenic, and control mice (Figure 5D). Insulin levels mirrored the changes of blood glucose, as they were reduced in basal condition in *TFEB* KO mice and dropped after exercise in the different genotypes (Figure 5E).

*TFEB* KO mice are hypoglycemic, contain dysfunctional mitochondria, and produce less ATP. Thus, we reasoned that they use anaerobic glycolysis to produce energy. Consistent with this hypothesis, we found higher levels of lactate in the blood of *TFEB* KO mice before and after exercise compared to controls (Figure 5F). Conversely, lactate of transgenic mice was already lower than controls in resting condition and did not increase after exercise (Figure 5F). Therefore, TFEB transgenic mice better utilize glucose for energy production. To further confirm this finding, we measured glycogen levels in muscle. Glycogen levels were remarkably lower in *TFEB* KO mice and higher in TFEB transgenic in basal condition compared to WT. Enzymatic quantification showed that glycogen content was 3-fold less after TFEB ablation (Figure 5G), while it was 10-fold higher after TFEB overexpression compared to controls (Figure 5G). This was confirmed by periodic acid-Schiff (PAS) staining (Figure 5H). Exercise led to glycogen consumption in both *TFEB* KO muscles and controls (Figure 5G). The lower glycogen content detected in sedentary *TFEB* KO muscles explains the decrease of RER observed after a 15 min exercise (Figure 5C). Since glycogen is rapidly depleted in *TFEB* KO mice, the additional need for energy during exhausting exercise requires a switch from glycolysis to fatty acid oxidation. Consistently, while blood free fatty acid concentrations were reduced after exercise in both *TFEB* KO mice and controls (Figure 5I) and blood levels of non-esterified fatty acids (NEFAs) did not differ between genotypes (Figure 5I), their muscle content dramatically decreased after exercise only in *TFEB* KO mice (Figure 5J). Importantly, ketones did not differ between *TFEB* KO and controls (Figure 5K). These findings confirm a change in metabolic flexibility in the absence of TFEB that forced muscle cells to use lipids for ATP production. The exhaustion of the lipid fuel in KO mice results in inability to maintain the same exercise intensity of controls.

### TFEB Controls Metabolic Flexibility and Energy Balance Independently of Autophagy

We and others have found that autophagy is important for mitochondrial quality control and is activated by exercise to clear dysfunctional mitochondria (Lo Verso et al., 2014). Thus, we checked whether TFEB controls autophagy in adult skeletal muscles. Surprisingly, TFEB activation was not sufficient to enhance autophagy flux and TFEB deletion did not impair autophagy flux in the presence or absence of nutrients (Figures S6A and S6B). Moreover, activation of TFEB did not induce protein breakdown and muscle loss. In fact, most of the atrophy-related genes belonging to the ubiquitin proteasome and autophagy-lysosome systems were not induced by TFEB expression (Figure S6C). Similarly, mitophagy genes were not upregulated. Since protein degradation is not affected by TFEB activation, we checked whether genes related to protein synthesis were modulated by TFEB. However, when we checked a cross-sectional area, we found a shift toward smaller size in transgenic mice, suggesting that protein synthesis was not induced. This decrease in fiber size is due to a metabolic shift because oxidative fibers are smaller than glycolytic skeletal muscle fibers (Figure S7A). Furthermore, we did not find any significant difference in myosin distribution between transgenic and control muscles (Figure S7B). Therefore, TFEB controls myofiber metabolism, but not myosin content/type, independently of autophagy or proteostasis.

### TFEB Controls Glucose Homeostasis and Insulin Sensitivity Independently of PGC1α

Because muscles from *TFEB* KO mice contain lower glycogen levels than controls, we reasoned that they may have abnormal regulation of glucose homeostasis. Thus, we performed euglycemic-hyperinsulinemic (EU) clamps and observed that the glucose infusion rate (GIR) that is required to maintain a constant glycemia during insulin treatment was significantly reduced in *TFEB* KO compared to control mice (Figure 5L). The reduction of GIR was consequent to a decrease in skeletal muscle glucose uptake (Figure 5M), which caused a decrease of glycogen synthesis (Figure 5N). Although there was an apparent small decrease in adipose tissue glucose uptake, this was not statistically significant (Figure 5O). Moreover, insulin was equally effective in suppressing hepatic glucose output during the clamp experiment (Figure 5P). Together, these data demonstrate that TFEB deficiency in skeletal muscle results in peripheral insulin resistance-reduced glucose uptake and decreased glycogen content.

**Figure 6:**
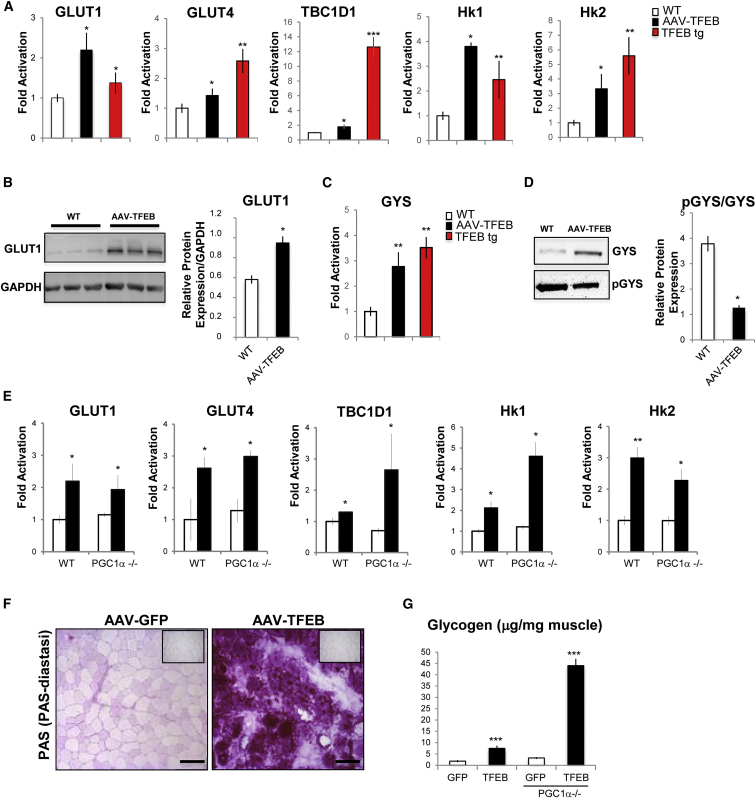
TFEB Controls Genes Related to Glucose Metabolism (A and C) Quantitative real-time PCR of glucose uptake and glycogen biosynthesis-related genes in GCN muscles infected with AAV2.1-TFEB (black bar) and in TFEB transgenic muscles (red bar) compared with WT (white bar) muscles. Data were normalized for GAPDH and expressed as fold induction relative to the WT. Data are shown as mean ± SE, n = 3; ^∗^p < 0.05, ^∗∗^p < 0.01. (B) Western blot of GLUT1 and densitometric quantification. Values are normalized for GAPDH; ^∗^p < 0.05. (D) Western blot analysis of total and phosphorylated GYS from extracts of GCN. Representative blot images are shown (left panel). Densitometric quantification is depicted on the right panel. Data are shown as mean ± SE, n = 3; ^∗^p < 0.05. (E–G) TFEB regulates glycogen synthesis independently of PGC1α. (E) Quantitative real-time PCR of genes related to glucose uptake and glycogen biosynthesis in *PGC1α*^−/−^ and WT mice that were infected with AAV2.1-*GFP* (white) or AAV2.1-*TFEB* (black). Data are shown as mean ± SE, n = 3; ^∗^p < 0.05, ^∗∗^p < 0.01. (F) PAS staining of cryosections from *PGC1α*^−/−^ muscles that were transfected by AAV2.1-*GFP* or AAV2.1-*TFEB*. The scale bars represent 100 μm. (G) Enzymatic quantification of basal glycogen levels in *PGC1α*^−/−^ and WT muscle, infected with AAV2.1-*GFP* (white) or AAV2.1-*TFEB* (black).

These findings are consistent with the transcriptomic signature of *TFEB*-overexpressing and *TFEB* KO muscles. To further confirm these findings, we monitored the expression levels of glucose homeostasis-related genes in muscles injected with AAV-*TFEB* and in muscles of TFEB transgenic mice and found a significant increase in the expression of GLUT1 and GLUT4, the GTPase involved in GLUT4 translocation (TBC1D1), and the rate-limiting enzymes of glycolysis (hexokinase 1 and 2) (Figure 6A). Immunoblotting analyses confirmed increased protein levels of GLUT1 in *TFEB*-overexpressing muscles (Figure 6B). The induction of genes involved in glucose uptake was also coupled with an increase of transcript and protein of GYS (Figures 6C and 6D).

GYS activity is negatively regulated by phosphorylation of the C-terminal region by GYS kinases (GSK3s) (Jensen and Lai, 2009). However, the phospho-GYS levels were unchanged in AAV-*TFEB* muscles, and therefore, the pGYS/GYS ratio was dramatically decreased in *TFEB*-overexpressing muscles when compared to controls (Figure 6D). Furthermore, expression analysis of genes related to glucose metabolism did not reveal any significant difference between *PGC1α* KO and controls after *TFEB* overexpression (Figure 6E). Finally, EM showed an accumulation of glycogen in *PGC1α* KO mice that were infected by AAV2.1-*TFEB* (Figure S4B). Quantitative and qualitative analyses of glycogen showed that TFEB induced a higher glycogen accumulation in *PGC1α* KO muscle than in WT (Figures 6F and 6G). These data indicate that TFEB is able to control muscle glycogen content in a PGC1α-independent manner.

### Glucose-Related Signaling Pathways Are Affected by TFEB

**Figure 7:**
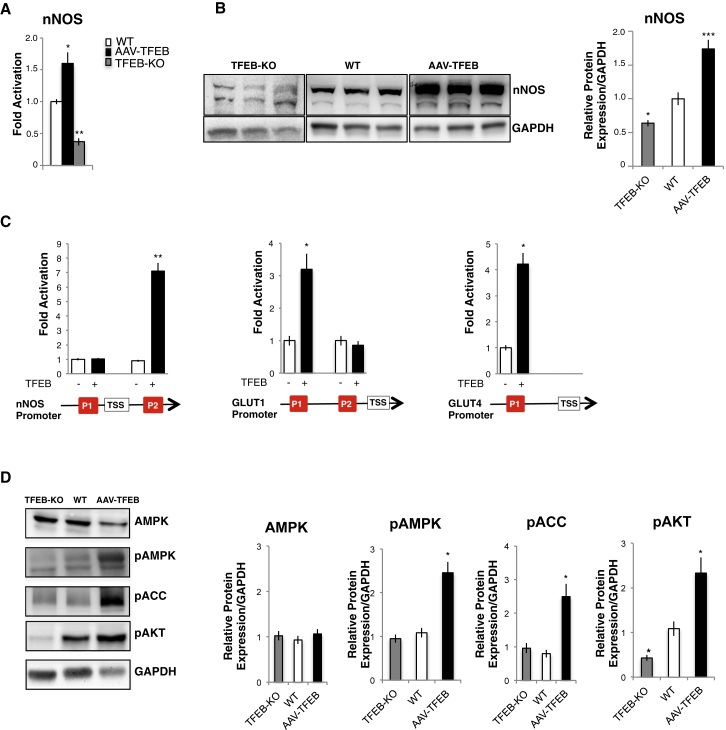
TFEB Also Controls Glucose Uptake via Regulation of AMPK Signaling Pathways (A) Quantitative real-time PCR of nNOS transcript in WT (white bar), *TFEB*-overexpressing (black), and *TFEB* KO muscles (gray). Values are expressed as fold induction compared to controls. Data are shown as mean ± SE, n = 8; ^∗^p < 0.05, ^∗∗^p < 0.01. (B) Western blot of nNOS and densitometric quantification. Value are normalized for GAPDH; ^∗^p < 0.05; ^∗∗∗^p < 0.001. (C) ChIP analyses of muscles from TFEB transgenic and WT mice on nNOS, GLUT1, and GLUT4 promoters. The histograms show the amount of immunoprecipitated DNA as detected by quantitative real-time PCR assay. Data represent mean ± SE of three independent experiments; ^∗^p < 0.05. (D) Western blot of AMPK, PAMPK, PACC, and PAKT performed on protein extracts from GCN muscles of AAV2.1-*TFEB* transfected, *TFEB* KO, or WT mice. Representative images (left) and densitometric quantification (right) are shown; ^∗^p < 0.05.

The activity of GLUT transporters is tightly controlled by several pathways. Thus, we checked whether TFEB impinges not only on GLUT1/4 expression but also on glucose-related signaling. Previous studies have shown that nitric oxide (NO) controls several metabolic aspects of skeletal muscle, including mitochondrial biogenesis and glucose uptake. NO is formed by nitric oxide synthase (NOS) via the conversion of L-arginine to L-citrulline. Skeletal muscles express neuronal (nNOS), endothelial (eNOS), and inducibile (iNOS) isoforms (Andrew and Mayer, 1999). Moreover, nNOS is the major isoform involved in AMPK-dependent regulation of GLUT4 (Lira et al., 2007). We found that the nNOS transcription and protein expression were increased in *TFEB*-overexpressing muscle. Conversely, real-time PCR and immunoblotting experiments showed a decrease of nNOS expression in *TFEB* KO muscle, as compared with controls (Figures 7A and 7B). To test whether nNOS and glucose transporter genes are direct targets of TFEB, we analyzed their promoters and identified CLEAR sites. ChIP experiments showed that TFEB is recruited on nNOS, GLUT1, and GLUT4 promoters (Figure 7C). Since AMPK is a downstream target of nNOS (Lira et al., 2010), we monitored the activation of AMPK. *TFEB* overexpression in muscle showed a significant induction of AMPK phosphorylation and of its downstream target acetyl-CoA carboxylase (ACC). Interestingly, *TFEB* overexpression triggered protein kinase B (AKT) activation. However, no changes in pAMPK and pACC were found in *TFEB* KO muscles (Figure 7D). Consistent with the presence of insulin resistance, we detected decreased AKT phosphorylation in *TFEB* KO muscles.

## Discussion

The beneficial effects of physical activity on mitochondrial content/function, fatty acid oxidation, and glucose homeostasis are well known (Hawley, 2002, Holloszy and Coyle, 1984, Holloszy et al., 1998). Indeed, muscle activity is important to counteract disease progression in diabetes, obesity, and metabolic syndrome. Here we have found that TFEB is a major regulator of glucose homeostasis and mitochondrial biogenesis to provide the energetic support to maintain muscle contraction. Our in vivo data show that the absence of TFEB causes accumulation of morphologically abnormal and dysfunctional mitochondria that display impairment in respiratory chain complex II activity and proton leakage, leading to a defect in ATP production and exercise intolerance. Conversely, overexpression of TFEB induces mitochondrial biogenesis, improves respiratory chain complex activities, and increases ATP production. These findings are surprising since the documented effects of TFEB are mainly related to lysosomal biogenesis and autophagy regulation.

The positive effects of TFEB on the mitochondrial network in skeletal muscle appear to be independent from PGC1α. We found that TFEB is sufficient to induce the expression of NRF2 and Tfam, two major master regulators of mitochondrial biogenesis in muscle, even in the absence of both PGC1α and PGC1β, and is both sufficient and required for PPARα expression. Therefore, TFEB acts independently of PGC1α to promote oxidation of glucose and lipids, which are critical substrates during the early and late phase of strenuous contraction, respectively. Therefore, TFEB is at least one missing factor that explains why PGC1α is dispensable for exercise and mitochondrial biogenesis (Rowe et al., 2012). Moreover, TFEB directly controls glucose homeostasis via GLUT1/4 expression and insulin sensitivity via nNOS. Indeed, muscle from *TFEB* KO mice showed decreased glucose uptake during EU clamps, nearly completely absent glycogen stores, and reduced AKT phosphorylation under resting conditions. The insulin resistance of the TFEB-deficient fibers prevents glucose oxidation and therefore drives the exercising muscle to use fatty acid oxidation, which consequently blocked pyruvate dehydrogenase (PDH) enzyme (Figure 2A), resulting in lactate accumulation. Altogether, these findings suggest that TFEB is a critical player of metabolic flexibility during physical activity.

In a previous study, we reported that TFEB regulates lipid metabolism in liver, and this effect appears to be mediated, at least in part, by PGC1α (Settembre et al., 2013). Conversely, our findings in muscle show that TFEB is directly involved in mitochondrial function and glucose homeostasis independently from PGC1α. These observations indicate that the networks of genes regulated by TFEB are context specific, for the gene expression profiles have significant tissue-specific changes, supporting distinct tissue-specific metabolic functions.

Calcium signaling is greatly affected by exercise, and the calcium-dependent phosphatase calcineurin is one of the most important players for muscle adaptation to physical activity. We have recently found that exercise triggers TFEB nuclear localization in a calcineurin-dependent fashion (Medina et al., 2015). Calcineurin dephosphorylates TFEB serine residues that play a critical role in determining TFEB subcellular localization and promotes its nuclear translocation (Medina et al., 2015). Importantly, calcium-dependent signaling also modulates exercise-dependent, glucose-related pathways. In fact, muscle-specific transgenic mice that overexpress an activated form of calcineurin show increased glycogen accumulation and lipid oxidation (Long et al., 2007) and upregulation of PGC1α, several glycolytic enzymes, mitochondrial genes, genes related to lipid metabolism, and GLUT4 (Long and Zierath, 2008). Conversely, muscle-specific calcineurin KO mice show exercise intolerance when subjected to exhausting physical activity (Pfluger et al., 2015). Finally, immunosuppression therapy by high-dose treatment with calcineurin inhibitors cylosporin A or tacrolimus (FK-506) has been associated with a higher risk for developing obesity and diabetes in patients (T D Correia et al., 2003). Altogether, these data suggest that calcineurin plays a major role in glucose and lipid metabolism, although the mechanistic insights of these beneficial effects of calcineurin are unknown. Our data suggest that TFEB is a critical calcineurin downstream target that coordinates metabolic adaptations such as glucose uptake and mitochondria function to optimize energy production to sustain muscle contraction.

TFEB not only regulates expression of glucose transporters and critical glycolytic enzymes but also factors that impinge on AMPK regulation, such as nNOS. It was shown that nNOS controls GLUT4 expression in skeletal muscle cells through AMPK activation. In addition, endogenous nNOS is required for the upregulation of AMPK activity by the AMP mimetic, AICAR (Higaki et al., 2001, Lira et al., 2010, McConell et al., 2010).

In summary, our results position TFEB as a central coordinator of insulin sensitivity, glucose homeostasis, lipid oxidation, and mitochondrial function, further emphasizing the importance of this regulatory pathway in the metabolic response to energy-demanding conditions such as physical exercise.

## Experimental Procedures

### Cell Culture, Plasmids, and Transfection Reagent

Detailed information in the Supplemental Experimental Procedures.

### Real-Time PCR

Quantitative real-time PCR analyses were performed on a LigthCycler 480 II (Roche). For detailed information on preparation and gene expression analysis, see the Supplemental Experimental Procedures.

### Generation of Muscle-Specific TFEB KO and Inducible Muscle-Specific Transgenic Mice and In Vivo Experimental Procedures

Detailed information in the Supplemental Experimental Procedures.

### Western Blot Analysis and Antibodies

Total homogenates were prepared in RIPA buffer (50 mM Tris HCl [pH 8], 150 mM NaCl, 1% NP-40, 0.5% sodium deoxycholate, and 0.1% SDS) with the addition of a protease inhibitor cocktail (Roche). Protein concentration was determined by the Lowry method. Aliquots, 50 μg each, were run through an SDS-PAGE and electroblotted onto a PVDF membrane, which was then matched with different antibodies. For the antibodies used in western blot analysis, see the Supplemental Experimental Procedures.

### Biochemical Analysis of Mitochondrial Respiratory Chain Complex

Muscle samples stored in liquid nitrogen were homogenized in 10 mM phosphate buffer (pH 7.4), and the spectrophotometric activity of cI, cII, cIII, and cIV, as well as citrate synthase (CS), was measured as described (Bugiani et al., 2004). Detailed information in the Supplemental Experimental Procedures.

### Morphological Analysis

Histochemical and ultrastructural analyses were performed as described (Sciacco and Bonilla, 1996). Detailed information in the Supplemental Experimental Procedures.

### Isolation of Skeletal Myofibers and Measurements of Mitochondrial Membrane Potential

Muscle fibers were isolated from FDB muscle and mitochondrial membrane potential measured by epifluorescence microscopy on the basis of the accumulation of TMRM fluorescence, as previously described (Lo Verso et al., 2014). Fibers were considered as depolarizing when they lost more than 10% of the initial value of TMRM fluorescence. Imaging was performed with a Zeiss Axiovert 100 TV inverted microscope equipped with a 12-bit digital cooled charge-coupled device camera (Micromax, Princeton Instruments). The results were analyzed with MetaFluor imaging software (Universal Imaging).

### Acute Exercise, Training, and Fatigue Experiments

For acute and chronic exercise studies, 16-week-old mice performed concentric exercise on a treadmill (Biological Instruments, LE 8710 Panlab Technology 2B) with a 10° incline, according to the protocol of exercise previously described (Medina et al., 2015). Total running distance was recorded for each mouse. Detailed information in the Supplemental Experimental Procedures.

### Immunohistochemistry

Detailed information in the Supplemental Experimental Procedures.

### Electron Microscopy

Detailed information in the Supplemental Experimental Procedures.

### Plasma Chemistry Analysis

Blood was collected from the orbital plexus under isoflurane (Vedco) anesthesia. Plasma was frozen in aliquots at −20°C or used immediately after collection. Specific enzymatic kits were used for determination of serum NEFAs (Wako) and lactate (Abcam). Plasma glucose was monitored by a glucometer. Insulin was measured by ELISA (Mercodia).

### Tissue Metabolite Quantification

Detailed information in the Supplemental Experimental Procedures.

### Whole-Body Indirect Calorimetry

Metabolic measurements were performed using an Oxymax indirect calorimetry system (Columbus Instruments) (Zong et al., 2011). Detailed information in the Supplemental Experimental Procedures.

### In Vivo Assessment of Insulin Action and Glucose Metabolism

Four days before the experiment, the mice were anesthetized and an indwelling catheter was introduced into the left internal jugular vein. The mice were fully recovered from the surgery before the in vivo experiments, as reflected by their reaching preoperative weight. After an overnight fast, EU clamps were conducted in conscious mice as previously described (Ayala et al., 2006, Zong et al., 2012). Detailed information in the Supplemental Experimental Procedures.

### ChIP Assays

We performed ChIP assays on adult skeletal muscle overexpressing *TFEB*3XFlag, and on WT muscle as control, by using the ChIP assay kit (Upstate) according to Milan et al. (2015). For immunoprecipitation, we used anti-FLAG antibody (F7425, Sigma-Aldrich). Oligonucleotide primers for amplification of a TFEB binding site on the GLUT1, GLUT4, nNOS, NRF1, NRF2, and TFAM promoters are listed in Table S7.

### Microarray Data Analysis

Detailed information in the Supplemental Experimental Procedures.

### Statistical Analysis

Data are expressed as mean values ± SE. Results were evaluated by repeated-measures ANOVA, multivariate ANOVA (MANOVA), or Student’s two-tailed t test. p < 0.05 was considered statistically significant. In all figures, ^∗^p < 0.05, ^∗∗^p < 0.01, and ^∗∗∗^p < 0.001.

## Author Contributions

G.M., M.S., and A.B. designed the project and experiments and wrote the manuscript. G.M., A.A., and C.V. performed experiments and analyzed and interpreted data. G.M. and A.A. generated transgenic mice. E.V.P., C.L., I.D.M., V.R., H.Z., B.B., F.S., and C.T. contributed to experiments and data collection; P.G. and P.B. provided reagents and discussed data; R.D.C. performed computational analysis; and L.D., S.M., and P.K.S. provided technical expertise. J.E.P., M.Z., M.S., and A.B. discussed, reviewed, and edited the manuscript. M.S. and A.B. supervised the work.

## Supplemental Information

Document S1. Supplemental Experimental Procedures, Figures S1–S7, and Tables S4 and S5

Table S1. TFEB Overexpression Muscle Dataset Related to Figure 1. Complete list of differentially expressed genes in TFEB overexpression muscle microarray dataset (GSE62975). The genes significantly (FDR<0.05; FoldChange ≥ 1.5) upregulated and downregulated are highlighted in red and in green, respectively.

Table S2. TFEB KO Muscle Dataset Related to Figure 1. Complete list of differentially expressed genes in TFEB-KO muscle microarray dataset (GSE62976). The genes significantly (FDR<0.05) upregulated and downregulated are highlighted in red and in green, respectively.

Table S3. TFEB Controls Lipid and Glucose Metabolism in Muscle Related to Figure 1. List of Lipid and Glucose Metabolism related terms related to the GOEA induced by TFEB overexpression and inhibited by TFEB-KO. For each term the genes significantly induced by TFEB overexpression (Supplemental Table BPALL_UP_OverTFEB) and inhibited by TFEB-KO (Supplemental Table BPALL_DOWN_KO-TFEB) are reported in table.

Table S6. CLEAR Sites on Mitochondrial Genes Related to Figure 2. Position and sequence of the CLEAR sites (i.e., the consensus TFEB binding-site) in the promoter region (1Kb upstream the TSS in the human and Mouse promoters) of mitochondrial genes downregulated in TFEB-KO muscle.

Table S7. CLEAR Sites in Promoter Region of Genes Related to Mitochondria Biogenesis and Glucose Metabolism Related to Figures 2 and 7. Position and sequence of TFEB binding site on the GLUT1, GLUT4, nNOS, TFAM, NRF1 and NRF2 promoter.

Document S2. Article plus Supplemental Information
