# Supplementary material for: Transcription Factor EB Controls Metabolic Flexibility during Exercise
Source: Cell Metab. 2017 Jan 10;25(1):182–96. doi: 10.1016/j.cmet.2016.11.003 (PMC5241227; doi:10.1016/j.cmet.2016.11.003)
Supplement: Document S1. Supplemental Experimental Procedures, Figures S1–S7, and Tables S4 and S5 [file mmc1.pdf]

Cell Metabolism, Volume *25*

Supplemental Information

Transcription Factor EB Controls Metabolic Flexibility during Exercise

Gelsomina Mansueto, Andrea Armani, Carlo Viscomi, Luca D'Orsi, Rossella De Cegli, Elena V. Polishchuk, Costanza Lamperti, Ivano Di Meo, Vanina Romanello, Silvia Marchet, Pradip K. Saha, Haihong Zong, Bert Blaauw, Francesca Solagna, Caterina Tezze, Paolo Grumati, Paolo Bonaldo, Jeffrey E. Pessin, Massimo Zeviani, Marco Sandri, and Andrea Ballabio

Figure S1.

A

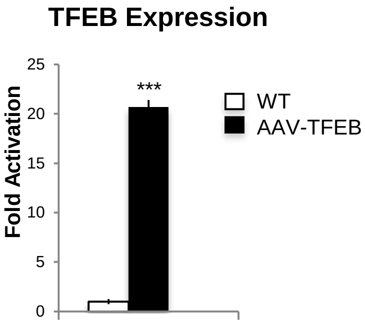
TFEB Expression
Fold Activation
25
20
15
10
5
0
***
WT
AAV-TFEB

B

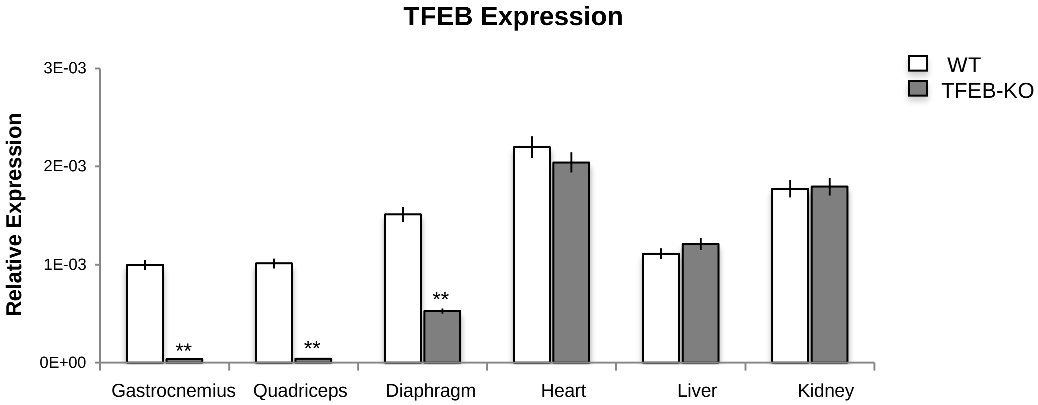
TFEB Expression
Relative Expression
3E-03
2E-03
1E-03
0E+00
**
**
**
WT
TFEB-KO
Gastrocnemius
Quadriceps
Diaphragm
Heart
Liver
Kidney

Figure S1. Expression analysis of TFEB transcript. Related to Figure 1. (A) The TFEB transcript from AAV2.1-*TFEB* transfected muscle was quantified by qRT-PCR (B) Expression levels of endogenous *Tcfeb* mRNA in muscles isolated from *TFEB*-KO and WT mice. Bar represent mean ± SE for $n = 5$; **p<0.01,***p<0.001s.

Figure S2.

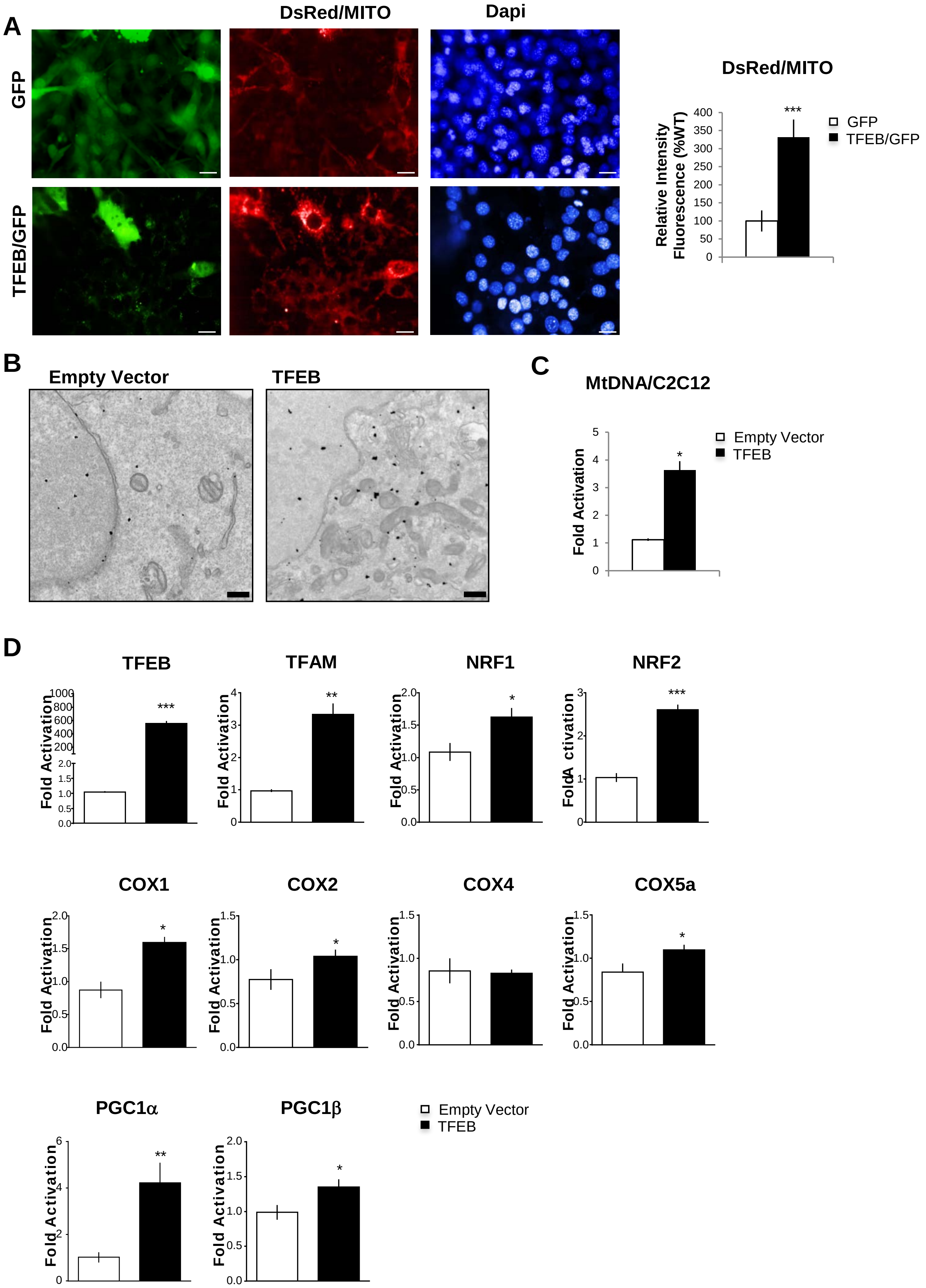
A
DsRed/MITO
Dapi
GFP
TFEB/GFP
DsRed/MITO
Relative Intensity Fluorescence (%WT)
400
350
300
250
200
150
100
50
0
***
GFP
TFEB/GFP
B
Empty Vector
TFEB
C
MtDNA/C2C12
Fold Activation
5
4
3
2
1
0
*
Empty Vector
TFEB
D
TFEB
TFAM
NRF1
NRF2
Fold Activation
1000
800
600
400
200
2.0
1.5
1.0
0.5
0.0
***
**
*
***
COX1
COX2
COX4
COX5a
PGC1α
PGC1β
Empty Vector
TFEB

Figure S2. TFEB induces mitochondrial biogenesis in C2C12 cells. Related to Figure 1. (A) Confocal images of C2C12 cells co-transfected with DsRed/MITO and with *CMV-GFP* or *TFEB-GFP*. Nuclei were stained with DAPI (blue). Mitochondria are shown in red. DAPI and DsRed were excited with 405 (UV) and 561 laser lines, respectively. Images were acquired on a Perkin Elmer Opera automated confocal microscope. The scale bars represent 10 µm. Percentage of DsRed/MITO intensity was calculated using Prism software (see Materials and Methods for details) Mean ± SE, n = 3; **p<0.01. (B) Electron microscopy analysis of C2C12 cells that overexpress *TFEB*-3XFlag compared with control cells. The scale bars represent 500 nm. (C) Quantitative MtDNA analysis, by Rt-PCR, confirmed an increase of mtDNA when TFEB is overexpressed in muscle cells compared with WT condition. Error bars represent mean ± SE for n=3; **p<0.01. (D) Expression analysis of genes related to mitochondrial biogenesis and functions in C2C12 cells transfected by empty vector (white bar) or *TFEB*-3XFlag (black bar). Error bars represent mean ± SE for n=3; *p<0.05, **p<0.01, ***p<0.001.

Figure S3.

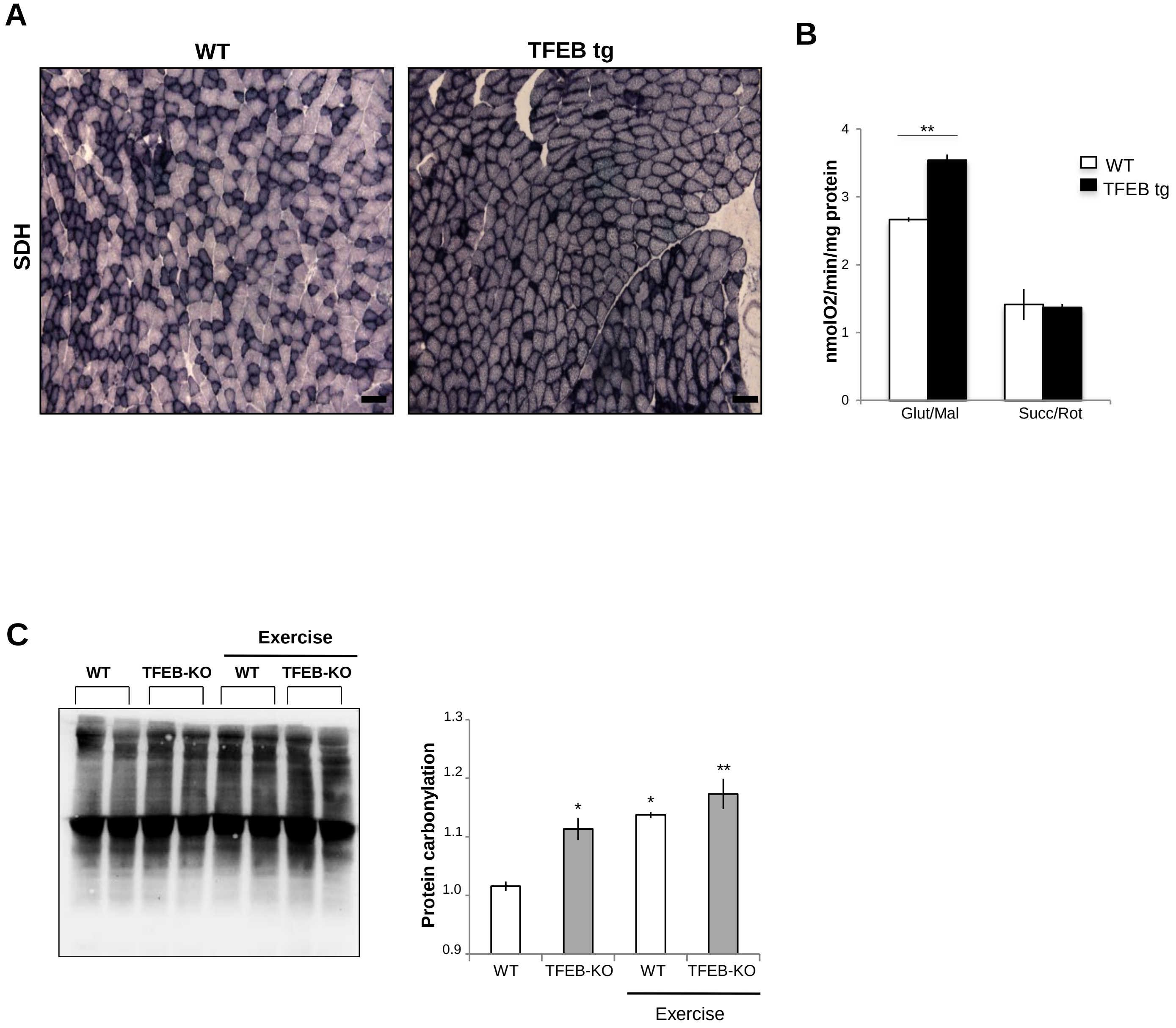
A
WT
TFEB tg
SDH
B
nmolO2/min/mg protein
4
3
2
1
0
**
WT
TFEB tg
Glut/Mal
Succ/Rot
C
Exercise
WT
TFEB-KO
WT
TFEB-KO
Protein carbonylation
1.3
1.2
1.1
1.0
0.9
*
*
**
WT
TFEB-KO
WT
TFEB-KO
Exercise

Figure S3. TFEB increases respiratory chain activity and glycogen synthesis in skeletal muscle. Related to Figure 2. Succinate dehydrogenase (SDH) staining of cryosections from TFEB transgenic mice compared with control mice. TFEB was induced for 2 weeks in 5 months old mice. The scale bars represent 100 nm. (B) Respiration of mitochondria from muscles of TFEB transgenic and control mice. TFEB significantly increased oxygen consumption rate when glutamate/malate were used as substrates. Data are presented as mean $\pm$ SE; n=3; $^{**}p < 0.01$. (C) Overall protein carbonylation of WT and *TFEB*-KO muscles revealed by Oxyblot. A representative immunoblot for carbonylated proteins is depicted on the left; densitometric quantification of the carbonylated proteins is shown in the graph on the right. *TFEB*- KO mice show higher carbonylated than WT in both sedentary condition and after exercise. n =4; $^{*}p < 0.05$). Values are mean $\pm$ SE for n = 4; *p < protein 0.05.

Figure S4.

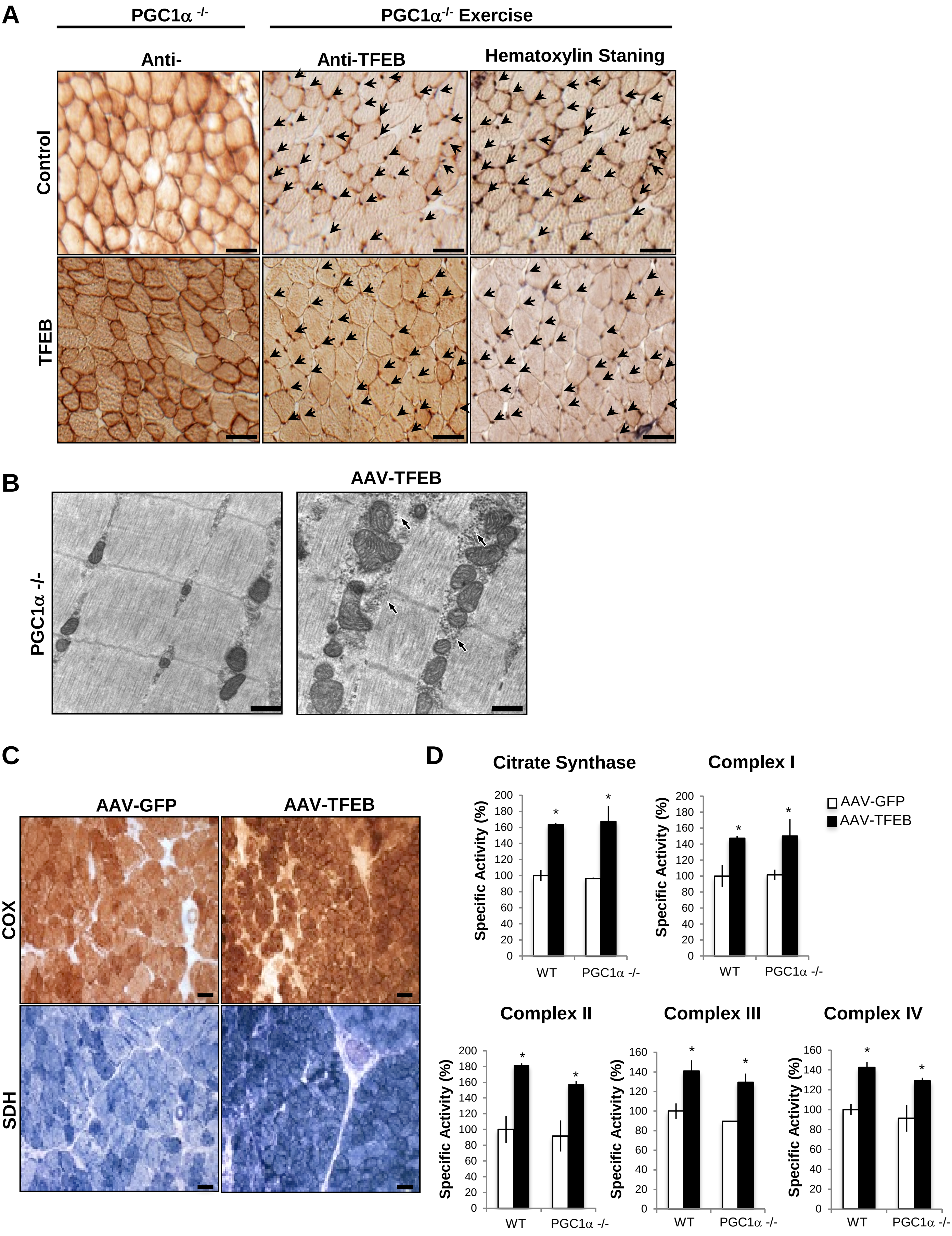
A
PGC1α -/-
PGC1α-/- Exercise
Anti-
Anti-TFEB
Hematoxylin Staning
Control
TFEB
B
AAV-TFEB
PGC1α -/-
C
AAV-GFP
AAV-TFEB
COX
SDH
D
Citrate Synthase
Complex I
Complex II
Complex III
Complex IV
Specific Activity (%)
WT
PGC1α -/-
AAV-GFP
AAV-TFEB

Figure S4. Exercise induces TFEB expression and nuclear localization in PGC1α-/- muscle. Related to Figure 3. (A) TFEB immunohistochemical analysis of PGC1α-/- muscles from sedentary and exercised mice. The muscles were infected by intramuscular injection of AAV2.1 control and AAV2.1-*TFEB* virus. Gastrocnemius muscle cryosections were immunostained by using anti-TFEB antibody and counter- stained with hematotoxylin for nuclei detection. Control means endogenous TFEB in muscles infected with control virus. TFEB means the transgene TFEB in muscles infected with AAV-*TFEB*. Arrows indicate exercise-induced TFEB nuclear localization. The scale bars represent 100 nm. (B-D) PGC1α is not required for mitochondrial biogenesis induced by TFEB. (B) EM of PGC1α-/- muscles that were infected with AAV2.1-*TFEB* or AAV2.1-*GFP*. The electron micrographs reveal an accumulation of mitochondria in *TFEB*-overexpressing muscle and a granular staining consistent with glycogen b- particles. The scale bars represent 500 nm. (C) COX and SDH staining of cryostatic sections from PGC1α-/- muscles transfected by AAV2.1-*GFP* or AAV2.1-*TFEB*. The scale bars represent 100 nm. (D) The mitochondrial respiratory chain activity in control and *TFEB*-overexpressing muscles. Data are expressed as percentage respect to the WT muscles infected with AAV-*GFP* control vector. Error bars represent mean ± SE for n=3; *$p<0.05$.

Figure S5.

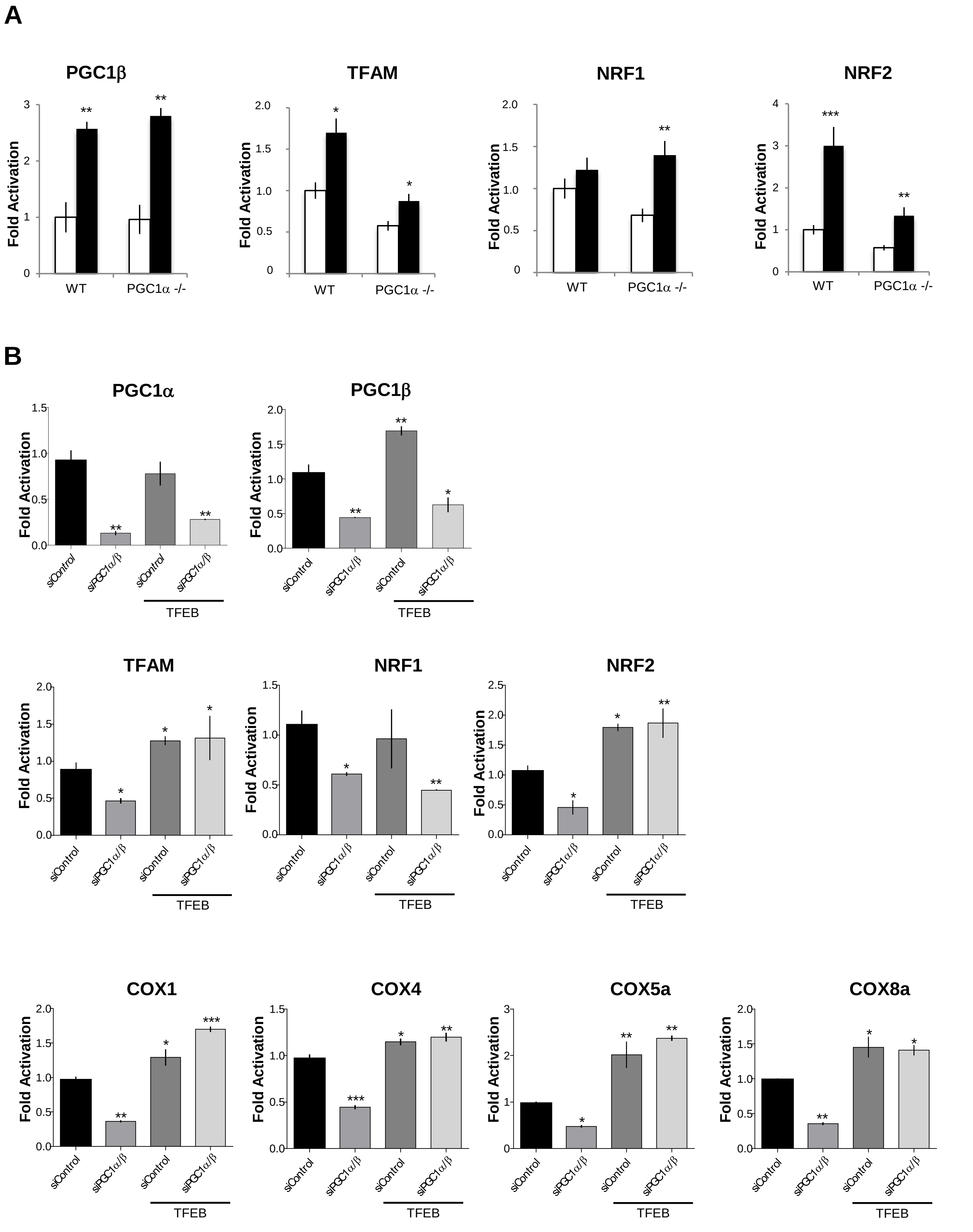
A
PGC1β
TFAM
NRF1
NRF2
Fold Activation
WT
PGC1α -/-
***
**
*
B
PGC1α
PGC1β
TFAM
NRF1
NRF2
COX1
COX4
COX5a
COX8a
siControl
siPGC1α/β
TFEB

Figure S5. TFEB controls mitochondrial biogenesis and activity by a PGC1α and PGC1β- independent mechanism. Related to Figure 3. (A) Expression analysis of genes related to mitochondrial biogenesis in WT and PGC1α-/- muscles that were transfected by AAV2.1-*GFP* (white bar) or AAV2.1-*TFEB* (black bar). Error bars represent mean ± SE for n=3; *p<0.05, **p<0.01, ***p<0.001. (B) Expression analysis of genes related to mitochondrial biogenesis and mitochondrial respirator chain in Hela cells silenced for PGC1α PGC1β and transfected with *TFEB* or empty vector. Error bars represent mean ± SE for n=3; *p<0.05, **p<0.01, ***p<and 0.001.

Figure S6.

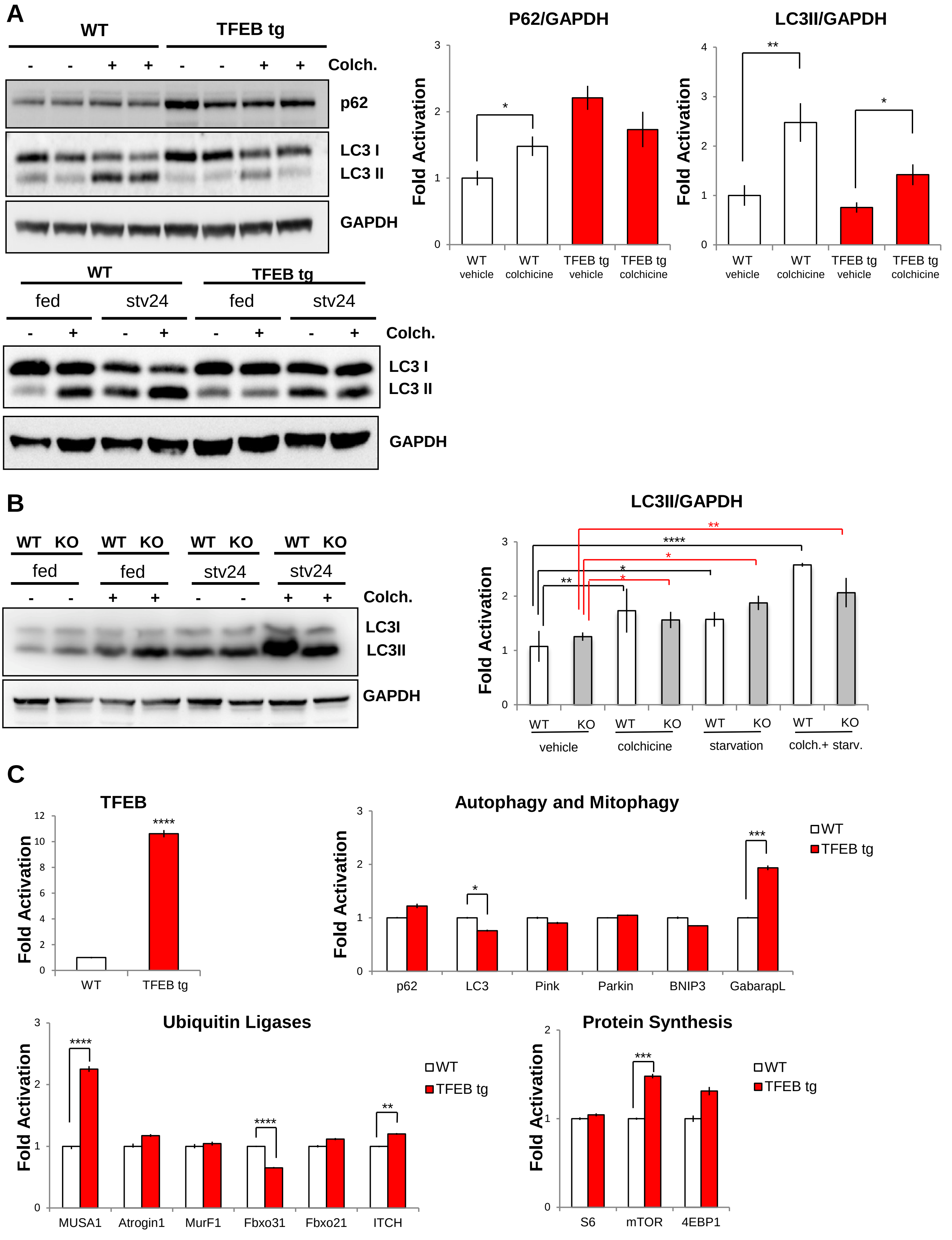
A
WT
TFEB tg
Colch.
p62
LC3 I
LC3 II
GAPDH
P62/GAPDH
LC3II/GAPDH
Fold Activation
WT vehicle
WT colchicine
TFEB tg vehicle
TFEB tg colchicine
fed
stv24
B
WT KO
LC3I
LC3II
vehicle
colchicine
starvation
colch.+ starv.
C
TFEB
Autophagy and Mitophagy
p62
LC3
Pink
Parkin
BNIP3
GabarapL
Ubiquitin Ligases
MUSA1
Atrogin1
MurF1
Fbxo31
Fbxo21
ITCH
Protein Synthesis
S6
mTOR
4EBP1

Figure S6. Autophagy flux is not changed by TFEB-overexpression or deletion. **Related to Figure 5.** (A) Upper panel: representative blots of p62 and LC3 from protein lysates of gastrocnemius muscles of WT and TFEB transgenic (tg) animals. Mice were treated with colchicine (Colch.) or vehicle for autophagy flux measurements. The densitometric quantification is depicted on the right panel. Lower panel: immunoblot analysis of LC3 from protein lysates of gastrocnemius muscles from fed or 24 h fasted TFEB tg or control mice. GAPDH was used as loading control. (B) Representative blot and densitometric quantification of LC3 from protein lysates from gastrocnemius muscles of fed and starved *TFEB-KO* or controls mice. Mice were treated with colchicine or vehicle for autophagy flux measurements. GAPDH was used as loading control. Data are representative of four independent experiments. (C) Quantitative RT-PCR of TFEB, autophagy/mitophagy (*p62/SQSTM1*, *LC3*, *Pink*, *Parkin*, *Bnip3* and *Gabarapl*), atrophy-related ubiquitin ligases (*MusA1*, *Atrogin1*, *MuRF1*, *Fbxo31*, *Fbxo21 and Itch*) and protein synthesis (S6, mTOR and *4EBP1*) transcripts. Data are normalized to GAPDH and expressed as fold increase of control animals. *n*=4 muscles in each group Values are mean ± SE *p<0.05, ***p<0.001, ****p<0.0001.

Figure S7.

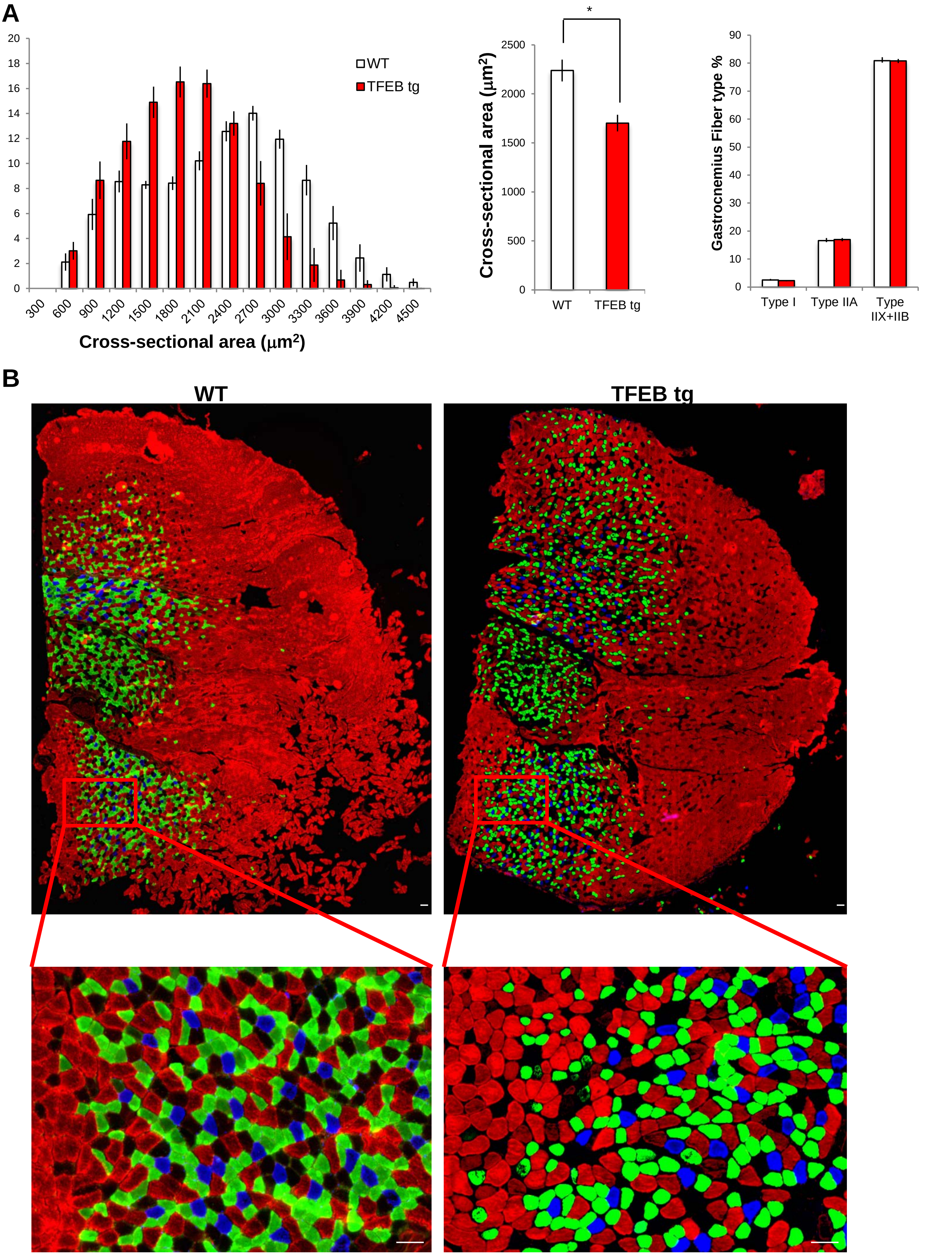

A
20
18
16
14
12
10
8
6
4
2
0
300
600
900
1200
1500
1800
2100
2400
2700
3000
3300
3600
3900
4200
4500
Cross-sectional area (μm²)
WT
TFEB tg
*
2500
2000
1500
1000
500
0
Cross-sectional area (μm²)
WT
TFEB tg
90
80
70
60
50
40
30
20
10
0
Gastrocnemius Fiber type %
Type I
Type IIA
Type IIX+IIB
B
WT
TFEB tg

Figure S7. TFEB-overexpression mouse muscles modifies fiber size but does not affect MHC expression. Related to Figure 5. (A) Frequency histograms showing the distribution of myofibers cross-sectional areas (μm2) from gastrocnemius muscles of inducible muscle-specific TFEB transgenic (black bars) and control male mice (white bars). (B) Average cross-sectional area of myofibers from grastrocnemius muscles of TFEB tg and control male mice. Values are shown as means ± SE *p<0.05, from 4 muscles in each group. Percentage of type I and type II fibers in WT and TFEB tg was calculated using ImageJ 1.47 software (see Materials and Methods for details). Values are shown as means ± SE *p<0.05, from 3 muscles in each group. (c) Cryosections of Gastrocnemius muscle were immunostained for the different MHCI, MHCIIa, and MHCIIb myosins. Type I (blue), type IIA (green), type IIB (red) and type IIX (unstained) fibers are depicted. The scale bars represent 100 nm.

Supplemental Tables

Table S1. TFEB overexpression muscle dataset. Related to Figure 1. Complete list of differentially expressed genes in TFEB overexpression muscle microarray dataset (GSE62975). The genes significantly (FDR<0.05; FoldChange ≥1.5) up-regulated and down-regulated are highlighted in red and in green, respectively.
(supplied as Excel file: Supplemental Table 1.xlsx).

Table S2. TFEB-KO muscle dataset. Related to Figure 1. Complete list of differentially expressed genes in TFEB-KO muscle microarray dataset (GSE62976). The genes significantly (FDR<0.05) up-regulated and down-regulated are highlighted in red and in green, respectively.
(supplied as Excel file: Supplemental Table 2.xlsx).

Table S3. TFEB controls lipid and glucose metabolism in muscle. Related to Figure 1. List of Lipid and Glucose Metabolism related terms related to the GOEA induced by TFEB overexpression and inhibited by TFEB-KO. For each term the genes significantly induced by TFEB overexpression (Supplemental Table BPALL_UP_OverTFEB) and inhibited by TFEB-KO (Supplemental Table BPALL_DOWN_KO-TFEB) are reported in table.
(supplied as Excel file: Supplemental Table 3.xlsx).

Tables S4. Mitochondrial genes up-regulated in TFEB overexpression muscle. Related to Figure 1. List of 38 genes significantly up-regulated in TFEB overexpression muscle dataset, with a main localization in the mitochondrion.

**Gene Symbol**	**Gene Title**
Abat	4-aminobutyrate aminotransferase
Arl2bp	ADP-ribosylation factor-like 2 binding protein
Bak1	BCL2-antagonist/killer 1
Bik	BCL2-interacting killer
Bid	BH3 interacting domain death agonist
Cds2	CDP-diacylglycerol synthase (phosphatidate cytidylyltransferase) 2
Dna2	DNA replication helicase 2 homolog (yeast)
Htra2	HtrA serine peptidase 2
Rab11fip5	RAB11 family interacting protein 5 (class I)
Acsl6	acyl-CoA synthetase long-chain family member 6
Aldh18a1	aldehyde dehydrogenase 18 family, member A1
Alas1	aminolevulinic acid synthase 1
Cpt1a	carnitine palmitoyltransferase 1a, liver
Cidea	cell death-inducing DNA fragmentation factor, alpha subunit-like effector A
Ckmt1	creatine kinase, mitochondrial 1, ubiquitous
Cyb5r3	cytochrome b5 reductase 3
Cox6a1	cytochrome c oxidase, subunit VI a, polypeptide 1; predicted gene 7795
Gyk	glycerol kinase
Gatm	glycine amidinotransferase (L-arginine:glycine amidinotransferase)
Hspa1b	heat shock protein 1B; heat shock protein 1A; heat shock protein 1-like
Hk1	hexokinase 1
Me2	malic enzyme 2, NAD(+)-dependent, mitochondrial
Msto1	misato homolog 1 (Drosophila)
Mrpl39	mitochondrial ribosomal protein L39
Maoa	monoamine oxidase A
Nos1	nitric oxide synthase 1, neuronal
Ppif	peptidylprolyl isomerase F (cyclophilin F)

Pmaip1	phorbol-12-myristate-13-acetate-induced protein 1
Sfxn1	sideroflexin 1
Ugt1a9, Ugt1a2, Ugt1a6a, Ugt1a7c, Ugt1a10, Ugt1a1, Ugt1a5, Ugt1a6b	UDP glucuronosyltransferase 1 family
Cox7c	similar to cytochrome c oxidase, subunit VIIc; predicted gene 3386
Slc25a5	solute carrier family 25 (mitochondrial carrier, adenine nucleotide translocator), member 5; similar to ADP/ATP translocase 2 (Adenine nucleotide translocator 2) (ANT 2) (ADP,ATP carrier protein 2)
Slc25a24	solute carrier family 25 (mitochondrial carrier, phosphate carrier), member 24
Slc25a36	solute carrier family 25, member 36
Slc25a37	solute carrier family 25, member 37
Slc25a45	solute carrier family 25, member 45
Tmlhe	trimethyllysine hydroxylase, epsilon
Tdrd7	tudor domain containing 7

Table S5. Mitochondrial genes down-regulated in TFEB-KO muscle. Related to Figure 1. List of 73 genes significantly inhibited in the TFEB-KO muscle dataset, with a main localization in the mitochondrion.

**Gene Symbol**	**Gene Title**
Abcb8	ATP-binding cassette, sub-family B (MDR/TAP), member 8
Aco2	aconitase 2, mitochondrial
Acox1	acyl-Coenzyme A oxidase 1, palmitoyl
Acsl1	acyl-CoA synthetase long-chain family member 1
Adhfe1	alcohol dehydrogenase, iron containing, 1
Adsl	adenylosuccinate lyase
Ak3	adenylate kinase 3
Aldh2	aldehyde dehydrogenase 2, mitochondrial
Araf	v-raf murine sarcoma 3611 viral oncogene homolog
Atp5a1	ATP synthase, H+ transporting, mitochondrial F1 complex, alpha subunit, isoform 1
Bckdhb	branched chain ketoacid dehydrogenase E1, beta polypeptide; similar to 3-methyl-2-oxobutanoate dehydrogenase
Bdh1	3-hydroxybutyrate dehydrogenase, type 1
Ccdc123	similar to RIKEN cDNA 2610507L03 gene; coiled-coil domain containing 123
Coq5	coenzyme Q5 homolog, methyltransferase (yeast)
Cox15	COX15 homolog, cytochrome c oxidase assembly protein (yeast)
Cox8b	cytochrome c oxidase, subunit VIIIb
Cpt1b	carnitine palmitoyltransferase 1b, muscle
Cs	citrate synthase
Dhrs4	dehydrogenase/reductase (SDR family) member 4
Dhx30	DEAH (Asp-Glu-Ala-His) box polypeptide 30
Dnaja3	DnaJ (Hsp40) homolog, subfamily A, member 3
Dnajc11	DnaJ (Hsp40) homolog, subfamily C, member 11
Echs1	enoyl Coenzyme A hydratase, short chain, 1, mitochondrial
Ecsit	ECSIT homolog (Drosophila)

Fth1	ferritin heavy chain 1
Gatm	glycine amidinotransferase (L-arginine:glycine amidinotransferase)
Gcdh	glutaryl-Coenzyme A dehydrogenase
Gfm2	G elongation factor, mitochondrial 2
Got2	glutamate oxaloacetate transaminase 2, mitochondrial
Gpam	glycerol-3-phosphate acyltransferase, mitochondrial
Gpd1	glycerol-3-phosphate dehydrogenase 1 (soluble)
Gpd2	glycerol phosphate dehydrogenase 2, mitochondrial
Ide	insulin degrading enzyme
Idh2	isocitrate dehydrogenase 2 (NADP+), mitochondrial
Idh3a	isocitrate dehydrogenase 3 (NAD+) alpha
Immt	inner membrane protein, mitochondrial
Isca1	iron-sulfur cluster assembly 1 homolog (S. cerevisiae)
Ivd	isovaleryl coenzyme A dehydrogenase
Ldhb	lactate dehydrogenase B; predicted gene 5514
Mavs	mitochondrial antiviral signaling protein
Mccc2	methylcrotonoyl-Coenzyme A carboxylase 2 (beta)
Mdh2	malate dehydrogenase 2, NAD (mitochondrial)
Mfn1	mitofusin 1
Mrpl15	mitochondrial ribosomal protein L15
Myl10	myosin, light chain 10, regulatory
Ndufa10	NADH dehydrogenase (ubiquinone) 1 alpha
Ndufv1	NADH dehydrogenase (ubiquinone) flavoprotein 1
LOC100045796, Nfs1	nitrogen fixation gene 1 (S. cerevisiae)
Nos1	nitric oxide synthase 1, neuronal
Nudt19	nudix (nucleoside diphosphate linked moiety X)-
Oxct1	3-oxoacid CoA transferase 1
Parl	presenilin associated, rhomboid-like
Pdk2	pyruvate dehydrogenase kinase, isoenzyme 2
Pln	phospholamban
Pnkd	paroxysmal nonkinesiogenic dyskinesia
Ppif	peptidylprolyl isomerase F (cyclophilin F)
Prdx6	Peroxiredoxin-6 (Antioxidant protein 2)
Pxmp2	peroxisomal membrane protein 2
Rfk	riboflavin kinase
Rhot2	ras homolog gene family, member T2
Rtn4ip1	reticulon 4 interacting protein 1
Sdhb	succinate dehydrogenase complex, subunit B, iron
Arts (Sept4)	septin 4
Shmt1	serine hydroxymethyltransferase 1 (soluble)
Slc25a15	solute carrier family 25 (mitochondrial carrier
Suclg2	succinate-Coenzyme A ligase, GDP-forming, beta
Supv3l1	suppressor of var1, 3-like 1 (S. cerevisiae)
Tmem186	transmembrane protein 186
Tufm	predicted gene 9755; Tu translation elongation
Ung	uracil DNA glycosylase
Uqcrc1	ubiquinol-cytochrome c reductase core protein 1

Table S6. CLEAR sites on mitochondrial genes. Related to Figure 2. Position and sequence of the CLEAR sites (i.e. the consensus TFEB binding-site) in the promoter region (1Kb upstream the TSS in the human and Mouse promoters) of mitochondrial genes down-regulated in TFEB-KO muscle.
(supplied as Excel file: Supplemental Table 6.xlsx).

Table S7. CLEAR sites in promoter region of genes related to mitochondria biogenesis and glucose metabolism. Related to Figure 2 and Figure 7. Position and sequence of TFEB binding site on the GLUT1, GLUT4, nNOS, TFAM, NRF1 and NRF2 promoter.
(supplied as Excel file: Supplemental Table 7.xlsx).

Supplemental Experimental Procedures

Cell culture, Plasmids and Transfection Reagent.
C2C12 and HeLa cells were purchased from ATCC and cultured in DMEM media supplemented with 10% fetal bovine serum, 200 μM L-Glutamine, 100 μM sodium pyruvate, in 5% CO_2 at 37° C. Cells were transfected with pDsRed2-Mito (Clontech) and Human full-lenght *TFEB*-GFP or *TFEB*3XFlag, both constructs were previously described (Martina et al., 2012; Sardiello et al., 2009). Cells were transfected using TransIT®-LT1 Transfection Reagent (MirusBio) according to the protocol from the manufacturers. Cells were silenced with PGC1α siRNA oligonucleotides (Thermo Dhamacon, L-005111-00), PGC1β siRNA oligonucleotides (Thermo Dhamacon, L-008556-00) or non-targeting siRNAs (Thermo Dhamacon, D-001810-10-05), by direct or reverse transfection, using Lipofectamine RNAiMAX reagent (Invitrogen) according to the protocol from the manufacturer. siRNA-transfected cells were collected after 48 h, if not otherwise stated.

Real-time PCR.
For mtDNA content analysis, SYBR Green real-time PCR was performed using primers specific to a mouse mtDNA region in the ND1 gene and primers specific to RNaseP, a single copy gene taken as a nuclear gene reference, as described (Viscomi et al., 2009). For the analysis of transcripts, total RNA was extracted from liquid nitrogen snap frozen muscle by Trizol, according to the manufacturer's instructions (Invitrogen, Carlsbad, CA, USA). Of total RNA, 2 μg was treated with RNase free-DNase and retrotranscribed using the ''cDNA cycle'' kit (QuantiTect Reverse Transcription Kit - QIAGEN). Approximately 2–5 ng of cDNA was used for real-time PCR assay (SYBR®Green PCR Master Mix Applied Biosystem) using primers specific for amplification of several genes. Autophagic, Lipid Metabolism and Glucose Metabolism gene-specific primers are listed below. Fold change values were calculated using the ΔΔCt method. An unpaired *t*-test was used to calculate statistical significance.

List of murine primers for RT-PCR

Gene Name	Primer Sequence
TCFEB F	GCAGAAGAAAGACAATCACAA
TCFEB R	GCCTTGGGGATCAGCATT
GLUT1 F	AGCAGCAAGAAGGTGACG
GLUT1 R	CACGGAGAGAGACCAAAGC
GLUT4 F	CCGCGGCCTCCTATGAGATACT
GLUT4 R	AGGCACCCCGAAGATGAGT
NOS1 F	CCAAAGCAGAGATGAAAGACACA
NOS1 R	TCTTGGTAGGAGACTGTTTGC
PGC1alpha F	AGCCGTGACCAGTGACAACGAG
PGC1alpha R	GCTGCATGGTTCTGAGTGCTAAG
GAPDH F	TGCACCACCAACTGCTTAGC
GAPDH R	TCTTCTGGGTGGCAGTGATC
TBC1D1 F	GTCCCGGGGTAATAAAGCCA
TBC1D1 R	TTGTCACCCATGGACAGCTC
PGC1-beta F	ACTGAAAGAGGCCCAGCAGA
PGC1-beta R	ATTGGAAGGGCCTTGTCTGA
PPAR-alpha F	CCTACGCTTGGGGATGAAGA
PPAR-alpha R	CCCCATTTCGGTAGCAGGTA
DLAT F	TGTTTCATCGGTGTTGAGTCTG
DLAT R	TGCTAATGATGTGCCCTTGTG
CRAT F	GGAGAAGAGAGCCAGTCCAG
CRAT R	AGATAATCCTCCCACCGCTG

CPT1b F	GACAGTACCCTCCTTCCACC
CPT1b R	ACCAGCAAGAACAGGGTAAGA
CS F	GACCCTCGCTATTCCTGTCA
CS R	AGTTCATCTCCGTCATGCCA
COX8B F	CGAAGTTCACAGTGGTTCCC
COX8B R	GCTGGAACCATGAAGCCAAC
MDH2 F	TGAACGGGAAGGAAGGAGTC
MDH2 R	AATGCCCAGGTTCTTCTCCA
CYC1 F	GGCTCCTCCCATCTACACAG
CYC1 R	CTGACCACTTATGCCGCTTC
ATP5A1 F	GACAGTACCCTCCTTCCACC
ATP5A1 R	ACCAGCAAGAACAGGGTAAGA
SDHA F	TTACCTGCGTTTCCCCTCAT
SDHA R	AAGTCTGGCGCAACTCAATC
NRF1 F	CAGACACGTTTGCTTCGGAAA
NRF1 R	CCCACTCGCGTCGTGTACT
NRF2 F	GATCAGGCGACATGTTAACGTT
NRF2 R	AGAGCCCAGTCAAACCCTTTC
HK2 F	TGGGTTTCACCTTCTCGTTC
HK2 R	TGGACTTGAACCCCTTAGTCC
HK1 F	TGGTGGAAAAGATCCGAGAG
HK1 R	TTTGGTGCATGATTCTGGAG
COX1 F	TGCTAGCCGCAGGCATTACT
COX1 R	CGGGATCAAAGAAAGTTGTGTTT
COX2 F	CAGGCCGACTAAATCAAGCAA
COX2 R	GAGCATTGGCCATAGAATAATCCT
COX4 F	TCACTGCGCTCGTTCTGAT
COX4 R	CGATCGAAAGTATGAGGGATG
COX5a F	TCATCCAGGAACTTAGACCAACT
COX5a R	AGTCCTTAGGAAGCCCATCG
TFAM F	AGATATGGGTGTGGCCCTTG
TFAM R	AAAGCCTGGCAGCTTCTTTG
CD36 F	CCAAATGAAGATGAGCATAGGACAT
CD36 R	GTTGACCTGCAGTCGTTTTGC
ND1 F	CATAAGCCTGCGTCAGATCA
ND1 R	CCTGTGTTGGGTTGACAGTG
RNasiP F	GCCTACACTGGAGTCCGTGCTACT
RNasiP R	CTGACCACACACGAGCTGGTAGAA
PPAR gamma F	CGTGCAGCTACTGCATGTGA
PPAR gamma R	GGGTGGGACTTTCCTGCTAA
PPAR delta F	ATGGGGGACCAGAACACAC
PPAR delta R	GGAGGAATTCTGGGAGAGGT
GYS F	TTGGAAGACTGGGAGGATGA
GYS R	CATTCATCCCCTGTCACCTT
PYGM F	CTACGAAAAGGACCCCAG
PYGM R	CATGGTGTTCTGCAGTGTC
Atrogin F	GCAAACACTGCCACATTCTCTC
Atrogin R	CTTGAGGGGAAAGTGAGACG
Murf1 F	ACCTGCTGGTGGAAAACATC
Murf1 R	ACCTGCTGGTGGAAAACATC

Fbxo31 F	GTATGGCGTTTGTGAGAACC
Fbxo31 R	AGCCCCAAAATGTGTCTGTA
Fbxo21 F	TCAATAACCTCAAGGCGTTC
Fbxo21 R	GTTTTGCACACAAGCTCCA
ITCH F	CCACCCACCCCACGAAGACC
ITCH R	CTAGGGCCCGAGCCTCCAGA
p62 F	CCCAGTGTCTTGGCATTCTT
p62 R	AGGGAAAGCAGAGGAAGCTC
LC3 F	CACTGCTCTGTCTTGTGTAGGTTG
LC3 R	TCGTTGTGCCTTTATTAGTGCATC
PINK F	TGGAATATCTCGGCAGGTTC
PINK R	CGGATGATGTTAGGGTGTGG
Parkin F	CCATCTTGCTGGGACGA
Parkin R	GCCTGTTGCTGGTGATCA
BNIP3 F	TTCCACTAGCACCTTCTGATGA
BNIP3 R	GAACACCGCATTTACAGAACAA
GabarapL F	CATCGTGGAGAAGGCTCCTA
GabarapL R	ATACAGCTGGCCCATGGTAG
mTOR F	GAGAAGGGTATGAATCGAGATGA
mTOR R	CCCATGAGGTCTTTGCAGTA
S6 F	AGCCCAGGACCAAAGCACCCAA
S6 R	TCCTGGCGCTTTTCTTTGGCTTCC
MUSA1 F	TCGTGGAATGGTAATCTTGC
MUSA1 R	CCTCCCGTTTCTCTATCACG

List of human primers for RT-PCR

PGC1 alpha F	TAGTCCTTCCTCCATGCCTG
PGC1 alpha R	TGCATGGTTCTGGGTACTGA
PGC1 beta F	CTCTTCACCCTGCCACTCC
PGC1 beta R	ACCTCGCACTCCTCAATCTC
TFAM F	TTAGAAGAATTGCCCAGCGT
TFAM R	TTCTTTATATACCTGCCACTCCG
NRF1 F	CTTGTACCATCACAGACTGTAGT
NRF1 R	ACGGCAGAATAATTCACTTGGG
NRF2 F	GCCCCTGTTGATTTAGACGG
NRF2 R	GTTTGGCTTCTGGACTTGGA
COX1 F	ACCCTAGACCAAACCTACGC
COX1 R	TGTATGCATCGGGGTAGTCC
COX4 F	CAAGCGAGCAATTTCCACCT
COX4 R	GTCACGCCGATCCATATAAGC
COX5a F	TGCCTGGGAATTGCGTAAAG
COX5a R	ATTTAACCGTCTGCATGCCC
COX8a F	GAGGGGAAGCTTGGGATCAT
COX8a R	CCTGTAGGTCTCCAGGTGTG
ND1 F	CCTAAAACCCGCCACATCTA
ND1 R	GCCTAGGTTGAGGTTGACCA

Generation of muscle-specific TFEB Knockout and inducible muscle-specific transgenic mice and in vivo experimental procedures.

Mice bearing *Tcfeb*-floxed alleles (Tcfeb$^{f/f}$) (Settembre et al., 2012) were crossed with transgenic mice expressing Cre either under the control of a Myosin Light Chain 1 fast promoter (*MLC1f-Cre*) (Bothe et al., 2000). Genomic DNA isolated from Tcfeb$^{f/f}$ mice was subjected to PCR analysis. Cre-mediated recombination was confirmed by PCR with genomic DNA from gastrocnemius muscles using the primers Cre forward: 3′-CACCAGCCAGCTATCAACTCG-5′ and Cre reverse: 3′-TTACATTGGTCC AGCCACCAG-5′.

The Pgc1α Knockout mouse line was obtained from Jackson Laboratory (Bruce Spiegelman, Dana- Farber Cancer Institute) (Lin et al., 2004). The muscle-specific Pgc1α Knockout mouse were generated by crossing the Pgc1α floxed mice from Jackson Laboratory (Bruce Spiegelman, Dana-Farber Cancer Institute) with *MLC1f-Cre* transgenic mice.

To generate inducible muscle-specific TFEB transgenic animals, *Tcfeb*3XFlag cDNA was inserted after a CAGCAT cassette [chicken actin promoter (CAG) followed by chloramphenicol acetyltransferase (CAT) cDNA flanked by 2 loxP sites] and used to generate transgenic mice (Baylor College of Medicine transgenic core) (Settembre et al., 2011). Mice were then crossed with transgenic mice expressing Cre-ER driven by human skeletal actin promoter (HSA). Tamoxifen-induced Cre LoxP recombination was activated by IP injection of tamoxifen-containing for 3 days. Muscles were collected 2 weeks after the tamoxifen treatment. Cre-negative littermates, also receiving tamoxifen treatment, were used as controls. Adult mice (3 to 5 months-old) of the same sex and age were used for each individual experiment.

All mice used were males and maintained in a C57BL/6 strain background. For all experiments involving *TFEB*-KO mice, the control mice were mice Tcfeb$^{f/f}$ that did not carry the *MLC1f-Cre* transgene. Standard food and water were given ad libitum. Mice were maintained in a temperature - and humidity-controlled animal-care facility, with a 12 hr light/dark cycle and free access to water and food (Standard Diet, Mucedola, Italy). All experiments were performed on 3- to 5-month-old male (28–32*g*), mice of the same sex and age were used for each individual experiment. *In vivo TFEB* overexpression experiments were performed by i.m. injection of a total dose of 10^{11} GC of AAV2/1 vector preparation. Muscles were removed 21 days after injection and frozen in liquid nitrogen for subsequent analyses. For fasting experiments, control animals were fed *ad libitum*; food pellets were removed from the cages of the fasted animals. All procedures were formerly approved by the Italian Public Health, Animal Health, Nutrition and Food Safety, Italian Ministry of Health, (D.M. N°75/2014-B).

Western Blot Analysis and Antibodies.

For western blot the following antibodies were used: anti-GAPDH (cat. sc-32233) anti-nNOS (cat. sc-648) from Santa Cruz Biotechnology; anti-NDUFA9 (cat.459100), anti-SDHA (cat. 459200), anti-COX5a (cat. 459120), from Invitrogen; anti-Gys (cat. 3886), anti-pGys (cat. 3891) , anti-AMPK (cat.2532), anti-pAMPK (cat. 2531), anti-pACC (cat. 3661), anti-AKT (cat. 4691) from Cell Signaling; anti-GLUT1(cat. ab40084) from Abcam; anti-LC3B (cat. L7543), anti-p62 (cat. P0067) from Sigma Aldrich.

Biochemical Analysis of Mitochondrial Respiratory Chain Complex.

Complex I activity was measured using 50 μg mitochondrial protein in a reaction containing 250 mM sucrose, 2 mM EDTA, 100 mM Tris–HCl pH 7.4, between 10 and 100 μM decyl-ubiquinone and 2 mM KCN at 30° C. Reactions were started by the addition of 50 μM NADH and oxidation followed at 340 nm for 2 minutes. Rotenone was used to block any rotenone insensitive activity.

Complex II activity was measured by incubating 40 μg of mitochondrial protein at 30° C in a reaction mix containing 50 mM KH2PO4, pH 7.4, 20 mM succinate, 2 μg/mL antimycin A, 2 μg/mL rotenone, 2 mM KCN and 50 μM 2,6-dichlorophenolindophenol (DCPIP). Reactions were initiated by adding 50μM DB to the reaction and measuring the change in absorbance at 600 nm for 2 min.

Complex III activity is measured by incubating 20 μg mitochondrial proteins at 30° C in a reaction mix containing 250 mM sucrose, 50 mM Tris–HCl, 1 mM EDTA, 50 μM cytochrome c and 2 mM KCN. Decylubiquinol (DBH2) was used an electron donor. The reaction was started by the addition of 50 μM DBH2 and the change in absorbance of cytochrome c at 550 nm was followed for 2 min. 5 μg of antimycin A were added to duplicate reactions to measure any antimycin A-insensitive activity. Complex IV activity was measured by adding 10 μg mitochondria to a mix containing 10 mM KH2PO4, pH 7.2 and between 5 and 50 μM ferrocytochrome c. The rate of oxidation of ferrocytochrome c was recorded for 2 min (extinction coefficient of 27.2 mM− 1 cm− 1). Citrate synthase activity was measured at 30° C by using 10 μg mitochondria to a

reaction mixture containing 125 mM Tris–HCl, 100 μM DTNB (5,5′-dithiobis(2-nitrobenzoic acid) and 300 μM acetyl coenzyme A. The reaction is initiated by the addition of 500 μM oxaloacetate, and DTNB reduction at 412 nm measured for 2 min. The Mitochondrial respiratory chain activities were expressed as nmoles/min/mg of protein.
For the quantitative determination of Adenosine 5'triphosphate (ATP) the muscle gastrocnemius samples stored in liquid nitrogen were homogenized in sterile water and analyzed according to the manufacturer's instructions of ATP Bioluminescent Assay Kit (Sigma-Aldrich).

Protein Carbonyls Detection.
Carbonylation of muscle proteins was detected by using the OxyBlot Protein Oxidation Detection Kit from Millipore (Masiero et al., 2009). Spot detection and quantification was carried out using the UVP VisionWorksLS software.

Acute Exercise, Training and Fatigue Experiments
Mild exercise was performed by let mice run on the treadmill for 1 hour at 15 cm/sec speed for 4 days. Training was performed by increasing the speed of 5 cm/sec every week for a total of 7 weeks. The starting speed was the 50% of the maximal speed (exhaustion test) that corresponded to 25cm/sec and the final speed reached at the end of the training period was 55 cm/sec.
To measure muscle endurance, soleus muscles were dissected under a stereomicroscope and mounted between a force transducer (KG Scientific Instruments, Heidelberg, Germany) and a micromanipulator- controlled shaft in a small chamber in which oxygenated Krebs solution was continuously circulated and temperature maintained at 25°C. The stimulation voltage was optimized, and the length of the soleus was increased until force development during tetanus was maximal. The responses to a single stimulus (twitch) or to trains of stimuli at various rates producing unfused or fused tetani were recorded. In order to induce muscle fatigue, muscles were stimulated for 500ms at 100Hz every 2 seconds (0,25 duty cycle). Induction of fatigue ex-vivo is known to be performed at a duty cycle between 0,1 and 0,5 (Allen et al., 2008).

Mitochondrial Assays
Mitochondria from mouse skeletal muscle were isolated as described (Frezza et al., 2007). The rate of mitochondria oxygen consumption was measured at 3 0 ° C in an incubator chamber with a Clark/type O2 electrode filled with 2ml of incubation medium (125 mM KCl, 10 mM Pi.Tris, 20 mM Tris-HCl, 0,1 mM EGTA, ph 7,2). All measured were performed using mitochondria (0,2 mg mitochondrial protein/ml) incubated either with glutamate (5 mM)/ malate (2,5 mM) or /and succinate (5 mM) as substrates, in the presence (state 3) and in absence (state 4) of 100 mM ADP (Frezza et al., 2007).

Autophagic flux quantification
We monitored autophagic flux in fed and 24 h starving mice by using colchicine as previously described (Milan et al., 2015). Briefly muscle specific transgenic TFEB and *TFEB*-KO mice were treated with 0,4 mg/kg colchicine or vehicle by i.p. injection and starved. The treatment was repeated at 15 h prior to muscle harvesting.

Morphological Analysis.
For SDH activity, 8µm thick sections from frozen tissue were collected on coverslips. The reaction mix contained: 5 mM phosphate buffer pH 7.4, 5 mM EDTA, 1 mM KCN, 0.2 mM Phenazine methosulfate (PMS), 50 mM Succinic acid, 1.5 mM Nitro blue tetrazolium (NBT). NBT was the electron acceptor with PMS served as intermediate electron donor to NBT. Sections were incubated for 20 min at 37° C. For control sections, sodium malonate (10 mM) was added to the incubation medium. For Cytochrome-c Oxidase the sections were incubated 1 hr at 37° C. The reaction mix contained: 5 mM phosphate buffer pH 7.4, 0.1% 3,3'-Diaminobenzidine (DAB), 0.1% Cytochrome c, 0.02% catalase. Histochemical analyses were performed as described (Sciacco and Bonilla, 1996).
Cross-sectional area was measured using ImageJ software in at least 400 fibres and compared with the area of age-matched control. The fibre diameter was calculated as caliper width, perpendicular to the longest chord of each myofibre. The total myofibre number was calculated from entire muscle section based on assembled mosaic image (×20 magnification). (Fibre typing was determined by immunofluorescence using combinations of the following monoclonal antibodies: BA-D5 that recognizes type 1 MyHC isoform and SC-71 for type 2A MyHC isoform. Images were captured using a Leica DFC300-FX digital charge-coupled device camera by using Leica DC Viewer software, and morphometric analyses were made using the software ImageJ 1.47 version.

Immunohistochemistry

Frozen muscle serial sections (7 μm) were fixed in 4% paraformaldehyde in PBS for 10 min, washed twice in PBS (Sigma-Aldrich) for 5 min at room temperature (RT), and incubated in 0.03% H_2O_2 in Methanol for 10 min at RT. After washing in PBS, sections were incubated in blocking solution (10% normal goat serum, 5% BSA in PBS) for 1h at RT. Anti-TFEB mAb (Bethil Laboratories) was added separately to each serial section for 16 h at 4°C. Sections were washed twice in PBS, incubated with biotin-conjugated goat anti-rabbit IgG1, 5 μg/ml with 1% normal goat serum and 0,5% BSA in PBS) for 2 h at 4°C, washed twice, incubated with HRP-conjugated streptavidin (*Nova*RED substrate kit for peroxidase) (Vector Laboratories), following the manufacturer instructions, then rinsed in distilled water for 5 min, and counterstained with hematoxylin. Sections were prepared from independent muscle specimens from 3 mice for group; and 3–4 sections for each muscle were analyzed.

Electron microscopy.

Small pieces of muscle tissue were fixed in a mixture of 2% paraformaldehyde and 1% Glutaraldehyde prepared in 0.2 M Hepes. Then samples were post-fixed in a mixture of osmium tetroxide and potassium ferrocyanide, dehydrated in ethanol and propyleneoxide and embedded in epoxy resin as described previously (Polishchuk et al., 2014). Thin 65 nm sections were cut using a Leica EM UC7 ultramicrotome. EM images were acquired using a FEI Tecnai-12 electron microscope (FEI, Eindhoven, Netherlands) equipped with a VELETTA CCD digital camera (Soft Imaging Systems GmbH, Munster, Germany). Morphometric analysis of number and size of mitochondria was performed using iTEM software (Olympus SIS, Germany). Number of mitochondria was counted using the same magnification within 100 μm square field of view. For each experiment, between 154 and 196 individual mitochondrial diameters were measured from 3 mice per group.

Tissue metabolites quantification

Muscle free fatty acids were extracted as follows: briefly, pulverized muscle was homogenized in PBS, then extracted using chloroform/methanol (2:1), dried overnight and re-suspended in a solution of 60% butanol 40% Triton X-114/methanol (2:1). Measurements were normalized to protein content in the initial homogenate by DC protein assay (Bio-Rad). The quantitative determination of glycogen amount was performed according to the manufacture's instruction of the Glycogen Colorimetric/Fluorometric Assay Kit (BioVision). For the quantitative determination of keton bodies, the muscle gastrocnemius samples stored in liquid nitrogen were homogenized and analyzed according to the manufacturer's instructions of β-hydroxybutyrate colorimetric assay kit (Vinci-Biochem). β- Hydroxybutyrate concentration is determined by a coupled enzyme reaction, which results in a colorimetric (450 nm) product, proportional to the β-Hydroxybutyrate present in muscle.

Whole Body Indirect Calorimetry.

Mice were individually housed in the chamber with a 12-h light/12-h dark cycle in an ambient temperature of 22–24° C. VO_2 and VCO_2 rates were determined under Oxymax system settings as follows: air flow, 0.6 l/min; sample flow, 0.5 l/min; settling time, 6 min; and measuring time, 3 min. The system was calibrated against a standard gas mixture to measure O_2 consumed (VO_2, ml/kg/h) and CO_2 generated (VCO_2, ml/kg/h). Energy expenditure (EE), oxygen consumption ($\dot{V}o_2$) and carbon dioxide production ($\dot{V}co_2$) during running was determined as described by Shemesh (Shemesh et al., 2014). Briefly, the mice were acclimatized to a treadmill (Columbus Instruments) by running at 10 m/min for 15 min over three consecutive days. On the fourth day, groups of mice were run at 10 m/min, progressing to 15 m/min. Energy expenditure, oxygen consumption ($\dot{V}o_2$) and carbon dioxide production ($\dot{V}co_2$) were recorded during running interval, and the total distance run to exhaustion was determined.

In Vivo Assessment of Insulin Action and Glucose Metabolism.

The 2-h EU clamp was conducted with a prime continuous infusion of human insulin (4 mill units/kg/min) and a variable infusion of 25% glucose to maintain glucose at <150 mg/dl. Insulin stimulated whole body glucose metabolism was estimated using a prime continuous infusion of [3-^{3}H] glucose (10 μCi bolus, 0.1 μCi/min; PerkinElmer Life Sciences). To determine the rate of basal glucose turnover, [3-^{3}H] glucose (0.05 μCi/min) was infused for 2 h (basal period) before starting the EU clamp, and a blood sample was taken at the end of this basal period. To assess insulin-stimulated tissue-specific glucose uptake, 2-deoxy-d-[1-^{14}C] glucose (PerkinElmer Life

Sciences) was administered as a bolus (10 μCi) 75 min after the start of the clamp. Blood samples were taken at 80, 90, 100, 110, and 120 min after the start of the EU clamp. At the end of the EU clamp, different muscle groups, adipose tissue, and liver were rapidly dissected and frozen at -80° C for analysis. For the determination of plasma [3-^{3}H]glucose and 2-deoxy-d-[1-^{14}C]glucose concentrations, plasma was deproteinized with $ZnSO_4$ and $Ba(OH)_2$, dried to remove $^{3}H_2O$, resuspended in water, and counted in scintillation fluid (Ultima Gold; Packard Instrument Co.). For the determination of tissue 2-deoxy-d-[1-^{14}C]glucose (2-DG)-6-phosphate (2-DG-6-P) content, tissue samples were homogenized, and the supernatants were subjected to an ion-exchange column to separate 2-DG-6-P from 2-DG, as described previously. The radioactivity of ^{3}H in tissue glycogen was determined by digesting tissue samples in KOH and precipitating glycogen with ethanol as previously described. Muscle glycogen synthesis was calculated as muscle [^{3}H] glycogen content divided by the area under the plasma [^{3}H] glucose-specific activity profile.

Microarray hybridization.

Total RNA (3 μg) was reverse transcribed to single-stranded cDNA with a special oligo (dT) 24 primer containing a T7 RNA promoter site, added 3' to the poly-T tract, prior to second strand synthesis (One Cycle cDNA Synthesis Kit by Affymetrix, Fremont, CA, USA). Biotinylated cRNAs were then generated, using the GeneChip IVT Labeling Kit (Affymetrix). Twenty micrograms of biotinylated cRNA was fragmented and 10 μg hybridized to the Affymetrix GeneChip Mouse 430A-2 microarrays for 16 hours at 45° C using an Affymetrix GeneChip Fluidics Station 450 according to the manufacturer's standard protocols.

For determine the changes in muscle transcriptome induced by *TFEB* overexpression, total RNA was extracted from muscle from three mice injected with an adenoviral vector that expresses mouse *Tcfeb* (AAV2.1-*TFEB*) and muscle from three mice injected with AAV2.1-*GFP* used as control.

For the analysis of *Tcfeb* knock-out (KO) mice muscle-specific total RNA was extracted from the muscle of three *TFEB*-KO mice 3 month old as compared to three WT mice used as control.

Microarray data processing.

The data discussed in this publication have been deposited in NCBIs Gene Expression Omnibus (GEO)(Edgar et al., 2002) and are accessible through GEO Series accession number GSE62975 (*TFEB*_overexpression muscle dataset) and GSE62976 (*TFEB*-KO_muscle dataset). The two muscle studies (GSE62975 and GSE62976) are part of the SuperSeries GSE62980.

The SuperSeries has been named: Expression data from mice after knockout or overexpression of *Tcfeb* in muscle. Low-level analysis to convert probe level data to gene level expression was performed using Robust Multiarray Average (RMA) implemented using the RMA function of the Bioconductor project (Gentleman et al., 2004a, b).

Statistical analysis of differential gene expression.

For each gene, a Bayesian t-test (Cyber-t) (Baldi and Long, 2001) was used on RMA normalized data to determine if there was a significant difference in expression between mice overexpressing *TFEB* (*TFEB* overexpression muscle mice) versus not-injected mice used as control and *Tcfeb* knock-down (KO) mice muscle-specific versus WT mice. P-value adjustment for multiple comparisons was done with the False Discovery Rate (FDR) of Benjamini-Hochberg (Klipper-Aurbach et al., 1995). The threshold for statistical significance chosen for both muscle microarray datasets analysis was FDR<0.05; a further filtering was performed for *TFEB* overexpression muscle dataset (GSE62975) by selection genes with an absolute Fold Change ≥1.5 for both increased (up-regulated genes) and decreased (down-regulated genes) expression levels.

Microarray data analysis.

Gene Ontology Enrichment Analysis (GOEA) (Dennis et al., 2003) was performed for each microarray dataset on the up-regulated and down-regulated gene lists, separately, by using the DAVID online tool (DAVID Bioinformatics Resources 6.7) restricting the output to all Biological Process terms (BP_ALL) and to all Cellular Compartments terms (CC_ALL). The threshold for statistical significance of GOEA was FDR≤10% and Enrichment Score ≥1.5. 73 out of 461 genes significantly inhibited in the *TFEB*-KO_muscle dataset were mainly localized in the mitochondrion (GO:0005739) as shown in the Table S5. Promoter analysis to isolate potential TFEB binding sites (i.e. CLEAR motifs) was also performed on a region of 1kb up-stream the TSS of the human and mouse promoter sequences of the 73 Mitochondrial genes; 52 out of the total 73 Mitochondrial genes contain at least one CLEAR motif in the human or mouse promoter, or in both (Table S6, 73 Mitochondrial_DW_*TFEB*-KO_CLEAR). 38 out of 1512 genes significantly induced in the *TFEB* overexpression muscle dataset were localized in the

mitochondrial part (GO:0044429; refer to Table S4). In Figure 1A the Lipid and Glucose Metabolism related terms induced by *TFEB* overexpression and inhibited by *TFEB*-KO were selected from BPALL_UP in *TFEB* overexpression and from BPALL_DOWN in *TFEB*-KO, respectively. For each term the genes significantly induced by *TFEB* overexpression and inhibited by *TFEB*-KO are reported in table Table S3 Lipid and Glucose Metabolism.

Accession codes:

Primary accessions Gene Expression Omnibus

The two muscle studies (GSE62975 and GSE62976) are part of the SuperSeries **GSE62980**. The SuperSeries has been named: Expression data from mice after knockout or overexpression of *Tcfeb* in muscle.

GSE62975: Expression data from injected mice overexpressing *Tcfeb* specifically in muscle

GSE62976: Expression data from *Tcfeb* KO mice specifically in muscle

Supplemental References

Allen, D.G., Lamb, G.D., and Westerblad, H. (2008). Skeletal muscle fatigue: cellular mechanisms. Physiological reviews *88*, 287-332.

Baldi, P., and Long, A.D. (2001). A Bayesian framework for the analysis of microarray expression data: regularized t -test and statistical inferences of gene changes. Bioinformatics *17*, 509-519.

Bothe, G.W., Haspel, J.A., Smith, C.L., Wiener, H.H., and Burden, S.J. (2000). Selective expression of Cre recombinase in skeletal muscle fibers. Genesis *26*, 165-166.

Dennis, G., Jr., Sherman, B.T., Hosack, D.A., Yang, J., Gao, W., Lane, H.C., and Lempicki, R.A. (2003). DAVID: Database for Annotation, Visualization, and Integrated Discovery. Genome Biol *4*, P3.

Edgar, R., Domrachev, M., and Lash, A.E. (2002). Gene Expression Omnibus: NCBI gene expression and hybridization array data repository. Nucleic Acids Res *30*, 207-210.

Frezza, C., Cipolat, S., and Scorrano, L. (2007). Organelle isolation: functional mitochondria from mouse liver, muscle and cultured fibroblasts. Nature protocols *2*, 287-295.

Gentleman, R.C., Carey, V.J., Bates, D.M., Bolstad, B., Dettling, M., Dudoit, S., Ellis, B., Gautier, L., Ge, Y., Gentry, J., et al. (2004a). Bioconductor: open software development for computational biology and bioinformatics. Genome Biol *5*, R80.

Gentleman, R.C., Carey, V.J., Bates, D.M., Bolstad, B., Dettling, M., Dudoit, S., Ellis, B., Gautier, L., Ge, Y., Gentry, J., et al. (2004b). Bioconductor: open software development for computational biology and bioinformatics. Genome biology *5*, R80.

Klipper-Aurbach, Y., Wasserman, M., Braunspiegel-Weintrob, N., Borstein, D., Peleg, S., Assa, S., Karp, M., Benjamini, Y., Hochberg, Y., and Laron, Z. (1995). Mathematical formulae for the prediction of the residual beta cell function during the first two years of disease in children and adolescents with insulin-dependent diabetes mellitus. Medical hypotheses *45*, 486-490.

Lin, J., Wu, P.H., Tarr, P.T., Lindenberg, K.S., St-Pierre, J., Zhang, C.Y., Mootha, V.K., Jager, S., Vianna, C.R., Reznick, R.M., et al. (2004). Defects in adaptive energy metabolism with CNS -linked hyperactivity in PGC-1alpha null mice. Cell *119*, 121-135.

Martina, J.A., Chen, Y., Gucek, M., and Puertollano, R. (2012). MTORC1 functions as a transcriptional regulator of autophagy by preventing nuclear transport of TFEB. Autophagy *8*, 903-914.

Masiero, E., Agatea, L., Mammucari, C., Blaauw, B., Loro, E., Komatsu, M., Metzger, D., Reggiani, C., Schiaffino, S., and Sandri, M. (2009). Autophagy is required to maintain muscle mass. Cell metabolism *10*, 507-515.

Milan, G., Romanello, V., Pescatore, F., Armani, A., Paik, J.H., Frasson, L., Seydel, A., Zhao, J., Abraham, R., Goldberg, A.L., et al. (2015). Regulation of autophagy and the ubiquitin-proteasome system by the FoxO transcriptional network during muscle atrophy. Nature communications *6*, 6670.

Polishchuk, E.V., Concilli, M., Iacobacci, S., Chesi, G., Pastore, N., Piccolo, P., Paladino, S., Baldantoni, D., van, I.S.C., Chan, J., et al. (2014). Wilson disease protein ATP7B utilizes lysosomal exocytosis to maintain copper homeostasis. Developmental cell *29*, 686-700.

Sardiello, M., Palmieri, M., di Ronza, A., Medina, D.L., Valenza, M., Gennarino, V.A., Di Malta, C., Donaudy, F., Embrione, V., Polishchuk, R.S., et al. (2009). A gene network regulating lysosomal biogenesis and function. Science *325*, 473-477.

Sciacco, M., and Bonilla, E. (1996). Cytochemistry and immunocytochemistry of mitochondria in tissue sections. Methods in enzymology *264*, 509-521.

Settembre, C., Di Malta, C., Polito, V.A., Garcia Arencibia, M., Vetrini, F., Erdin, S., Erdin, S.U., Huynh, T., Medina, D., Colella, P., et al. (2011). TFEB links autophagy to lysosomal biogenesis. Science *332*, 1429-1433.

Settembre, C., Zoncu, R., Medina, D.L., Vetrini, F., Erdin, S., Erdin, S., Huynh, T., Ferron, M., Karsenty, G., Vellard, M.C., et al. (2012). A lysosome-to-nucleus signalling mechanism senses and regulates the lysosome via mTOR and TFEB. The EMBO journal *31*, 1095-1108.

Shemesh, A., Wang, Y., Yang, Y., Yang, G.S., Johnson, D.E., Backer, J.M., Pessin, J.E., and Zong, H. (2014). Suppression of mTORC1 activation in acid-alpha-glucosidase-deficient cells and mice is ameliorated by leucine supplementation. American journal of physiology. Regulatory, integrative and comparative physiology *307*, R1251-1259.

Viscomi, C., Spinazzola, A., Maggioni, M., Fernandez-Vizarra, E., Massa, V., Pagano, C., Vettor, R., Mora, M., and Zeviani, M. (2009). Early-onset liver mtDNA depletion and late-onset proteinuric nephropathy in Mpv17 knockout mice. Human molecular genetics *18*, 12-26.
