# Supplementary material for: Transcription Factor EB Controls Metabolic Flexibility during Exercise
Source: Cell Metab. 2017 Jan 10;25(1):182–96. doi: 10.1016/j.cmet.2016.11.003 (PMC5241227; doi:10.1016/j.cmet.2016.11.003)
Supplement: Document S2. Article plus Supplemental Information [file mmc7.pdf]

Cell Metabolism

Article

Transcription Factor EB Controls Metabolic Flexibility during Exercise

Graphical Abstract

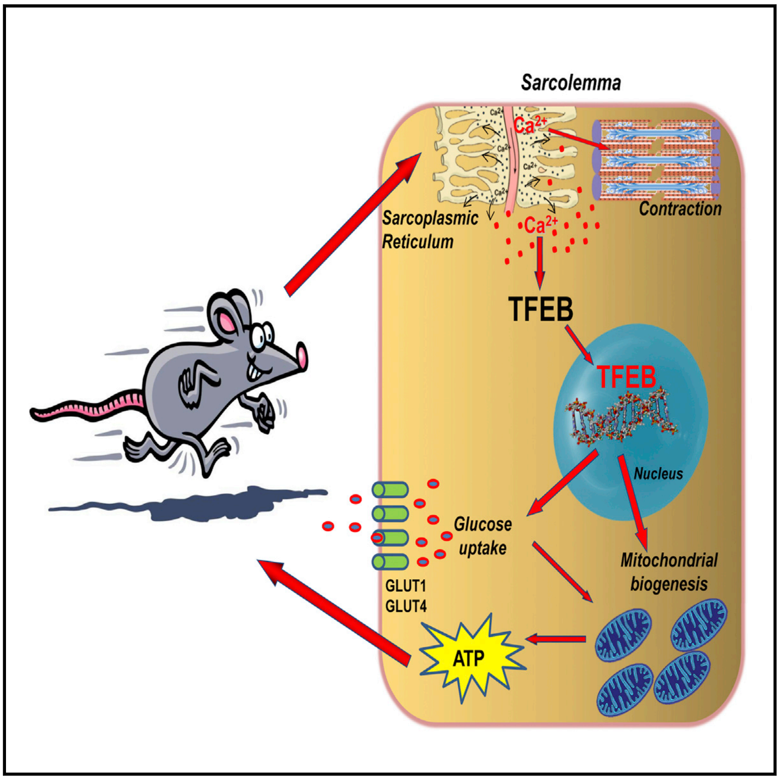

Highlights

- TFEB regulates mitochondrial biogenesis and function in muscle
- Glucose homeostasis in skeletal muscle requires TFEB
- The effects of TFEB on muscle metabolism are independent from PGC1α
- TFEB coordinates metabolic flexibility during physical exercise

Authors

Gelsomina Mansueto, Andrea Armani, Carlo Viscomi, ..., Massimo Zeviani, Marco Sandri, Andrea Ballabio

Correspondence

marco.sandri@unipd.it (M.S.),
ballabio@tigem.it (A.B.)

In Brief

Mansueto et al. show that TFEB acts as a central coordinator of skeletal muscle insulin sensitivity, glucose homeostasis, lipid oxidation, and mitochondrial function in the adaptive metabolic response to physical exercise in a PGC1α-independent manner.

Mansueto et al., 2017, Cell Metabolism 25, 182–196
January 10, 2017 © 2017 The Authors. Published by Elsevier Inc.
http://dx.doi.org/10.1016/j.cmet.2016.11.003

CellPress

Cell Metabolism
Article

Transcription Factor EB Controls Metabolic Flexibility during Exercise

Gelsomina Mansueto,[1] Andrea Armani,[2] Carlo Viscomi,[3,4] Luca D'Orsi,[1] Rossella De Cegli,[1] Elena V. Polishchuk,[1] Costanza Lamperti,[4] Ivano Di Meo,[4] Vanina Romanello,[2,5] Silvia Marchet,[4] Pradip K. Saha,[6] Haihong Zong,[7,8] Bert Blaauw,[2] Francesca Solagna,[2] Caterina Tezze,[2] Paolo Grumati,[9] Paolo Bonaldo,[9] Jeffrey E. Pessin,[8] Massimo Zeviani,[3,4] Marco Sandri,[1,2,5,13,*] and Andrea Ballabio[1,10,11,12,*]
[1]Telethon Institute of Genetics and Medicine (TIGEM), Via Campi Flegrei 34, 80078 Pozzuoli, Naples, Italy
[2]Department of Biomedical Science, University of Padova, Padova 35121, Italy
[3]MRC Mitochondrial Biology Unit, Cambridge CB2 0XY, UK
[4]Fondazione IRCCS Istituto Neurologico "C. Besta," 20133 Milan, Italy
[5]Venetian Institute of Molecular Medicine, Padova 35129, Italy
[6]Department of Molecular and Cellular Biology, Baylor College of Medicine, Houston, TX 77030, USA
[7]Hepatobiliary, Pancreatic, and Intestinal Research Institute, North Sichuan Medical College, Nanchong, Sichuan 637000, China
[8]Departments of Medicine and Molecular Pharmacology, Albert Einstein College of Medicine, Bronx, NY 10461, USA
[9]Department of Molecular Medicine, University of Padova, Padova 35121, Italy
[10]Medical Genetics, Department of Pediatrics, Federico II University, Via Pansini 5, 80131 Naples, Italy
[11]Department of Molecular and Human Genetics, Baylor College of Medicine, Houston, TX 77030, USA
[12]Jan and Dan Duncan Neurological Research Institute, Texas Children's Hospital, Houston, TX 77030, USA
[13]Lead Contact
*Correspondence: marco.sandri@unipd.it (M.S.), ballabio@tigem.it (A.B.)
http://dx.doi.org/10.1016/j.cmet.2016.11.003

SUMMARY

The transcription factor EB (TFEB) is an essential component of lysosomal biogenesis and autophagy for the adaptive response to food deprivation. To address the physiological function of TFEB in skeletal muscle, we have used muscle-specific gain- and loss-of-function approaches. Here, we show that TFEB controls metabolic flexibility in muscle during exercise and that this action is independent of peroxisome proliferator-activated receptor-γ coactivator1α (PGC1α). Indeed, TFEB translocates into the myonuclei during physical activity and regulates glucose uptake and glycogen content by controlling expression of glucose transporters, glycolytic enzymes, and pathways related to glucose homeostasis. In addition, TFEB induces the expression of genes involved in mitochondrial biogenesis, fatty acid oxidation, and oxidative phosphorylation. This coordinated action optimizes mitochondrial substrate utilization, thus enhancing ATP production and exercise capacity. These findings identify TFEB as a critical mediator of the beneficial effects of exercise on metabolism.

INTRODUCTION

Exercise elicits several beneficial effects by acting on mitochondrial content/function, fatty acid oxidation, and glucose homeostasis (Hawley, 2002; Holloszy and Coyle, 1984; Holloszy et al., 1998). Indeed, muscle activity is important to counteract disease progression in diabetes, obesity, and metabolic syndrome. The signaling pathways that control the contraction-mediated beneficial effects on mitochondria and glucose/lipid homeostasis are distinct from insulin signaling and mainly rely on AMPK and PGC1α. We have recently found that exercise leads to nuclear translocation of the helix-loop-helix leucine zipper transcription factor EB (TFEB) (Medina et al., 2015), an important regulator of lysosomal biogenesis and autophagy (Sardiello et al., 2009; Settembre et al., 2011). Upregulation of TFEB has been found in several tissues after food deprivation, including liver and skeletal muscle. We have previously shown that in liver, TFEB regulates genes involved in lipid catabolism, fatty acid oxidation, and ketogenesis (Settembre et al., 2013). Some of these effects are elicited by TFEB-mediated induction of PGC1α (Settembre et al., 2013), a transcriptional coactivator, which interacts with and enhances the activity of transcription factors involved in mitochondrial biogenesis, glucose homeostasis, and lipid oxidation (Kelly and Scarpulla, 2004; Puigserver et al., 1998).

In the presence of nutrients, TFEB is sequestered in the cytoplasm by mTORC1-mediated phosphorylation, whereas in nutrient-depleted conditions, mTORC1 is inactive and dephosphorylated TFEB translocates to the nucleus, where it induces the transcription of target genes (Martina et al., 2012; Roczniak-Ferguson et al., 2012; Settembre et al., 2012). The dephosphorylation of TFEB is mediated by the calcium-dependent phosphatase, calcineurin, which is necessary for TFEB activation (Medina et al., 2015). Importantly, exercise-dependent calcium influx activates calcineurin, which dephosphorylates TFEB, leading to nuclear localization. The calcineurin-mediated induction of TFEB is independent from mTORC1 activity, indicating that calcium-dependent signaling is a rate-limiting step of TFEB activation (Medina et al., 2015).

Previous studies implicated calcineurin in a variety of physiological processes, in particular in skeletal muscle adaptation

 © 2017 The Authors. Published by Elsevier Inc.
This is an open access article under the CC BY license (http://creativecommons.org/licenses/by/4.0/).

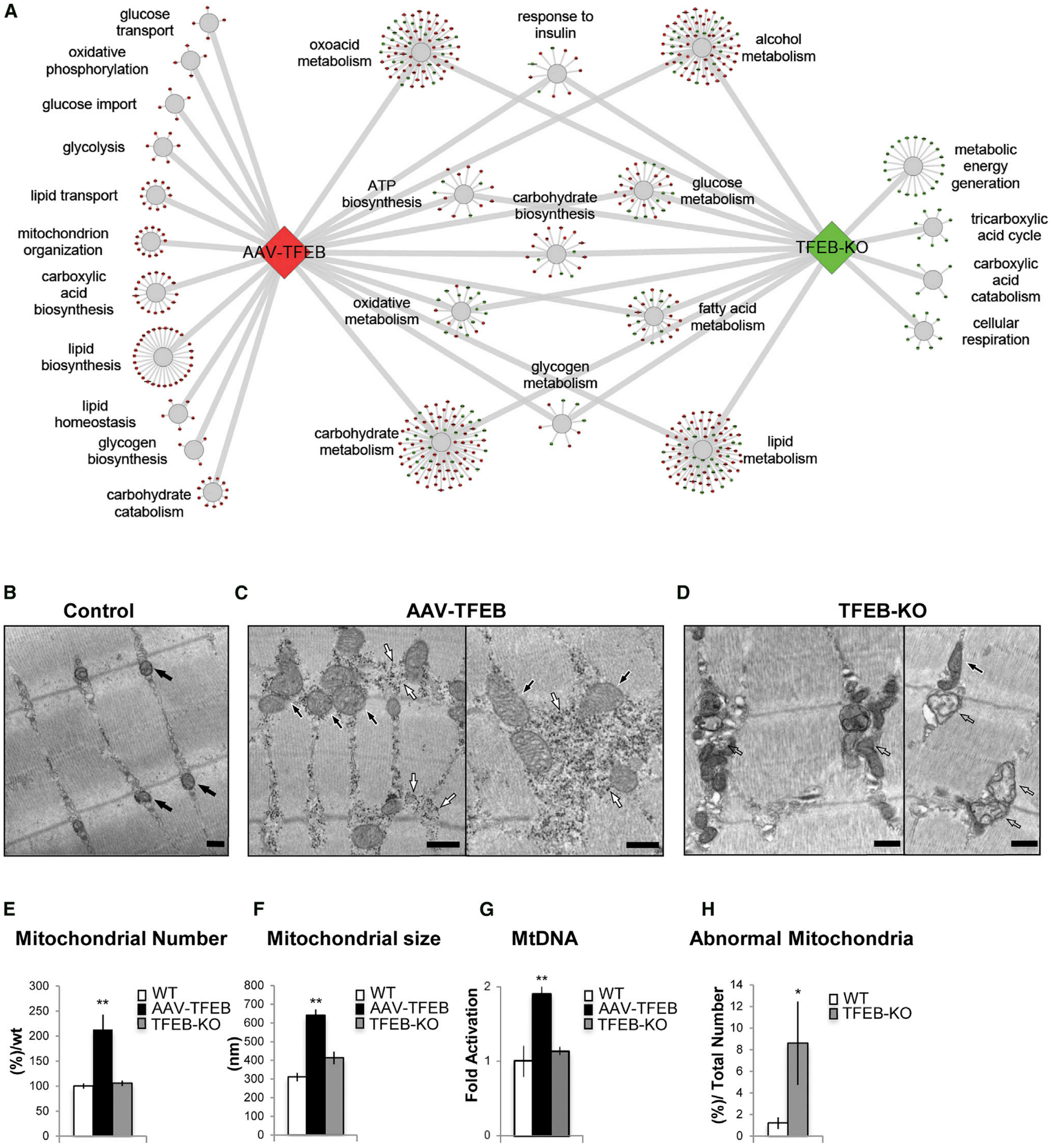

Figure 1. TFEB Induces Mitochondrial Biogenesis

(A) Genes involved in lipid and glucose metabolism that were affected by *TFEB* expression are shown in colored circles. The upregulated (red circles) and downregulated genes (green circles) are shown in *TFEB*-overexpressing and *TFEB* KO, respectively. The genes were divided into functional sub-categories.

(B–D) EM analysis of skeletal muscles from WT mice transfected with AAV2.1-*GFP* (B) and AAV2.1-*TFEB* (C), and from *TFEB* KO mice (D). Normal mitochondria are indicated by black arrows (B and C), while abnormal mitochondria in *TFEB* KO muscle (D) are indicated by empty arrows. Accumulation of glycogen is shown by white arrows in (C). The scale bars represent 500 nm.

(E and F) Morphometrical analyses of mitochondrial number (E) and size (F) in *TFEB*-overexpressing and *TFEB* KO muscles compared to controls. Error bars represent mean ± SE, n = 3; **p < 0.01.

(legend continued on next page)

to exercise (Gehlert et al., 2015). Muscle-specific transgenic mice that overexpress an activated form of calcineurin show increased glucose uptake, glycogen accumulation, and lipid oxidation (Long et al., 2007). Interestingly, calcineurin promotes the nuclear translocation of another family of transcription factors, NFAT, which, depending on the type of physical activity, modulate the expression of the different myosin isoforms (Calabria et al., 2009; Long et al., 2007; McCullagh et al., 2004).

Here we show that the calcineurin-TFEB axis plays a major role in the metabolic adaptations that occur during physical exercise. By using gain- and loss-of-function approaches, we show that TFEB regulates mitochondrial biogenesis and glucose uptake independently of PGC1α. Indeed, TFEB controls genes involved in glucose metabolism such as GLUT1 and GLUT4, hexokinase I and II, TBC1 domain family member 1 (TBC1D1), and glycogen synthase (GYS), leading to glycogen accumulation to sustain energy production during exercise.

RESULTS

Genome-wide Analyses Identified Glucose-Related and Mitochondrial Genes as Downstream Targets of TFEB

We previously demonstrated that TFEB promotes lipid catabolism in the liver and protects against diet-induced weight gain and insulin resistance (Settembre et al., 2013). Here we studied the physiological relevance of TFEB in skeletal muscle, an important insulin- and autophagy-dependent tissue (Grumati and Bonaldo, 2012; Mammucari et al., 2007; Masiero et al., 2009). Transcriptome analysis was performed by whole-genome gene expression profiling experiments (SuperSeries-GSE62980) in skeletal muscle from both *TFEB*-overexpressing and *TFEB* knockout (KO) mice. Overexpression of *Tcfeb*, the murine homolog of human *TFEB*, in muscle was achieved by means of intramuscular viral-mediated gene transfer using the adeno-associated virus (AAV) system. Adult mice were injected intramuscularly with either AAV2.1-CMV-*TFEB* or AAV2.1-CMV-*GFP* control vector and animals were sacrificed after 21 days, a time that allows efficient TFEB expression (Figure S1A, available online). Muscle-specific conditional *TFEB* KO mice were generated by crossing *Tcfeb* floxed (Settembre et al., 2013) with MLC1f-Cre transgenic mice (Bothe et al., 2000). Efficiency and specificity of the gene deletion were confirmed by quantitative real-time PCR analysis on multiple tissues (Figure S1B).

Overexpression of *TFEB* in muscle resulted in the upregulation of 1,514 genes and the downregulation of 1,109 genes (GSE62975), while genetic ablation of *TFEB* increased 496 genes and suppressed 458 genes (GSE62976). The up- or downregulated genes are highlighted in red and green, respectively, in Tables S1 and S2. To identify the main cellular compartments (CCs) and the principal biological process (BPs) for which the TFEB-dependent genes were enriched, we performed a gene ontology enrichment analysis (GOEA). The GOEA was performed on the lists of genes whose expression was either increased or decreased in transfected muscle or in the *TFEB* KO mice. Interestingly, several gene categories related to cellular metabolism, including lipid and glucose homeostasis, were found upregulated in *TFEB*-overexpressing muscle and downregulated in *TFEB* KO (Figure 1A; Table S3). Strikingly, genes involved in mitochondrial biogenesis were oppositely regulated by gain- and loss-of-function approaches. Indeed, 38 genes involved in mitochondrial function were induced in AAV2.1-*TFEB*-infected muscles (Table S4), while 73 genes were inhibited in *TFEB* KO muscles (Table S5).

To better identify the network of genes regulated by TFEB in muscle, we performed sequence analysis to identify putative TFEB target sites, previously referred to as CLEAR sites (coordinated lysosomal expression and regulation) (Palmieri et al., 2011), in the promoter regions of the downregulated genes in *TFEB* KO mice. Interestingly, we found that 79% of these genes contain a CLEAR sequence and are, therefore, potential direct targets of TFEB (Table S6).

TFEB Regulates Mitochondrial Biogenesis in Muscle

To examine potential effects of TFEB in mitochondrial function, we analyzed mitochondrial morphology in muscles overexpressing or lacking *TFEB*. Electron microscopy (EM) analyses showed a striking increase of mitochondrial density and volume in *TFEB*-overexpressing muscles (Figures 1B, 1C, 1E, and 1F). Interestingly, mitochondrial density and size were normal in the *TFEB* KO muscles (Figures 1E and 1F). Consistent with the EM data, increase of mitochondrial DNA (mtDNA) was found in TFEB transgenic muscles, while no differences were observed in *TFEB* KO muscles (Figure 1G). However, while the cristae shape, matrix density, and outer membrane morphology were normal in *TFEB*-overexpressing muscles (Figure 1C), abnormalities were found in approximately 10% of the mitochondria from *TFEB* KO muscles (Figures 1D and 1H). An increase in the number of mitochondria was also observed in C2C12 muscle cells transfected with *TFEB*-GFP, as detected by immunofluorescence confocal and confirmed by EM analyses (Figures S2A and S2B). Quantitative real-time PCR also revealed an increase of mtDNA content in *TFEB*-overexpressing cells (Figure S2C).

Importantly, quantitative real-time PCR analysis revealed that *TFEB* overexpression in muscle and in C2C12 cells induces the expression of many genes involved in mitochondrial biogenesis and function, including the master gene of mitochondrial biogenesis, PGC1α, a known direct target of TFEB (Settembre et al., 2013) (Figures 2A and S2D). Moreover, another PGC-1 family member, PGC1β, was also upregulated by *TFEB* overexpression. Consistently, we found a significant induction of peroxisome proliferator-activated receptor α (PPARα), PPARβ/δ, and PPARγ in *TFEB*-overexpressing muscles. However, *TFEB* deletion did not affect the expression of PGC1α/β and PPAR genes, with the exception of PPARα, which was downregulated. In order to elucidate the possible mechanisms underlying the induction of

(G) mtDNA analysis of muscles infected with AAV2.1-*TFEB* and from *TFEB* KO compared to WT mice. Quantitative real-time PCR of mtDNA copy numbers. Data are shown as mean ± SE, n = 3; $^{**}p < 0.01$.

(H) Quantification of abnormal mitochondria in *TFEB* KO gastrocnemius (GCN) muscles compared to WT muscles. Data are expressed as percentage of abnormal mitochondria on total mitochondria of 16 pictures (for detailed description of the criteria, see Experimental Procedures). Error bars represent mean ± SE; $^{*}p < 0.05$.

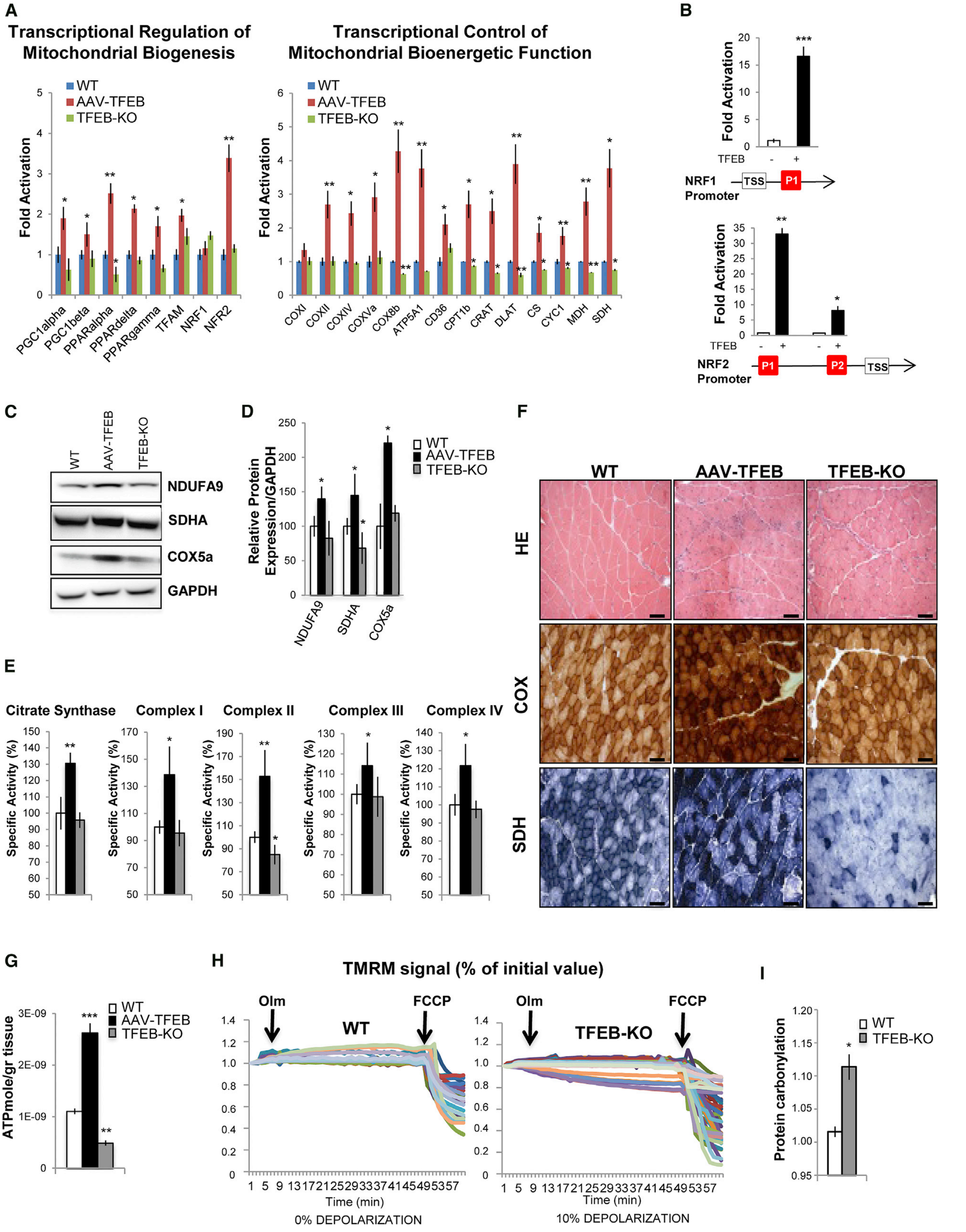

(legend on next page)

mitochondrial biogenesis observed in *TFEB*-transfected muscles, we examined the expression of nuclear respiratory factors 1 and 2 (NRF1and NRF2). The mRNA levels of NRF2 were increased, as well as NRF downstream genes, including mitochondrial transcription factor A (TFAM). Chromatin immunoprecipitation (ChIP) experiments confirmed the direct recruitment of TFEB on NRF1 and NRF2 promoters (Figure 2B), but not on TFAM promoter (data not shown). Finally, overexpression of *TFEB* in skeletal muscle increased the expression of mitochondrial enzymes. Subunits of the four respiratory chain complexes and the ATP synthase, as well as genes encoding electron transport and tricarboxylic acid cycle proteins, were induced by *TFEB* overexpression and were reduced by *TFEB* deletion (Figure 2A). Importantly, immunoblotting analyses confirmed the increase of complex I (NDUFA9), complex II (SDHA), and complex IV (COX5a) proteins in *TFEB*-transfected muscles (Figures 2C and 2D). *TFEB* deletion did not alter NDUFA9 and COX5a expression but significantly reduced the level of SDHA protein (Figures 2C and 2D). To better characterize the involvement of TFEB in mitochondrial respiration, we analyzed the specific activities of enzymes involved in oxidative phosphorylation. Biochemical analysis of muscle samples infected with AAV2.1-*TFEB* compared to wild-type (WT) muscles showed increase of citrate synthase, mitochondrial respiratory chain complex I (CI), CII, CIII, and CIV activities (Figure 2E). Consistent with the western blot analyses, *TFEB* deletion led to decrease of CII activity, the complex that contains the SDHA flavoprotein, while the other respiratory complexes had normal activities (Figure 2E). The changes in respiratory chain activities were corroborated by histochemical analyses for COX and SDH activity in AAV2.1-*TFEB*-transfected and *TFEB* KO muscles. Indeed, only SDH activity was greatly reduced in the absence of *TFEB*, while both COX and SDH were increased in *TFEB*-overexpressing muscles (Figure 2F). To further investigate the role of TFEB in mitochondrial function, we generated an inducible muscle-specific transgenic mouse line. Acute activation of TFEB by tamoxifen treatment in adult mice recapitulated the phenotype of AAV2.1-*TFEB* overexpression on mitochondria biogenesis (Figure S3). Consistent with the increase of SDH and respiratory chain complex activity, mitochondrial respiration was significantly enhanced by *TFEB* expression in adult transgenic muscles (Figures S3A and S3B).

We next assessed whether these TFEB-mediated changes of mitochondrial morphology and function had any impact on energy production. ATP levels were higher in *TFEB*-transfected muscles and lower in *TFEB*-deficient muscles compared to controls (Figure 2G). To understand the mechanisms underlying the significant decrease of ATP in *TFEB* KO muscles, we checked mitochondrial function in these animals. Fluorescent dyes, like tetramethylrhodamine methyl ester (TMRM), monitor the mitochondrial membrane potential ($\Delta\psi m$), the critical parameter that drives ATP production. Therefore, we checked the status of $\Delta\psi m$ in isolated adult fibers of WT and *TFEB* KO muscles. As expected, in control mice oligomycin-dependent inhibition of ATP synthase did not alter $\Delta\psi m$ (Figure 2H), and mitochondrial depolarization was achieved after membrane permeabilization by the protonophore carbonylcyanide-p-trifluoromethoxy phenylhydrazone (FCCP). Conversely, mitochondria of *TFEB* null fibers underwent a significant depolarization after oligomycin treatment (Figure 2G), suggesting that these fibers were at least in part relying on reverse activity of ATP synthase to preserve their membrane potential as a consequence of proton membrane leak. In addition, oxidative stress, revealed by protein carbonylation, was significantly higher in *TFEB* KO mice than controls (Figures 2I and S3C).

TFEB Regulates Mitochondrial Biogenesis in Skeletal Muscle through a PGC1α- and PGC1β-Independent Mechanism

Both PGC1α and PGC1β are master regulators of mitochondrial biogenesis and oxidative metabolism. However, recent findings suggest the presence of an independent pathway that regulates mitochondrial biogenesis during exercise (Rowe et al., 2012). Therefore, we examined whether TFEB is the missing sensor of physical activity that coordinates the metabolic responses independently of PGC1α. First, we checked expression and localization of endogenous TFEB in PGC1α KO mice before and after exercise. TFEB was expressed at lower levels and more cytosolic in *PGC1α* KO mice when compared to controls (Figures 3A and

Figure 2. TFEB Regulates Mitochondrial Function

(A) Quantitative real-time PCR of genes related to mitochondrial biogenesis and function in muscles of WT (blue bar), *TFEB*-overexpressing (red bar), and *TFEB* KO (green bar) mice. Data were normalized for GAPDH and expressed as fold induction relative to the WT. Data are expressed as mean ± SE, n = 3; *p < 0.05, **p < 0.01.

(B) ChIP analysis in muscles from *TFEB*-overexpressing and WT mice. The CLEAR elements of NRF1 and NRF2 promoters are depicted as red boxes. TSS (transcriptional start site) indicates the first codon. The histograms show the amount of immunoprecipitated DNA as detected by quantitative real-time PCR assay. Data represent mean ± SE of three independent experiments; *p < 0.05, **p < 0.01, ***p < 0.001.

(C) Western blots of mitochondrial respiratory chain proteins. Blot is representative of four independent experiments.

(D) Densitometric quantification of blots shown in (B). Data are shown as means ± SE (n = 4); *p < 0.05.

(E) Mitochondrial respiratory chain activity in muscles of WT, AAV2.1-*TFEB* transfected, and *TFEB* KO mice. Data are expressed as a percentage relative to the WT. Data are expressed as mean ± SE, n = 4; *p < 0.05, **p < 0.01.

(F) H&E, succinate dehydrogenase (*SDH*), and cytochrome oxidase (*COX*) staining of AAV2.1-GFP, AAV2.1-*TFEB* transfected, and muscles from *TFEB* KO mice. The scale bars represent 100 μm. Magnification, 20×.

(G) ATP production rate per gram of tissue from *TFEB*-overexpressing and *TFEB* KO mice compared to controls. Data are shown as means ± SE (n = 5); **p < 0.01, ***p < 0.001.

(H) Mitochondrial membrane potential of myofibers isolated from FDB muscles of WT and *TFEB* KO mice. Where indicated, 6 μM oligomycin or 4 μM FCCP were added. Each trace represents the tetramethylrhodamine methyl ester (TMRM) fluorescence of a single fiber. The fraction of myofibers with depolarizing mitochondria is indicated for each condition.

(I) Densitometric quantification of the carbonylated proteins extracted from WT and *TFEB* KO mice. Values are mean ± SEM (n = 4; *p < 0.05). A representative immunoblot for carbonylated proteins is shown in Figure S3C.

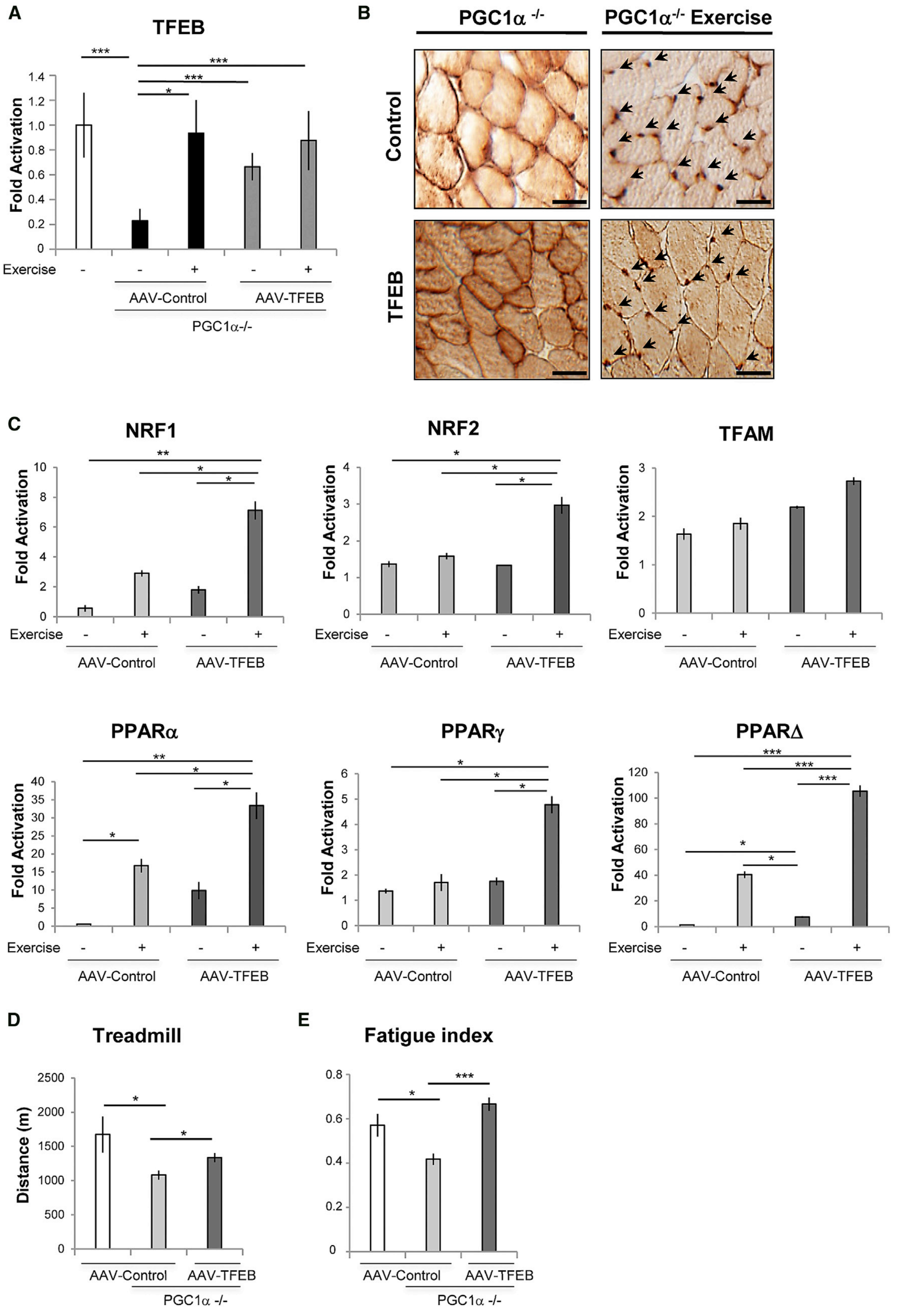

(legend on next page)

3B). Importantly, exercise restored a normal TFEB expression, induced TFEB nuclear translocation, and triggered upregulation of genes related to mitochondrial biogenesis (Figures 3C and S4A).

EM analysis of overexpressed *TFEB* in *PGC1α* KO mice revealed increased mitochondrial volume and density (Figure S4B). *TFEB* overexpression in *PGC1α* KO muscle also resulted in increased COX and SDH activity along with increased activities of CI, II, III, and IV (Figures S4C and S4D). The levels of PGC1α targets such as TFAM, NRF1, and NRF2 were also significantly upregulated by *TFEB* overexpression, even in the absence of PGC1α (Figure S5A). Importantly, systemic delivery of TFEB in muscle-specific *PGC1α* KO mice improved their exercise tolerance (Figure 3D). Indeed, *TFEB* expression was able to restore normal fatigue index when expressed in *PGC1α* KO muscle (Figure 3E). Altogether, these findings suggest that the induction of mitochondrial biogenesis in *TFEB*-overexpressing muscles does not depend on the presence of PGC1α.

To determine whether PGC1β may compensate for the lack of PGC1α, we measured the effect of *TFEB* overexpression on mitochondrial biogenesis in cells that were silenced for PGC1α and PGC1β. Importantly, inhibition of both PGC1 factors did not prevent or reduce TFEB-mediated induction of genes related to mitochondrial biogenesis and mitochondrial respiratory chain activity (Figure S5B).

TFEB Controls Energy Balance in Skeletal Muscle during Exercise

Physical activity has a major impact on glucose homeostasis and mitochondrial biogenesis and function. Therefore, we checked whether acute exhausting versus mild and chronic exercise regiments are equally able to activate TFEB. As a readout of TFEB activation, we monitored its nuclear localization. While acute exhausting contraction led to nuclear translocation of TFEB (Figure 4A), mild exercise did not (Figure 4B). However, 7 weeks of training with progressive increase of intensity without reaching exhaustion induced a massive TFEB nuclear translocation with concomitant cytosolic depletion (Figure 4B). Therefore, intensity and duration of training are critical factors that affect TFEB nuclear translocation and transcriptional regulation of genes related to mitochondrial biogenesis and function (Figure 4C).

To determine the physiological consequences of TFEB translocation during physical activity, we examined exercise performance in both *TFEB* KO and inducible muscle-specific TFEB transgenic mice. High-intensity exercise revealed significant training intolerance of *TFEB* KO mice compared to controls (Figure 5A). Conversely, acute muscle-specific activation of TFEB enhanced physical performance (Figure 5A). To better understand the exercise intolerance of the *TFEB* KO mice, we examined energy expenditure during treadmill running. While WT mice maintained constant levels of energy expenditure during physical activity, the *TFEB* KO mice displayed a drop after 15 min of physical exercise (Figure 5B). Metabolic analyses revealed that in basal condition, *TFEB* KO mice have a higher respiratory exchange rate (RER) than controls (Figure 5C). These data suggest that *TFEB* KO mice depend on glucose oxidation more than controls. In addition, while WT mice maintained a relatively constant RER during running period, *TFEB* KO mice showed a drop in RER after 20 min (Figure 5C). This decrease indicates a shift in substrate usage from glucose to fat metabolism. Finally, we measured glucose and fatty acid levels in muscle and blood from *TFEB* KO and TFEB transgenic mice before and after exercise. *TFEB* KO mice showed lower blood glucose levels compared to WT mice in basal condition (Figure 5D). Exercise caused a 50% reduction of blood glucose in *TFEB* KO, TFEB transgenic, and control mice (Figure 5D). Insulin levels mirrored the changes of blood glucose, as they were reduced in basal condition in *TFEB* KO mice and dropped after exercise in the different genotypes (Figure 5E).

TFEB KO mice are hypoglycemic, contain dysfunctional mitochondria, and produce less ATP. Thus, we reasoned that they use anaerobic glycolysis to produce energy. Consistent with this hypothesis, we found higher levels of lactate in the blood of *TFEB* KO mice before and after exercise compared to controls (Figure 5F). Conversely, lactate of transgenic mice was already lower than controls in resting condition and did not increase after exercise (Figure 5F). Therefore, TFEB transgenic mice better utilize glucose for energy production. To further confirm this finding, we measured glycogen levels in muscle. Glycogen levels were remarkably lower in *TFEB* KO mice and higher in TFEB transgenic in basal condition compared to WT. Enzymatic quantification showed that glycogen content was 3-fold less after TFEB ablation (Figure 5G), while it was 10-fold higher after TFEB overexpression compared to controls (Figure 5G). This was confirmed by periodic acid-Schiff (PAS) staining (Figure 5H). Exercise led to glycogen consumption in both *TFEB* KO muscles and controls (Figure 5G). The lower glycogen content detected in sedentary *TFEB* KO muscles explains the decrease of RER

Figure 3. Exercise Induces TFEB Expression and Nuclear Localization in $PGC1\alpha^{-/-}$ Muscle

(A) TFEB mRNA levels of $PGC1\alpha^{-/-}$ muscles infected with AAV2.9 control virus (light gray) or AAV2.9-*TFEB* (dark gray). Data were compared with TFEB mRNA level of WT mice (white). The levels of TFEB were measured in sedentary condition and post-exercise as indicated. Error bars represent mean ± SE for n = 3; *p < 0.05, ***p < 0.001.

(B) TFEB immunohistochemical analysis of $PGC1\alpha^{-/-}$ muscles from sedentary and exercised mice. GCN muscle cryosections were immunostained by using anti-TFEB antibody. Control means endogenous TFEB in muscles infected with AAV2.9 control virus. TFEB means the transgene TFEB in muscles infected with AAV2.9-*TFEB*. Arrows indicate exercise-induced TFEB nuclear localization. The scale bars represent 50 μm.

(C) Analysis of genes related to mitochondrial biogenesis in $PGC1\alpha^{-/-}$ skeletal muscle infected with AAV2.9 control or with AAV2.9-*TFEB* virus. The mRNA levels were measured before and after acute exercise, as indicated. Data are shown as mean ± SE, n = 3; *p < 0.05, **p < 0.01, ***p < 0.001.

(D and E) Muscle fatigue and physical performance are improved by TFEB expression in PGC1α-deficient muscle.

(D) The mice were systemically injected with AAV2.9-*TFEB* or AAV2.9 control virus. After 3 weeks from the virus injection, mice were subjected to run until exhaustion, as described in the Experimental Procedures. Data are shown as mean ± SE, n = 6; *p < 0.05.

(E) Soleus muscles were transected by intramuscular injection of AAV2.1-*TFEB* or AAV2.1 control virus, which resulted in 100% transfection efficacy. Index for fatigue means tetanic muscle force generated after 100 s of stimulation divided by the maximal tetanic tension produced during the fatigue protocol. Data are shown as mean ± SE, n = 4; *p < 0.05, ***p < 0.001.

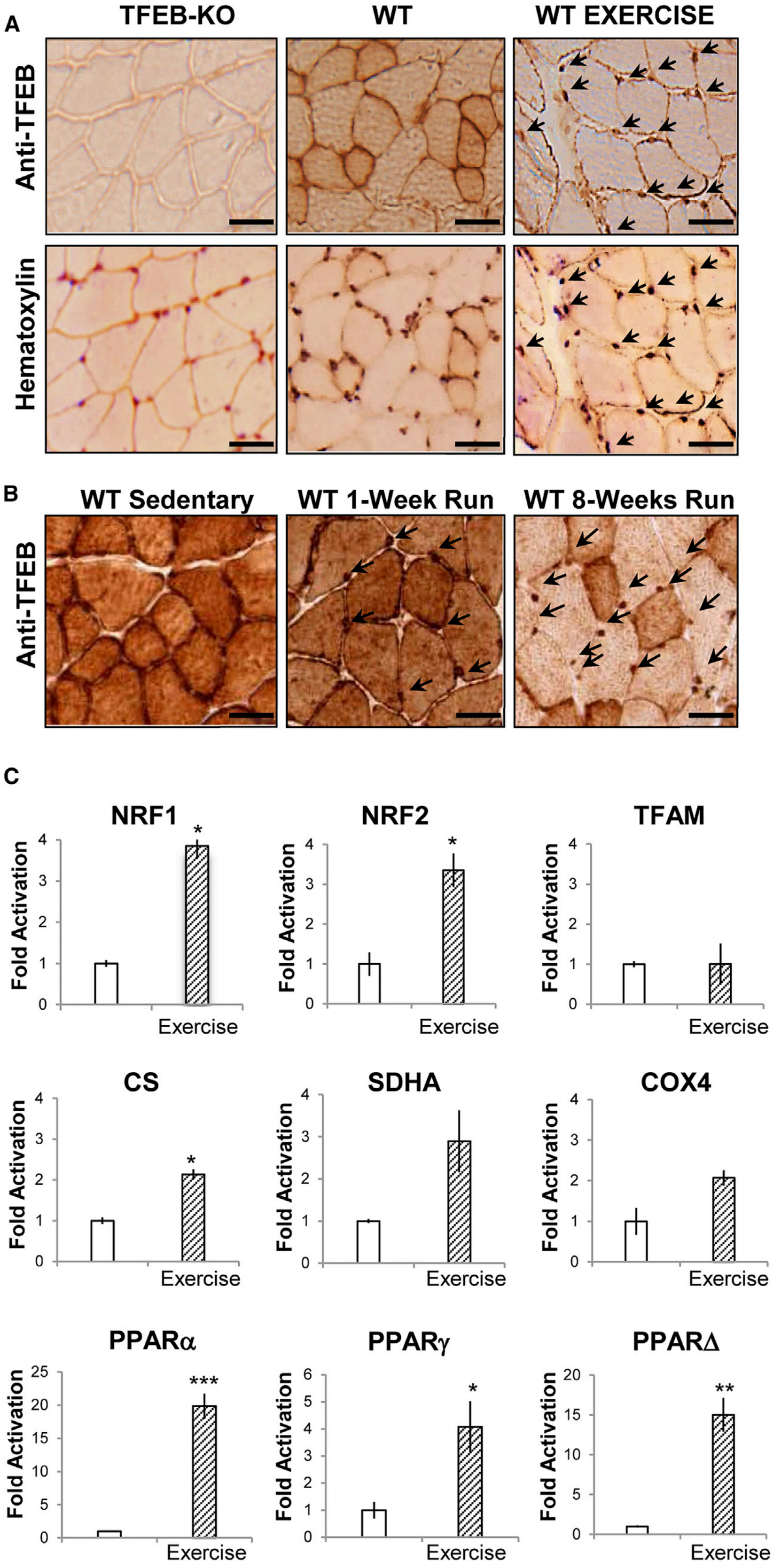

Figure 4. Exercise Induces TFEB Nuclear Translocation

(A) GCN tissue sections of *TFEB* KO and WT mice were immunostained with anti-TFEB antibody and counterstained with hematotoxylin as indicated. Arrows indicate nuclear localization of endogenous TFEB in WT mice after an acute bout of exercise. The scale bars represent 50 μm.

(B) Endogenous TFEB was immunostained in muscle cryosections of sedentary, 4 days mild exercised, and 7 weeks intense trained WT mice. Arrows indicate TFEB nuclear localization.

(C) Expression analysis of genes related to mitochondrial biogenesis and mitochondrial respiratory chain in WT skeletal muscle before and after acute exercise. Data are shown as mean ± SE, n = 3; *$p < 0.05$, **$p < 0.01$.

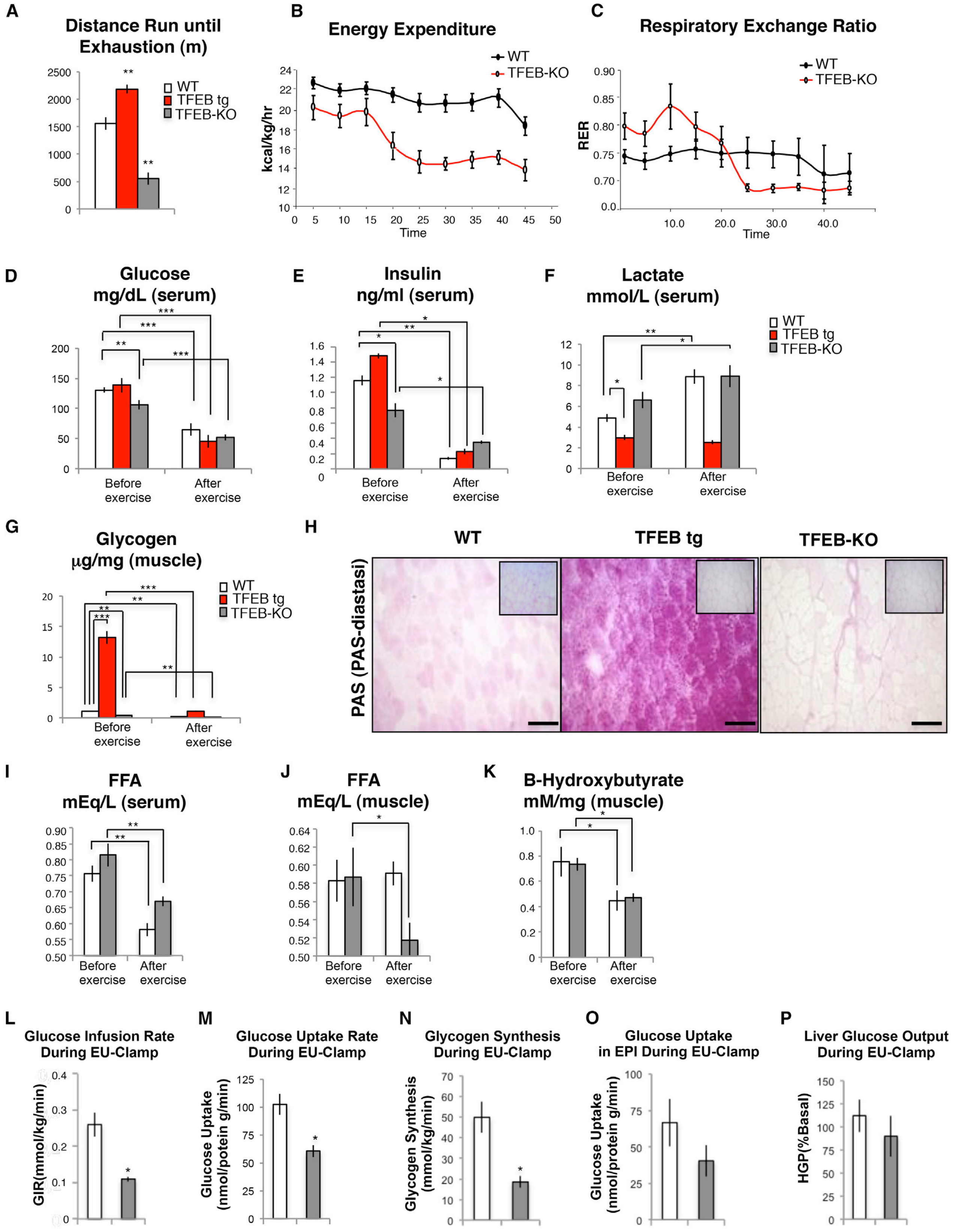

(legend on next page)

observed after a 15 min exercise (Figure 5C). Since glycogen is rapidly depleted in *TFEB* KO mice, the additional need for energy during exhausting exercise requires a switch from glycolysis to fatty acid oxidation. Consistently, while blood free fatty acid concentrations were reduced after exercise in both *TFEB* KO mice and controls (Figure 5I) and blood levels of non-esterified fatty acids (NEFAs) did not differ between genotypes (Figure 5I), their muscle content dramatically decreased after exercise only in *TFEB* KO mice (Figure 5J). Importantly, ketones did not differ between *TFEB* KO and controls (Figure 5K). These findings confirm a change in metabolic flexibility in the absence of TFEB that forced muscle cells to use lipids for ATP production. The exhaustion of the lipid fuel in KO mice results in inability to maintain the same exercise intensity of controls.

TFEB Controls Metabolic Flexibility and Energy Balance Independently of Autophagy

We and others have found that autophagy is important for mitochondrial quality control and is activated by exercise to clear dysfunctional mitochondria (Lo Verso et al., 2014). Thus, we checked whether TFEB controls autophagy in adult skeletal muscles. Surprisingly, TFEB activation was not sufficient to enhance autophagy flux and TFEB deletion did not impair autophagy flux in the presence or absence of nutrients (Figures S6A and S6B). Moreover, activation of TFEB did not induce protein breakdown and muscle loss. In fact, most of the atrophy-related genes belonging to the ubiquitin proteasome and autophagy-lysosome systems were not induced by TFEB expression (Figure S6C). Similarly, mitophagy genes were not upregulated. Since protein degradation is not affected by TFEB activation, we checked whether genes related to protein synthesis were modulated by TFEB. However, when we checked a cross-sectional area, we found a shift toward smaller size in transgenic mice, suggesting that protein synthesis was not induced. This decrease in fiber size is due to a metabolic shift because oxidative fibers are smaller than glycolytic skeletal muscle fibers (Figure S7A). Furthermore, we did not find any significant difference in myosin distribution between transgenic and control muscles (Figure S7B). Therefore, TFEB controls myofiber metabolism, but not myosin content/type, independently of autophagy or proteostasis.

TFEB Controls Glucose Homeostasis and Insulin Sensitivity Independently of PGC1α

Because muscles from *TFEB* KO mice contain lower glycogen levels than controls, we reasoned that they may have abnormal regulation of glucose homeostasis. Thus, we performed euglycemic-hyperinsulinemic (EU) clamps and observed that the glucose infusion rate (GIR) that is required to maintain a constant glycemia during insulin treatment was significantly reduced in *TFEB* KO compared to control mice (Figure 5L). The reduction of GIR was consequent to a decrease in skeletal muscle glucose uptake (Figure 5M), which caused a decrease of glycogen synthesis (Figure 5N). Although there was an apparent small decrease in adipose tissue glucose uptake, this was not statistically significant (Figure 5O). Moreover, insulin was equally effective in suppressing hepatic glucose output during the clamp experiment (Figure 5P). Together, these data demonstrate that TFEB deficiency in skeletal muscle results in peripheral insulin resistance-reduced glucose uptake and decreased glycogen content.

These findings are consistent with the transcriptomic signature of *TFEB*-overexpressing and *TFEB* KO muscles. To further confirm these findings, we monitored the expression levels of glucose homeostasis-related genes in muscles injected with AAV-*TFEB* and in muscles of TFEB transgenic mice and found a significant increase in the expression of GLUT1 and GLUT4, the GTPase involved in GLUT4 translocation (TBC1D1), and

Figure 5. TFEB Controls Energy Balance in Skeletal Muscle

(A) High-intensity exhaustive exercise. To determine exercise capacity, mice were run on a treadmill. During high-intensity exercise, transgenic mice (red) ran more, while *TFEB* KO mice (gray) ran half as much as WT mice (white). Data are shown as mean ± SE, n = 10; **p < 0.01.

(B and C) Energy expenditure (B) and RER (C) were determined during exercise in *TFEB* KO and WT mice. The mice ran at a fixed speed of 10 m/min and an incline of 20°. The figure shows the mean RER measured at peak oxygen consumption. Data were transformed by Blom's method to obtain both normally distributed data and normally distributed residual. Two-way ANOVA was used for comparison of RERs in the two groups during the entire resting period, whereas t test was used for comparison of individual time points, n = 8.

(D–F) Blood and muscle metabolites before and after high-intensity exercise in WT (white), TFEB transgenic (red), and *TFEB* KO mice (gray). Data are shown as mean ± SE, n = 8; *p < 0.05, **p < 0.01, ***p < 0.001.

(G) Enzymatic quantification of muscle glycogen before and after high-intensity exercise in WT (white), TFEB transgenic (red), and *TFEB* KO mice (gray). Data are shown as mean ± SE, n = 8; **p < 0.01.

(H) Periodic acid-Schiff (PAS) staining of cryosections from AAV2.1-*GFP*, AAV2.1-*TFEB* transfected, and *TFEB* KO mice. Inserts show PAS staining after glycogen breakdown. The scale bars represent 100 μm.

(I and J) Quantitative analysis of serum (I) and muscle (J) NEFA before and after high-intensity exercise in *TFEB* KO (gray) and WT mice (white). Data are shown as mean ± SE, n = 8; *p < 0.05, **p < 0.01.

(K) Enzymatic quantification of B-hydroxybutyrate before and after high-intensity exercise in *TFEB* KO (gray box) and WT mice (white box). Data are shown as mean ± SE, n = 8; *p < 0.05.

(L) TFEB ablation results in a decreased rate of insulin-stimulated GIR. EU clamps were used to assess whole-body insulin sensitivity by determining the GIR required to maintain euglycemia in WT (white) and *TFEB* KO (gray) mice. Data represent the means ± SE from five to six individual mice per group; *p < 0.05.

(M) TFEB deletion results in a reduction of insulin-stimulated glucose uptake in muscle. Insulin-stimulated glucose uptake into muscle tissues was determined by 2-deoxy-d-[1-^{14}C]glucose injection during the last 35 min of insulin infusion during the EU clamp. These data represent the means ± SE of five to six mice per group; *p < 0.05. WT, white; *TFEB* KO, gray.

(N) The amount of glucose conversion to glycogen in GCN muscles from WT (white) and *TFEB* KO (gray) mice was determined during the EU clamp by infusion of [3-^{3}H]glucose. Data represent the means ± SE from five to six mice per group; *p < 0.05.

(O and P) *TFEB* KO mice do not show any significant difference in glucose homeostasis of liver or adipose tissue. (O) Glucose uptake into adipose tissue (EPI) and (P) insulin suppression of hepatic glucose output (HGP, liver) were determined during the EU clamp by infusion of 2-deoxy-d-[1-^{14}C]glucose or [3-^{3}H]glucose infusion into WT mice (white) and *TFEB* KO (gray) mice. These data represent the means ± SE from five to six mice per group.

the rate-limiting enzymes of glycolysis (hexokinase 1 and 2) (Figure 6A). Immunoblotting analyses confirmed increased protein levels of GLUT1 in *TFEB*-overexpressing muscles (Figure 6B). The induction of genes involved in glucose uptake was also coupled with an increase of transcript and protein of GYS (Figures 6C and 6D).

GYS activity is negatively regulated by phosphorylation of the C-terminal region by GYS kinases (GSK3s) (Jensen and Lai, 2009). However, the phospho-GYS levels were unchanged in AAV-*TFEB* muscles, and therefore, the pGYS/GYS ratio was dramatically decreased in *TFEB*-overexpressing muscles when compared to controls (Figure 6D). Furthermore, expression analysis of genes related to glucose metabolism did not reveal any significant difference between *PGC1α* KO and controls after *TFEB* overexpression (Figure 6E). Finally, EM showed an accumulation of glycogen in *PGC1α* KO mice that were infected by AAV2.1-*TFEB* (Figure S4B). Quantitative and qualitative analyses of glycogen showed that TFEB induced a higher glycogen accumulation in *PGC1α* KO muscle than in WT (Figures 6F and 6G). These data indicate that TFEB is able to control muscle glycogen content in a PGC1α-independent manner.

Glucose-Related Signaling Pathways Are Affected by TFEB

The activity of GLUT transporters is tightly controlled by several pathways. Thus, we checked whether TFEB impinges not only on GLUT1/4 expression but also on glucose-related signaling. Previous studies have shown that nitric oxide (NO) controls several metabolic aspects of skeletal muscle, including mitochondrial biogenesis and glucose uptake. NO is formed by nitric oxide synthase (NOS) via the conversion of L-arginine to L-citrulline. Skeletal muscles express neuronal (nNOS), endothelial (eNOS), and inducibile (iNOS) isoforms (Andrew and Mayer, 1999). Moreover, nNOS is the major isoform involved in AMPK-dependent regulation of GLUT4 (Lira et al., 2007). We found that the nNOS transcription and protein expression were increased in *TFEB*-overexpressing muscle. Conversely, real-time PCR and immunoblotting experiments showed a decrease of nNOS expression in *TFEB* KO muscle, as compared with controls (Figures 7A and 7B). To test whether nNOS and glucose transporter genes are direct targets of TFEB, we analyzed their promoters and identified CLEAR sites. ChIP experiments showed that TFEB is recruited on nNOS, GLUT1, and GLUT4 promoters (Figure 7C). Since AMPK is a downstream target of nNOS (Lira et al., 2010), we monitored the activation of AMPK. *TFEB* overexpression in muscle showed a significant induction of AMPK phosphorylation and of its downstream target acetyl-CoA carboxylase (ACC). Interestingly, *TFEB* overexpression triggered protein kinase B (AKT) activation. However, no changes in pAMPK and pACC were found in *TFEB* KO muscles (Figure 7D). Consistent with the presence of insulin resistance, we detected decreased AKT phosphorylation in *TFEB* KO muscles.

DISCUSSION

The beneficial effects of physical activity on mitochondrial content/function, fatty acid oxidation, and glucose homeostasis are well known (Hawley, 2002; Holloszy and Coyle, 1984; Holloszy et al., 1998). Indeed, muscle activity is important to counteract disease progression in diabetes, obesity, and metabolic syndrome. Here we have found that TFEB is a major regulator of glucose homeostasis and mitochondrial biogenesis to provide the energetic support to maintain muscle contraction. Our in vivo data show that the absence of TFEB causes accumulation of morphologically abnormal and dysfunctional mitochondria that display impairment in respiratory chain complex II activity and proton leakage, leading to a defect in ATP production and exercise intolerance. Conversely, overexpression of TFEB induces mitochondrial biogenesis, improves respiratory chain complex activities, and increases ATP production. These findings are surprising since the documented effects of TFEB are mainly related to lysosomal biogenesis and autophagy regulation.

The positive effects of TFEB on the mitochondrial network in skeletal muscle appear to be independent from PGC1α. We found that TFEB is sufficient to induce the expression of NRF2 and Tfam, two major master regulators of mitochondrial biogenesis in muscle, even in the absence of both PGC1α and PGC1β, and is both sufficient and required for PPARα expression. Therefore, TFEB acts independently of PGC1α to promote oxidation of glucose and lipids, which are critical substrates during the early and late phase of strenuous contraction, respectively. Therefore, TFEB is at least one missing factor that explains why PGC1α is dispensable for exercise and mitochondrial biogenesis (Rowe et al., 2012). Moreover, TFEB directly controls glucose homeostasis via GLUT1/4 expression and insulin sensitivity via nNOS. Indeed, muscle from *TFEB* KO mice showed decreased glucose uptake during EU clamps, nearly completely absent glycogen stores, and reduced AKT phosphorylation under resting conditions. The insulin resistance of the TFEB-deficient fibers prevents glucose oxidation and therefore drives the exercising muscle to use fatty acid oxidation, which consequently blocked pyruvate dehydrogenase (PDH) enzyme (Figure 2A), resulting in lactate accumulation. Altogether, these findings suggest that TFEB is a critical player of metabolic flexibility during physical activity.

In a previous study, we reported that TFEB regulates lipid metabolism in liver, and this effect appears to be mediated, at least in part, by PGC1α (Settembre et al., 2013). Conversely, our findings in muscle show that TFEB is directly involved in mitochondrial function and glucose homeostasis independently from PGC1α. These observations indicate that the networks of genes regulated by TFEB are context specific, for the gene expression profiles have significant tissue-specific changes, supporting distinct tissue-specific metabolic functions.

Calcium signaling is greatly affected by exercise, and the calcium-dependent phosphatase calcineurin is one of the most important players for muscle adaptation to physical activity. We have recently found that exercise triggers TFEB nuclear localization in a calcineurin-dependent fashion (Medina et al., 2015). Calcineurin dephosphorylates TFEB serine residues that play a critical role in determining TFEB subcellular localization and promotes its nuclear translocation (Medina et al., 2015). Importantly, calcium-dependent signaling also modulates exercise-dependent, glucose-related pathways. In fact, muscle-specific transgenic mice that overexpress an activated form of calcineurin show increased glycogen accumulation and lipid oxidation (Long et al., 2007) and upregulation of PGC1α, several glycolytic enzymes, mitochondrial genes, genes related to lipid metabolism, and GLUT4 (Long and Zierath, 2008). Conversely,

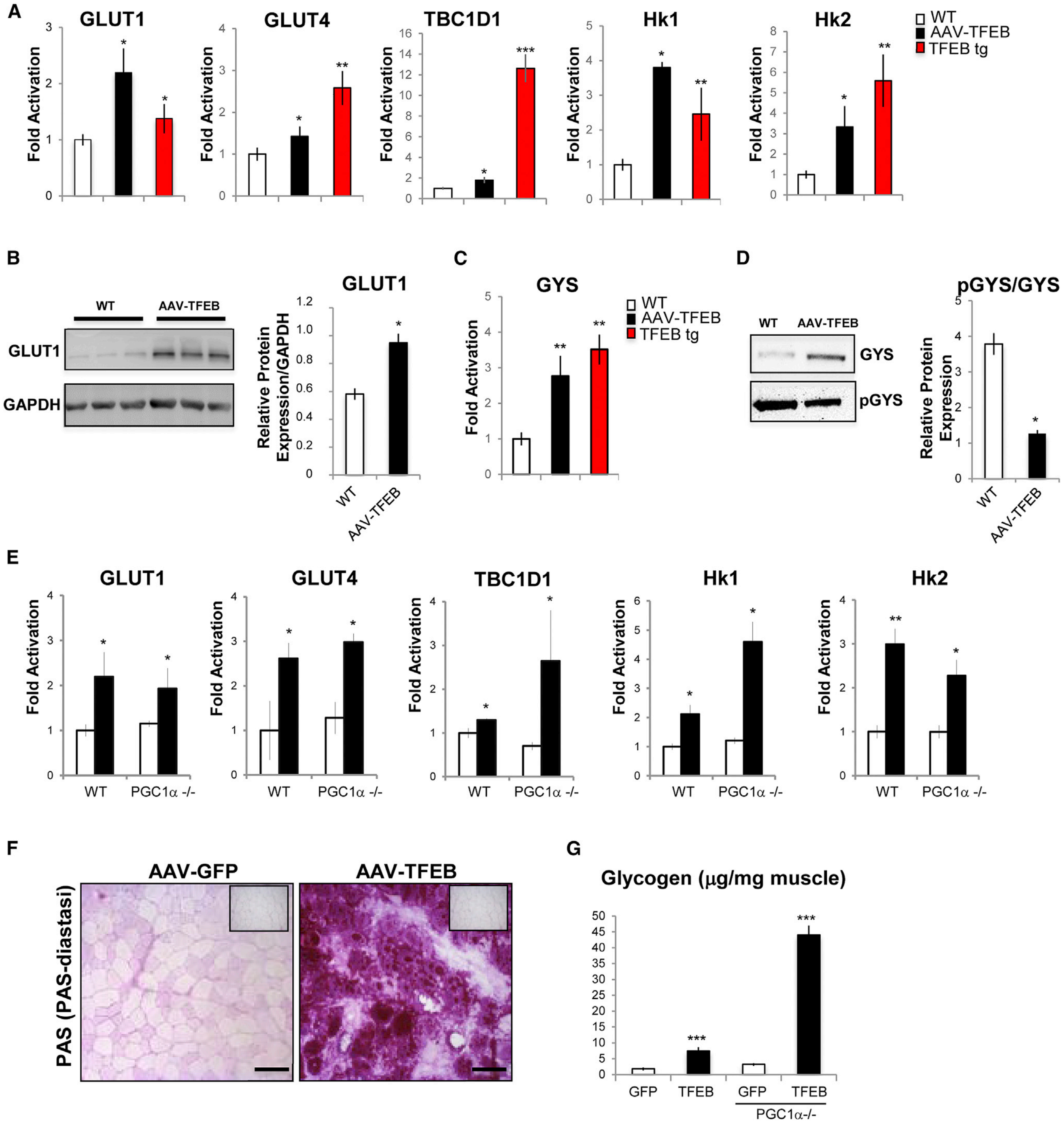

Figure 6. TFEB Controls Genes Related to Glucose Metabolism

(A and C) Quantitative real-time PCR of glucose uptake and glycogen biosynthesis-related genes in GCN muscles infected with AAV2.1-TFEB (black bar) and in TFEB transgenic muscles (red bar) compared with WT (white bar) muscles. Data were normalized for GAPDH and expressed as fold induction relative to the WT. Data are shown as mean ± SE, n = 3; *p < 0.05, **p < 0.01.

(B) Western blot of GLUT1 and densitometric quantification. Values are normalized for GAPDH; *p < 0.05.

(D) Western blot analysis of total and phosphorylated GYS from extracts of GCN. Representative blot images are shown (left panel). Densitometric quantification is depicted on the right panel. Data are shown as mean ± SE, n = 3; *p < 0.05.

(E–G) TFEB regulates glycogen synthesis independently of PGC1α.

(E) Quantitative real-time PCR of genes related to glucose uptake and glycogen biosynthesis in *PGC1α*$^{-/-}$ and WT mice that were infected with AAV2.1-*GFP* (white) or AAV2.1-*TFEB* (black). Data are shown as mean ± SE, n = 3; *p < 0.05, **p < 0.01.

(F) PAS staining of cryosections from *PGC1α*$^{-/-}$ muscles that were transfected by AAV2.1-*GFP* or AAV2.1-*TFEB*. The scale bars represent 100 μm.

(G) Enzymatic quantification of basal glycogen levels in *PGC1α*$^{-/-}$ and WT muscle, infected with AAV2.1-*GFP* (white) or AAV2.1-*TFEB* (black).

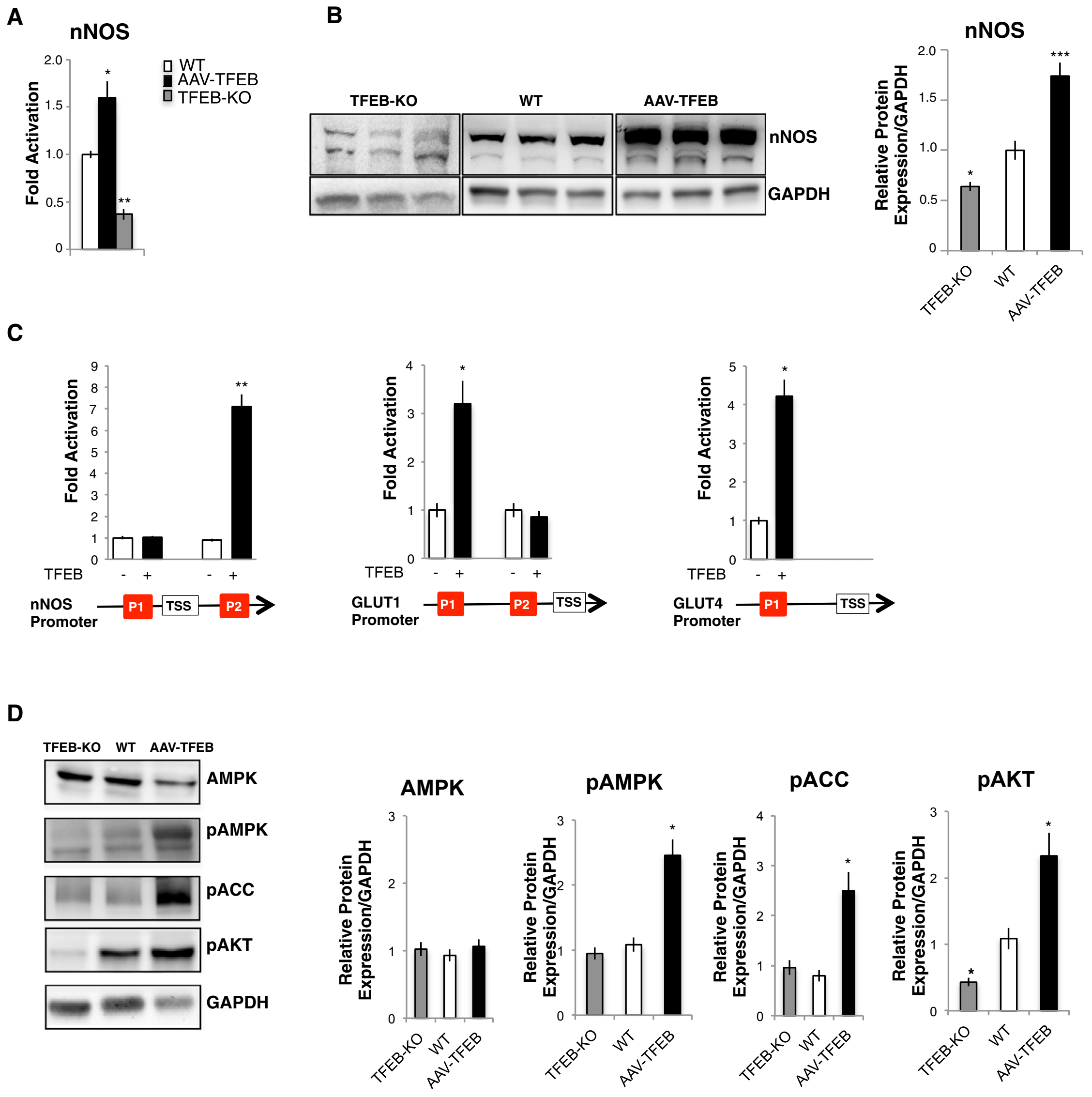

Figure 7. TFEB Also Controls Glucose Uptake via Regulation of AMPK Signaling Pathways

(A) Quantitative real-time PCR of nNOS transcript in WT (white bar), *TFEB*-overexpressing (black), and *TFEB* KO muscles (gray). Values are expressed as fold induction compared to controls. Data are shown as mean ± SE, n = 8; *p < 0.05, **p < 0.01.

(B) Western blot of nNOS and densitometric quantification. Value are normalized for GAPDH; *p < 0.05; ***p < 0.001.

(C) ChIP analyses of muscles from TFEB transgenic and WT mice on nNOS, GLUT1, and GLUT4 promoters. The histograms show the amount of immunoprecipitated DNA as detected by quantitative real-time PCR assay. Data represent mean ± SE of three independent experiments; *p < 0.05.

(D) Western blot of AMPK, PAMPK, PACC, and PAKT performed on protein extracts from GCN muscles of AAV2.1-*TFEB* transfected, *TFEB* KO, or WT mice. Representative images (left) and densitometric quantification (right) are shown; *p < 0.05.

muscle-specific calcineurin KO mice show exercise intolerance when subjected to exhausting physical activity (Pfluger et al., 2015). Finally, immunosuppression therapy by high-dose treatment with calcineurin inhibitors cylosporin A or tacrolimus (FK-506) has been associated with a higher risk for developing obesity and diabetes in patients (T D Correia et al., 2003). Altogether, these data suggest that calcineurin plays a major role in glucose and lipid metabolism, although the mechanistic insights of these beneficial effects of calcineurin are unknown. Our data suggest that TFEB is a critical calcineurin downstream target that coordinates metabolic adaptations such as glucose uptake and mitochondria function to optimize energy production to sustain muscle contraction.

TFEB not only regulates expression of glucose transporters and critical glycolytic enzymes but also factors that impinge on AMPK regulation, such as nNOS. It was shown that nNOS controls GLUT4 expression in skeletal muscle cells through AMPK activation. In addition, endogenous nNOS is required for the upregulation of AMPK activity by the AMP mimetic, AICAR (Higaki et al., 2001; Lira et al., 2010; McConell et al., 2010).

In summary, our results position TFEB as a central coordinator of insulin sensitivity, glucose homeostasis, lipid oxidation, and

mitochondrial function, further emphasizing the importance of this regulatory pathway in the metabolic response to energy-demanding conditions such as physical exercise.

EXPERIMENTAL PROCEDURES

Cell Culture, Plasmids, and Transfection Reagent

Detailed information in the Supplemental Experimental Procedures.

Real-Time PCR

Quantitative real-time PCR analyses were performed on a LigthCycler 480 II (Roche). For detailed information on preparation and gene expression analysis, see the Supplemental Experimental Procedures.

Generation of Muscle-Specific TFEB KO and Inducible Muscle-Specific Transgenic Mice and In Vivo Experimental Procedures

Detailed information in the Supplemental Experimental Procedures.

Western Blot Analysis and Antibodies

Total homogenates were prepared in RIPA buffer (50 mM Tris HCl [pH 8], 150 mM NaCl, 1% NP-40, 0.5% sodium deoxycholate, and 0.1% SDS) with the addition of a protease inhibitor cocktail (Roche). Protein concentration was determined by the Lowry method. Aliquots, 50 μg each, were run through an SDS-PAGE and electroblotted onto a PVDF membrane, which was then matched with different antibodies. For the antibodies used in western blot analysis, see the Supplemental Experimental Procedures.

Biochemical Analysis of Mitochondrial Respiratory Chain Complex

Muscle samples stored in liquid nitrogen were homogenized in 10 mM phosphate buffer (pH 7.4), and the spectrophotometric activity of cI, cII, cIII, and cIV, as well as citrate synthase (CS), was measured as described (Bugiani et al., 2004). Detailed information in the Supplemental Experimental Procedures.

Morphological Analysis

Histochemical and ultrastructural analyses were performed as described (Sciacco and Bonilla, 1996). Detailed information in the Supplemental Experimental Procedures.

Isolation of Skeletal Myofibers and Measurements of Mitochondrial Membrane Potential

Muscle fibers were isolated from FDB muscle and mitochondrial membrane potential measured by epifluorescence microscopy on the basis of the accumulation of TMRM fluorescence, as previously described (Lo Verso et al., 2014). Fibers were considered as depolarizing when they lost more than 10% of the initial value of TMRM fluorescence. Imaging was performed with a Zeiss Axiovert 100 TV inverted microscope equipped with a 12-bit digital cooled charge-coupled device camera (Micromax, Princeton Instruments). The results were analyzed with MetaFluor imaging software (Universal Imaging).

Acute Exercise, Training, and Fatigue Experiments

For acute and chronic exercise studies, 16-week-old mice performed concentric exercise on a treadmill (Biological Instruments, LE 8710 Panlab Technology 2B) with a 10° incline, according to the protocol of exercise previously described (Medina et al., 2015). Total running distance was recorded for each mouse. Detailed information in the Supplemental Experimental Procedures.

Immunohistochemistry

Detailed information in the Supplemental Experimental Procedures.

Electron Microscopy

Detailed information in the Supplemental Experimental Procedures.

Plasma Chemistry Analysis

Blood was collected from the orbital plexus under isoflurane (Vedco) anesthesia. Plasma was frozen in aliquots at −20°C or used immediately after collection. Specific enzymatic kits were used for determination of serum NEFAs (Wako) and lactate (Abcam). Plasma glucose was monitored by a glucometer. Insulin was measured by ELISA (Mercodia).

Tissue Metabolite Quantification

Detailed information in the Supplemental Experimental Procedures.

Whole-Body Indirect Calorimetry

Metabolic measurements were performed using an Oxymax indirect calorimetry system (Columbus Instruments) (Zong et al., 2011). Detailed information in the Supplemental Experimental Procedures.

In Vivo Assessment of Insulin Action and Glucose Metabolism

Four days before the experiment, the mice were anesthetized and an indwelling catheter was introduced into the left internal jugular vein. The mice were fully recovered from the surgery before the in vivo experiments, as reflected by their reaching preoperative weight. After an overnight fast, EU clamps were conducted in conscious mice as previously described (Ayala et al., 2006; Zong et al., 2012). Detailed information in the Supplemental Experimental Procedures.

ChIP Assays

We performed ChIP assays on adult skeletal muscle overexpressing *TFEB*3X-Flag, and on WT muscle as control, by using the ChIP assay kit (Upstate) according to Milan et al. (2015). For immunoprecipitation, we used anti-FLAG antibody (F7425, Sigma-Aldrich). Oligonucleotide primers for amplification of a TFEB binding site on the GLUT1, GLUT4, nNOS, NRF1, NRF2, and TFAM promoters are listed in Table S7.

Microarray Data Analysis

Detailed information in the Supplemental Experimental Procedures.

Statistical Analysis

Data are expressed as mean values ± SE. Results were evaluated by repeated-measures ANOVA, multivariate ANOVA (MANOVA), or Student's two-tailed t test. $p < 0.05$ was considered statistically significant. In all figures, *$p < 0.05$, **$p < 0.01$, and ***$p < 0.001$.

SUPPLEMENTAL INFORMATION

Supplemental Information includes Supplemental Experimental Procedures, seven figures, and seven tables and can be found with this article online at http://dx.doi.org/10.1016/j.cmet.2016.11.003.

AUTHOR CONTRIBUTIONS

G.M., M.S., and A.B. designed the project and experiments and wrote the manuscript. G.M., A.A., and C.V. performed experiments and analyzed and interpreted data. G.M. and A.A. generated transgenic mice. E.V.P., C.L., I.D.M., V.R., H.Z., B.B., F.S., and C.T. contributed to experiments and data collection; P.G. and P.B. provided reagents and discussed data; R.D.C. performed computational analysis; and L.D., S.M., and P.K.S. provided technical expertise. J.E.P., M.Z., M.S., and A.B. discussed, reviewed, and edited the manuscript. M.S. and A.B. supervised the work.

ACKNOWLEDGMENTS

We thank N. Brunetti, D. Medina, and C. Settembre for suggestions. We are grateful to E. Nusco and TIGEM Bioinformatic and HCS Facilities for technical support, in particular D. Carrella and S. Montefusco. We acknowledge support from the Italian Telethon Foundation to A.B. (TCR09003) and M.S. (TCP04009), the European Research Council (ERC) to A.B. (250154-CLEAR) and M.S. (282310-MyoPHAGY), the Italian Ministry of Education (MiUR) (PRIN 2010/2011) to M.S., CARIPARO and Foundation Leducq to M.S., the Pennsylvania University and Beyond Batten Disease Foundation to A.B., the Core Grant MRC-MBU and ERC FP7-322424 to M.Z., GR-2010-2306-756 and the Italian Ministry of Health to C.V., and the National Science Foundation of China (H0315: 81370531) to H.Z.

Received: January 19, 2016
Revised: June 24, 2016
Accepted: November 5, 2016
Published: December 20, 2016

REFERENCES

Andrew, P.J., and Mayer, B. (1999). Enzymatic function of nitric oxide synthases. Cardiovasc. Res. *43*, 521–531.

Ayala, J.E., Bracy, D.P., McGuinness, O.P., and Wasserman, D.H. (2006). Considerations in the design of hyperinsulinemic-euglycemic clamps in the conscious mouse. Diabetes *55*, 390–397.

Bothe, G.W., Haspel, J.A., Smith, C.L., Wiener, H.H., and Burden, S.J. (2000). Selective expression of Cre recombinase in skeletal muscle fibers. Genesis *26*, 165–166.

Bugiani, M., Invernizzi, F., Alberio, S., Briem, E., Lamantea, E., Carrara, F., Moroni, I., Farina, L., Spada, M., Donati, M.A., et al. (2004). Clinical and molecular findings in children with complex I deficiency. Biochim. Biophys. Acta *1659*, 136–147.

Calabria, E., Ciciliot, S., Moretti, I., Garcia, M., Picard, A., Dyar, K.A., Pallafacchina, G., Tothova, J., Schiaffino, S., and Murgia, M. (2009). NFAT isoforms control activity-dependent muscle fiber type specification. Proc. Natl. Acad. Sci. USA *106*, 13335–13340.

Gehlert, S., Bloch, W., and Suhr, F. (2015). Ca2+-dependent regulations and signaling in skeletal muscle: from electro-mechanical coupling to adaptation. Int. J. Mol. Sci. *16*, 1066–1095.

Grumati, P., and Bonaldo, P. (2012). Autophagy in skeletal muscle homeostasis and in muscular dystrophies. Cells *1*, 325–345.

Hawley, J.A. (2002). Adaptations of skeletal muscle to prolonged, intense endurance training. Clin. Exp. Pharmacol. Physiol. *29*, 218–222.

Higaki, Y., Hirshman, M.F., Fujii, N., and Goodyear, L.J. (2001). Nitric oxide increases glucose uptake through a mechanism that is distinct from the insulin and contraction pathways in rat skeletal muscle. Diabetes *50*, 241–247.

Holloszy, J.O., and Coyle, E.F. (1984). Adaptations of skeletal muscle to endurance exercise and their metabolic consequences. J. Appl. Physiol. *56*, 831–838.

Holloszy, J.O., Kohrt, W.M., and Hansen, P.A. (1998). The regulation of carbohydrate and fat metabolism during and after exercise. Front. Biosci. *3*, D1011–D1027.

Jensen, J., and Lai, Y.C. (2009). Regulation of muscle glycogen synthase phosphorylation and kinetic properties by insulin, exercise, adrenaline and role in insulin resistance. Arch. Physiol. Biochem. *115*, 13–21.

Kelly, D.P., and Scarpulla, R.C. (2004). Transcriptional regulatory circuits controlling mitochondrial biogenesis and function. Genes Dev. *18*, 357–368.

Lira, V.A., Soltow, Q.A., Long, J.H., Betters, J.L., Sellman, J.E., and Criswell, D.S. (2007). Nitric oxide increases GLUT4 expression and regulates AMPK signaling in skeletal muscle. Am. J. Physiol. Endocrinol. Metab. *293*, E1062–E1068.

Lira, V.A., Brown, D.L., Lira, A.K., Kavazis, A.N., Soltow, Q.A., Zeanah, E.H., and Criswell, D.S. (2010). Nitric oxide and AMPK cooperatively regulate PGC-1 in skeletal muscle cells. J. Physiol. *588*, 3551–3566.

Lo Verso, F., Carnio, S., Vainshtein, A., and Sandri, M. (2014). Autophagy is not required to sustain exercise and PRKAA1/AMPK activity but is important to prevent mitochondrial damage during physical activity. Autophagy *10*, 1883–1894.

Long, Y.C., and Zierath, J.R. (2008). Influence of AMP-activated protein kinase and calcineurin on metabolic networks in skeletal muscle. Am. J. Physiol. Endocrinol. Metab. *295*, E545–E552.

Long, Y.C., Glund, S., Garcia-Roves, P.M., and Zierath, J.R. (2007). Calcineurin regulates skeletal muscle metabolism via coordinated changes in gene expression. J. Biol. Chem. *282*, 1607–1614.

Mammucari, C., Milan, G., Romanello, V., Masiero, E., Rudolf, R., Del Piccolo, P., Burden, S.J., Di Lisi, R., Sandri, C., Zhao, J., et al. (2007). FoxO3 controls autophagy in skeletal muscle in vivo. Cell Metab. *6*, 458–471.

Martina, J.A., Chen, Y., Gucek, M., and Puertollano, R. (2012). MTORC1 functions as a transcriptional regulator of autophagy by preventing nuclear transport of TFEB. Autophagy *8*, 903–914.

Masiero, E., Agatea, L., Mammucari, C., Blaauw, B., Loro, E., Komatsu, M., Metzger, D., Reggiani, C., Schiaffino, S., and Sandri, M. (2009). Autophagy is required to maintain muscle mass. Cell Metab. *10*, 507–515.

McConell, G.K., Ng, G.P., Phillips, M., Ruan, Z., Macaulay, S.L., and Wadley, G.D. (2010). Central role of nitric oxide synthase in AICAR and caffeine-induced mitochondrial biogenesis in L6 myocytes. J. Appl. Physiol. *108*, 589–595.

McCullagh, K.J., Calabria, E., Pallafacchina, G., Ciciliot, S., Serrano, A.L., Argentini, C., Kalhovde, J.M., Lømo, T., and Schiaffino, S. (2004). NFAT is a nerve activity sensor in skeletal muscle and controls activity-dependent myosin switching. Proc. Natl. Acad. Sci. USA *101*, 10590–10595.

Medina, D.L., Di Paola, S., Peluso, I., Armani, A., De Stefani, D., Venditti, R., Montefusco, S., Scotto-Rosato, A., Prezioso, C., Forrester, A., et al. (2015). Lysosomal calcium signalling regulates autophagy through calcineurin and TFEB. Nat. Cell Biol. *17*, 288–299.

Milan, G., Romanello, V., Pescatore, F., Armani, A., Paik, J.H., Frasson, L., Seydel, A., Zhao, J., Abraham, R., Goldberg, A.L., et al. (2015). Regulation of autophagy and the ubiquitin-proteasome system by the FoxO transcriptional network during muscle atrophy. Nat. Commun. *6*, 6670.

Palmieri, M., Impey, S., Kang, H., di Ronza, A., Pelz, C., Sardiello, M., and Ballabio, A. (2011). Characterization of the CLEAR network reveals an integrated control of cellular clearance pathways. Hum. Mol. Genet. *20*, 3852–3866.

Pfluger, P.T., Kabra, D.G., Aichler, M., Schriever, S.C., Pfuhlmann, K., García, V.C., Lehti, M., Weber, J., Kutschke, M., Rozman, J., et al. (2015). Calcineurin links mitochondrial elongation with energy metabolism. Cell Metab. *22*, 838–850.

Puigserver, P., Wu, Z., Park, C.W., Graves, R., Wright, M., and Spiegelman, B.M. (1998). A cold-inducible coactivator of nuclear receptors linked to adaptive thermogenesis. Cell *92*, 829–839.

Roczniak-Ferguson, A., Petit, C.S., Froehlich, F., Qian, S., Ky, J., Angarola, B., Walther, T.C., and Ferguson, S.M. (2012). The transcription factor TFEB links mTORC1 signaling to transcriptional control of lysosome homeostasis. Sci. Signal. *5*, ra42.

Rowe, G.C., El-Khoury, R., Patten, I.S., Rustin, P., and Arany, Z. (2012). PGC-1α is dispensable for exercise-induced mitochondrial biogenesis in skeletal muscle. PLoS ONE *7*, e41817.

Sardiello, M., Palmieri, M., di Ronza, A., Medina, D.L., Valenza, M., Gennarino, V.A., Di Malta, C., Donaudy, F., Embrione, V., Polishchuk, R.S., et al. (2009). A gene network regulating lysosomal biogenesis and function. Science *325*, 473–477.

Sciacco, M., and Bonilla, E. (1996). Cytochemistry and immunocytochemistry of mitochondria in tissue sections. Methods Enzymol. *264*, 509–521.

Settembre, C., Di Malta, C., Polito, V.A., Garcia Arencibia, M., Vetrini, F., Erdin, S., Erdin, S.U., Huynh, T., Medina, D., Colella, P., et al. (2011). TFEB links autophagy to lysosomal biogenesis. Science *332*, 1429–1433.

Settembre, C., Zoncu, R., Medina, D.L., Vetrini, F., Erdin, S., Erdin, S., Huynh, T., Ferron, M., Karsenty, G., Vellard, M.C., et al. (2012). A lysosome-to-nucleus signalling mechanism senses and regulates the lysosome via mTOR and TFEB. EMBO J. *31*, 1095–1108.

Settembre, C., De Cegli, R., Mansueto, G., Saha, P.K., Vetrini, F., Visvikis, O., Huynh, T., Carissimo, A., Palmer, D., Klisch, T.J., et al. (2013). TFEB controls cellular lipid metabolism through a starvation-induced autoregulatory loop. Nat. Cell Biol. *15*, 647–658.

T D Correia, M.I., Rego, L.O., and Lima, A.S. (2003). Post-liver transplant obesity and diabetes. Curr. Opin. Clin. Nutr. Metab. Care *6*, 457–460.

Zong, H., Wang, C.C., Vaitheesvaran, B., Kurland, I.J., Hong, W., and Pessin, J.E. (2011). Enhanced energy expenditure, glucose utilization, and insulin sensitivity in VAMP8 null mice. Diabetes *60*, 30–38.

Zong, H., Armoni, M., Harel, C., Karnieli, E., and Pessin, J.E. (2012). Cytochrome P-450 CYP2E1 knockout mice are protected against high-fat diet-induced obesity and insulin resistance. Am. J. Physiol. Endocrinol. Metab. *302*, E532–E539.

Cell Metabolism, Volume *25*

Supplemental Information

Transcription Factor EB Controls

Metabolic Flexibility during Exercise

Gelsomina Mansueto, Andrea Armani, Carlo Viscomi, Luca D'Orsi, Rossella De Cegli, Elena V. Polishchuk, Costanza Lamperti, Ivano Di Meo, Vanina Romanello, Silvia Marchet, Pradip K. Saha, Haihong Zong, Bert Blaauw, Francesca Solagna, Caterina Tezze, Paolo Grumati, Paolo Bonaldo, Jeffrey E. Pessin, Massimo Zeviani, Marco Sandri, and Andrea Ballabio

Figure S1.

A

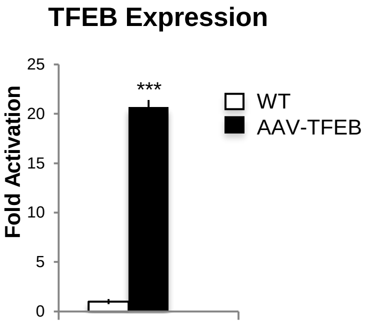
TFEB Expression
Fold Activation
25
20
15
10
5
0
***
WT
AAV-TFEB

B

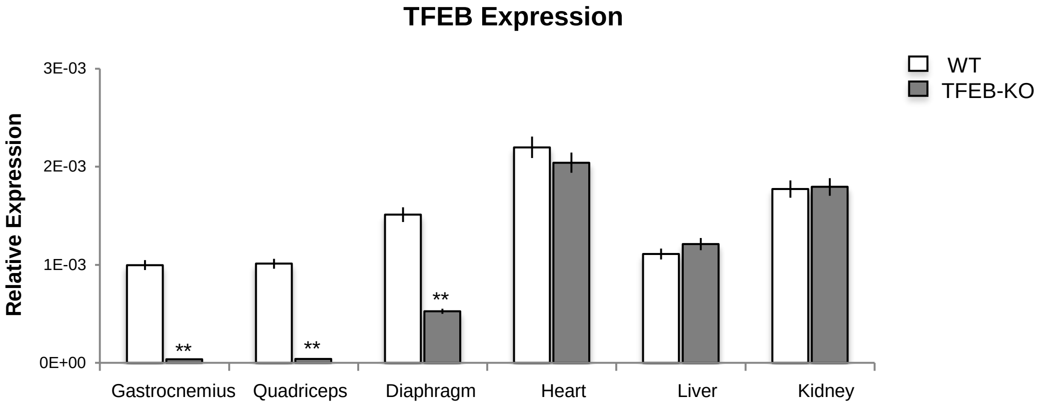
TFEB Expression
Relative Expression
3E-03
2E-03
1E-03
0E+00
**
**
**
Gastrocnemius
Quadriceps
Diaphragm
Heart
Liver
Kidney
WT
TFEB-KO

Figure S1. Expression analysis of TFEB transcript. Related to Figure 1. (A) The TFEB transcript from AAV2.1-*TFEB* transfected muscle was quantified by qRT-PCR (B) Expression levels of endogenous *Tcfeb* mRNA in muscles isolated from *TFEB*-KO and WT mice. Bar represent mean ± SE for $n = 5$; **p<0.01,***p<0.001s.

Figure S2.

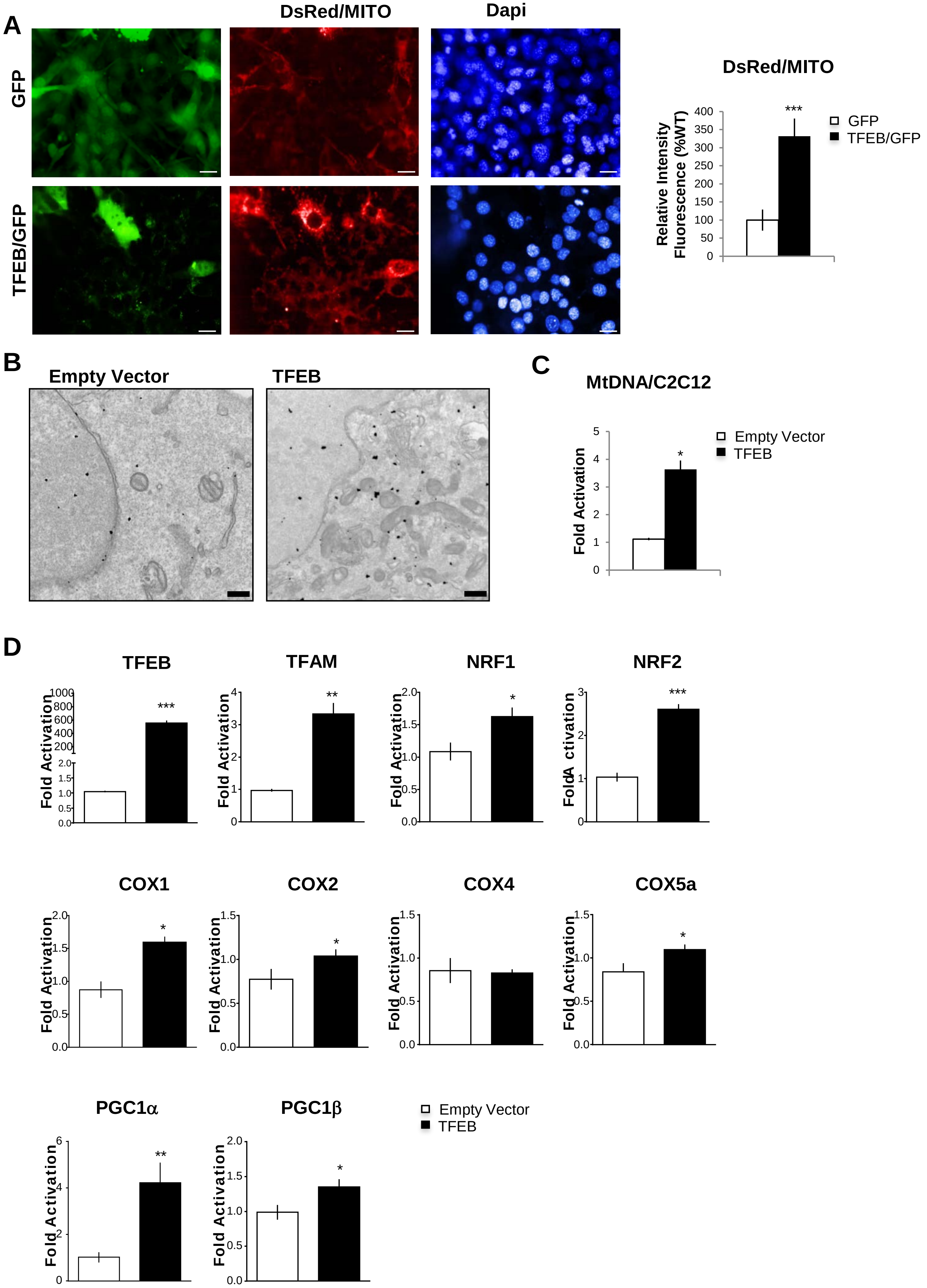
A
DsRed/MITO
Dapi
GFP
TFEB/GFP
DsRed/MITO
Relative Intensity Fluorescence (%WT)
***
GFP
TFEB/GFP
B
Empty Vector
TFEB
C
MtDNA/C2C12
Fold Activation
*
Empty Vector
TFEB
D
TFEB
TFAM
NRF1
NRF2
Fold Activation
***
**
*
***
COX1
COX2
COX4
COX5a
PGC1α
PGC1β
Empty Vector
TFEB

Figure S2. TFEB induces mitochondrial biogenesis in C2C12 cells. Related to Figure 1. (A) Confocal images of C2C12 cells co-transfected with DsRed/MITO and with *CMV-GFP* or *TFEB-GFP*. Nuclei were stained with DAPI (blue). Mitochondria are shown in red. DAPI and DsRed were excited with 405 (UV) and 561 laser lines, respectively. Images were acquired on a Perkin Elmer Opera automated confocal microscope. The scale bars represent 10 μm. Percentage of DsRed/MITO intensity was calculated using Prism software (see Materials and Methods for details) Mean ± SE, n = 3; **p<0.01. (B) Electron microscopy analysis of C2C12 cells that overexpress *TFEB*-3XFlag compared with control cells. The scale bars represent 500 nm. (C) Quantitative MtDNA analysis, by Rt-PCR, confirmed an increase of mtDNA when TFEB is overexpressed in muscle cells compared with WT condition. Error bars represent mean ± SE for n=3; **p<0.01. (D) Expression analysis of genes related to mitochondrial biogenesis and functions in C2C12 cells transfected by empty vector (white bar) or *TFEB*-3XFlag (black bar). Error bars represent mean ± SE for n=3; *p<0.05, **p<0.01, ***p<0.001.

Figure S3.

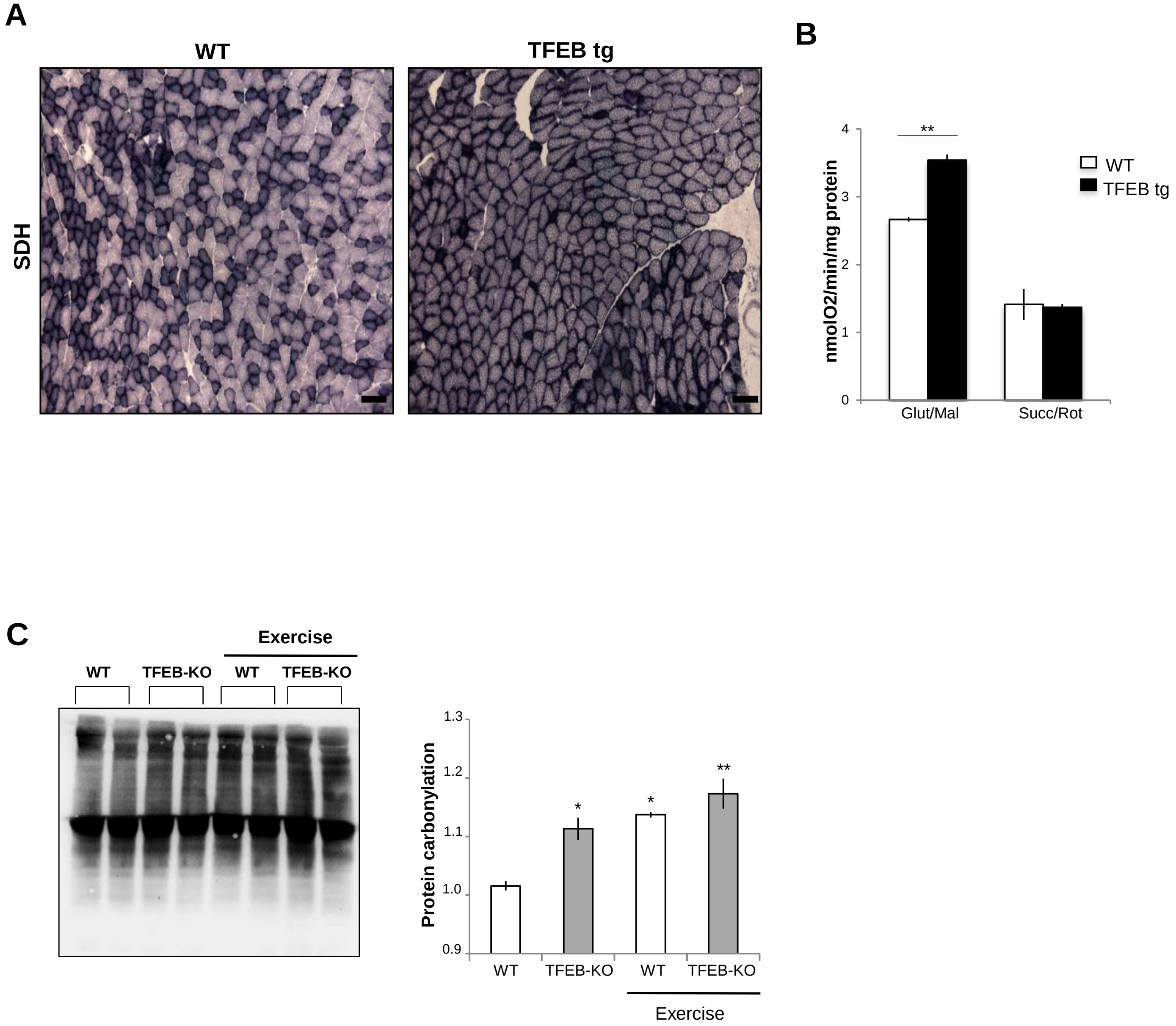
A
WT
TFEB tg
SDH
B
nmolO2/min/mg protein
4
3
2
1
0
**
WT
TFEB tg
Glut/Mal
Succ/Rot
C
Exercise
WT
TFEB-KO
WT
TFEB-KO
Protein carbonylation
1.3
1.2
1.1
1.0
0.9
*
*
**
WT
TFEB-KO
WT
TFEB-KO
Exercise

Figure S3. TFEB increases respiratory chain activity and glycogen synthesis in skeletal muscle. Related to Figure 2. Succinate dehydrogenase (SDH) staining of cryosections from TFEB transgenic mice compared with control mice. TFEB was induced for 2 weeks in 5 months old mice. The scale bars represent 100 nm. (B) Respiration of mitochondria from muscles of TFEB transgenic and control mice. TFEB significantly increased oxygen consumption rate when glutamate/malate were used as substrates. Data are presented as mean ± SE; n=3; **p < 0.01. (C) Overall protein carbonylation of WT and *TFEB*-KO muscles revealed by Oxyblot. A representative immunoblot for carbonylated proteins is depicted on the left; densitometric quantification of the carbonylated proteins is shown in the graph on the right. *TFEB*- KO mice show higher carbonylated than WT in both sedentary condition and after exercise. n =4; *p < 0.05). Values are mean ± SE for n = 4; *p < protein 0.05.

Figure S4.

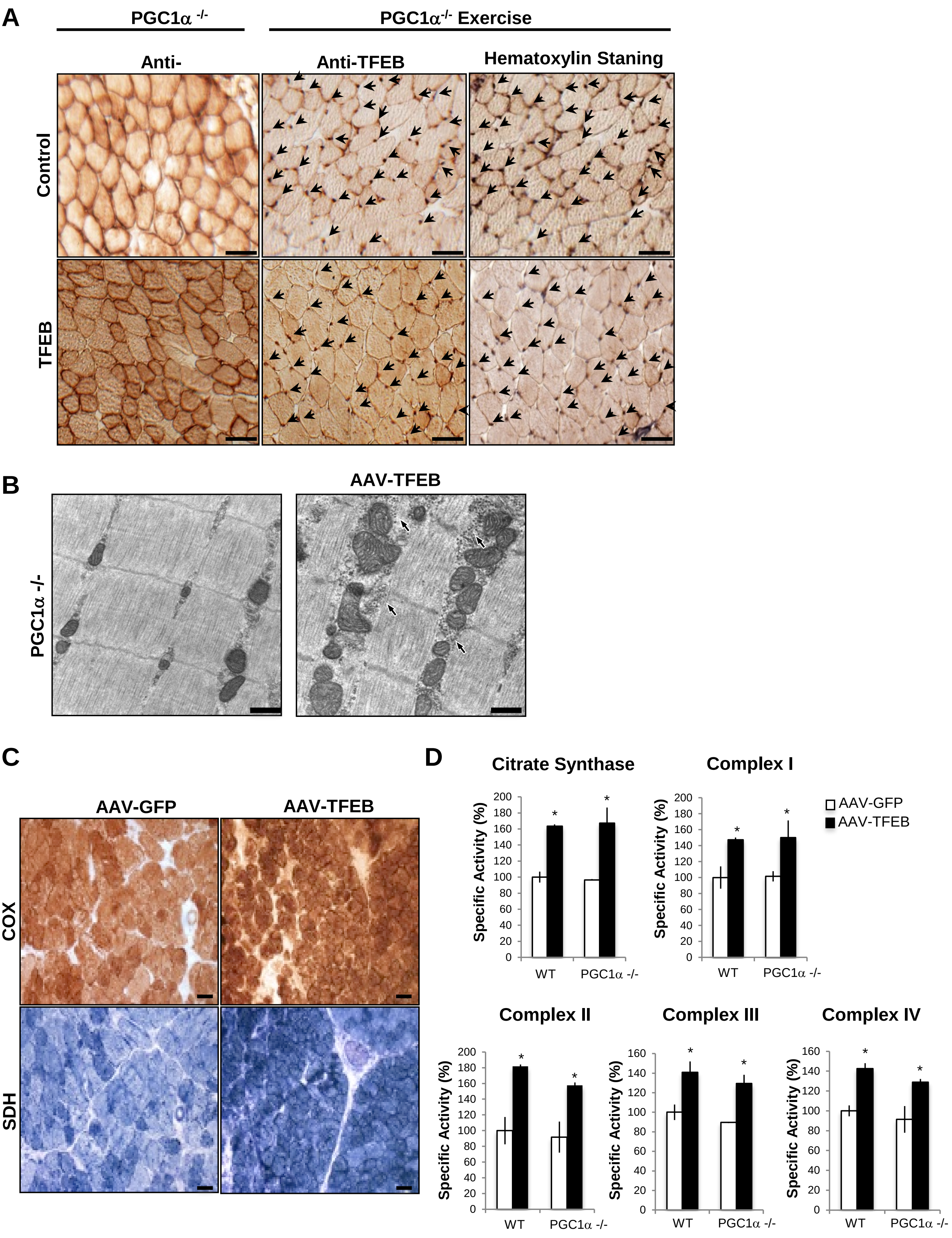
A
PGC1α -/-
PGC1α-/- Exercise
Anti-
Anti-TFEB
Hematoxylin Staning
Control
TFEB
B
AAV-TFEB
PGC1α -/-
C
AAV-GFP
AAV-TFEB
COX
SDH
D
Citrate Synthase
Complex I
AAV-GFP
AAV-TFEB
Specific Activity (%)
WT
PGC1α -/-
Complex II
Complex III
Complex IV
*

Figure S4. Exercise induces TFEB expression and nuclear localization in PGC1α-/- muscle. Related to Figure 3. (A) TFEB immunohistochemical analysis of PGC1α-/- muscles from sedentary and exercised mice. The muscles were infected by intramuscular injection of AAV2.1 control and AAV2.1-*TFEB* virus. Gastrocnemius muscle cryosections were immunostained by using anti-TFEB antibody and counter- stained with hematotoxylin for nuclei detection. Control means endogenous TFEB in muscles infected with control virus. TFEB means the transgene TFEB in muscles infected with AAV-*TFEB*. Arrows indicate exercise-induced TFEB nuclear localization. The scale bars represent 100 nm. (B-D) PGC1α is not required for mitochondrial biogenesis induced by TFEB. (B) EM of PGC1α-/- muscles that were infected with AAV2.1-*TFEB* or AAV2.1-*GFP*. The electron micrographs reveal an accumulation of mitochondria in *TFEB*-overexpressing muscle and a granular staining consistent with glycogen b- particles. The scale bars represent 500 nm. (C) COX and SDH staining of cryostatic sections from PGC1α-/- muscles transfected by AAV2.1-*GFP* or AAV2.1-*TFEB*. The scale bars represent 100 nm. (D) The mitochondrial respiratory chain activity in control and *TFEB*-overexpressing muscles. Data are expressed as percentage respect to the WT muscles infected with AAV-*GFP* control vector. Error bars represent mean ± SE for n=3; *p<0.05.

Figure S5.

A

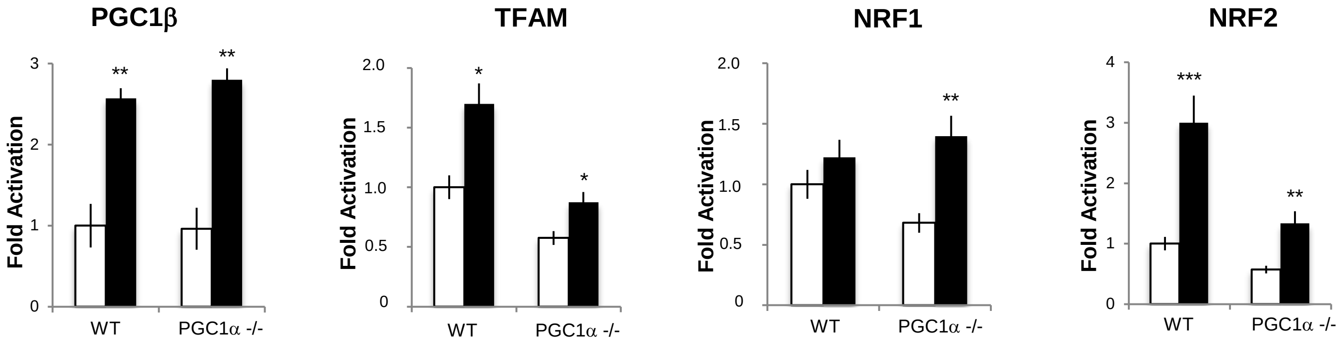
PGC1β
TFAM
NRF1
NRF2
Fold Activation
WT
PGC1α -/-
**
*
***

B

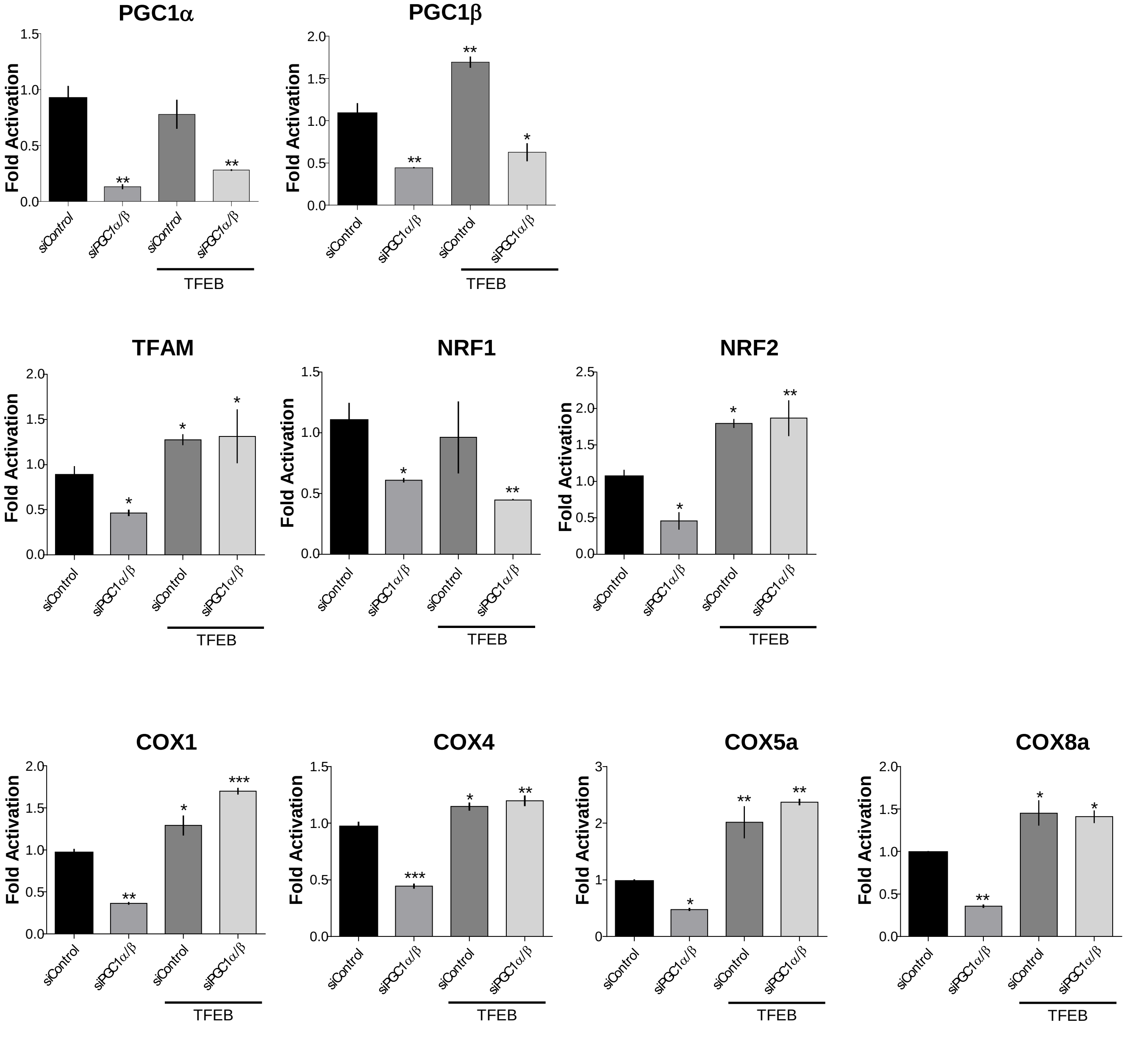
PGC1α
PGC1β
TFAM
NRF1
NRF2
COX1
COX4
COX5a
COX8a
Fold Activation
siControl
siPGC1α/β
TFEB

Figure S5. TFEB controls mitochondrial biogenesis and activity by a PGC1α and PGC1β- independent mechanism. Related to Figure 3. (A) Expression analysis of genes related to mitochondrial biogenesis in WT and PGC1α-/- muscles that were transfected by AAV2.1-*GFP* (white bar) or AAV2.1-*TFEB* (black bar). Error bars represent mean ± SE for n=3; *p<0.05, **p<0.01, ***p<0.001. (B) Expression analysis of genes related to mitochondrial biogenesis and mitochondrial respirator chain in Hela cells silenced for PGC1α PGC1β and transfected with *TFEB* or empty vector. Error bars represent mean ± SE for n=3; *p<0.05, **p<0.01, ***p<and 0.001.

Figure S6.

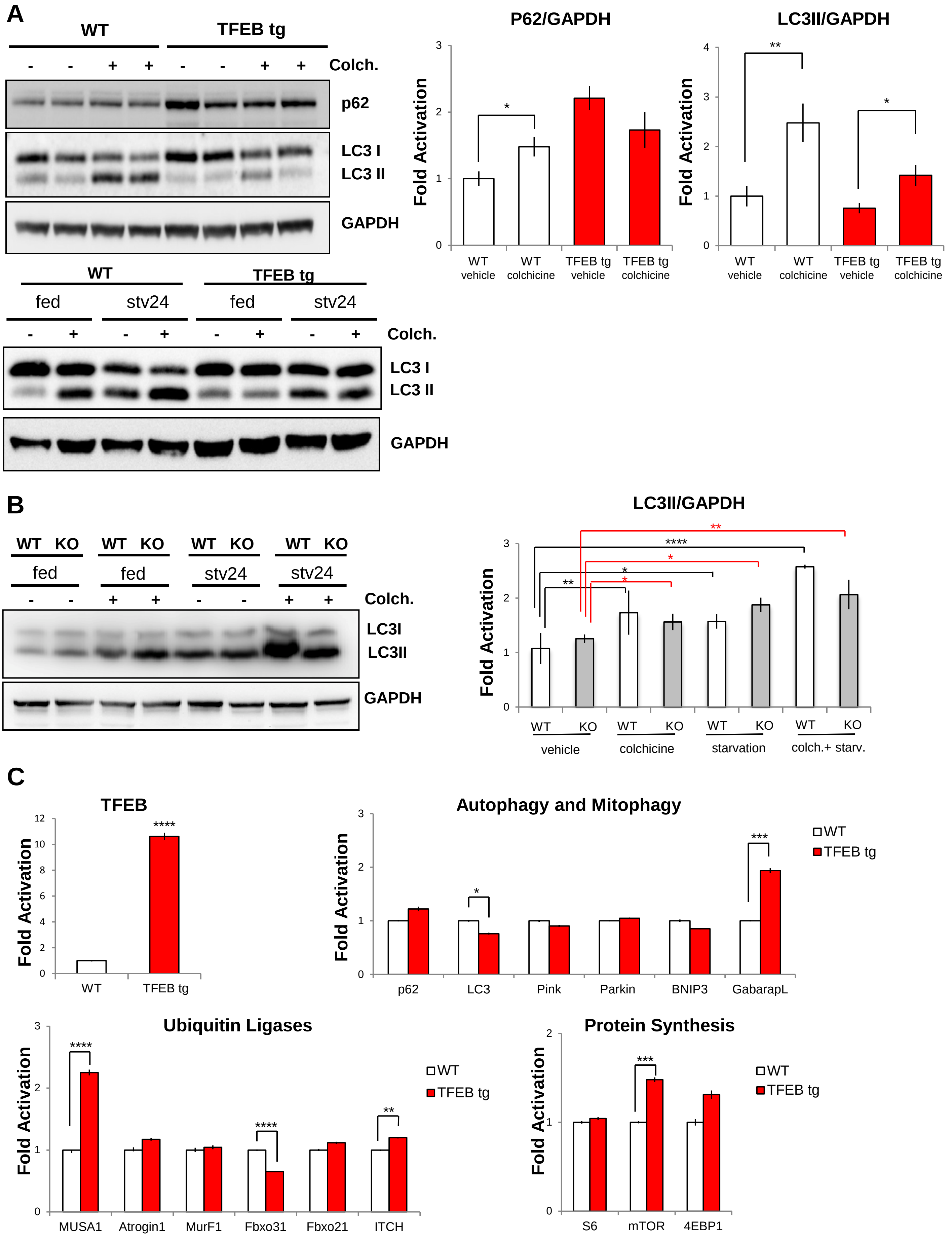
A
WT
TFEB tg
- - + + - - + + Colch.
p62
LC3 I
LC3 II
GAPDH
P62/GAPDH
Fold Activation
3
2
1
0
*
WT vehicle
WT colchicine
TFEB tg vehicle
TFEB tg colchicine
LC3II/GAPDH
4
**
*
WT
TFEB tg
fed
stv24
fed
stv24
- + - + - + - + Colch.
LC3 I
LC3 II
GAPDH
B
WT KO
fed
stv24
- - + + - - + + Colch.
LC3I
LC3II
GAPDH
LC3II/GAPDH
****
*
**
WT KO
vehicle
colchicine
starvation
colch.+ starv.
C
TFEB
12
10
8
6
4
2
****
WT
TFEB tg
Autophagy and Mitophagy
***
p62
LC3
Pink
Parkin
BNIP3
GabarapL
Ubiquitin Ligases
MUSA1
Atrogin1
MurF1
Fbxo31
Fbxo21
ITCH
Protein Synthesis
S6
mTOR
4EBP1

Figure S6. Autophagy flux is not changed by TFEB-overexpression or deletion. **Related to Figure 5.** (A) Upper panel: representative blots of p62 and LC3 from protein lysates of gastrocnemius muscles of WT and TFEB transgenic (tg) animals. Mice were treated with colchicine (Colch.) or vehicle for autophagy flux measurements. The densitometric quantification is depicted on the right panel. Lower panel: immunoblot analysis of LC3 from protein lysates of gastrocnemius muscles from fed or 24 h fasted TFEB tg or control mice. GAPDH was used as loading control. (B) Representative blot and densitometric quantification of LC3 from protein lysates from gastrocnemius muscles of fed and starved *TFEB-KO* or controls mice. Mice were treated with colchicine or vehicle for autophagy flux measurements. GAPDH was used as loading control. Data are representative of four independent experiments. (C) Quantitative RT-PCR of TFEB, autophagy/mitophagy (*p62/SQSTM1*, *LC3*, *Pink*, *Parkin*, *Bnip3* and *Gabarapl*), atrophy-related ubiquitin ligases (*MusA1*, *Atrogin1*, *MuRF1*, *Fbxo31*, *Fbxo21 and Itch*) and protein synthesis (S6, mTOR and *4EBP1*) transcripts. Data are normalized to GAPDH and expressed as fold increase of control animals. *n*=4 muscles in each group Values are mean ± SE *p<0.05, ***p<0.001, ****p<0.0001.

Figure S7.

A

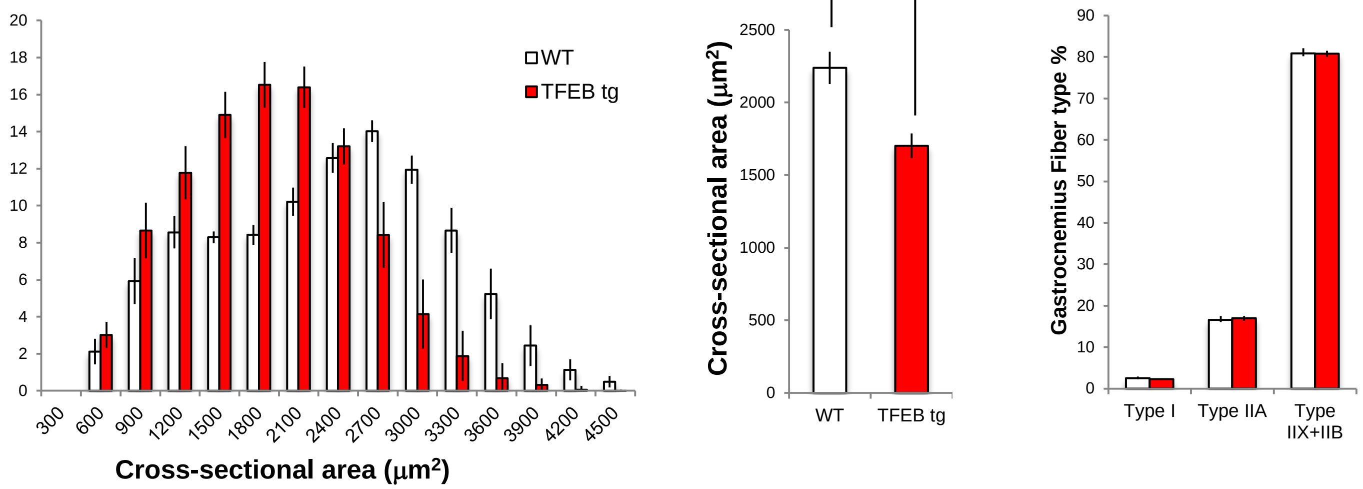

WT
TFEB tg
Cross-sectional area (μm²)
*
Cross-sectional area (μm²)
WT
TFEB tg
Gastrocnemius Fiber type %
Type I
Type IIA
Type IIX+IIB

B

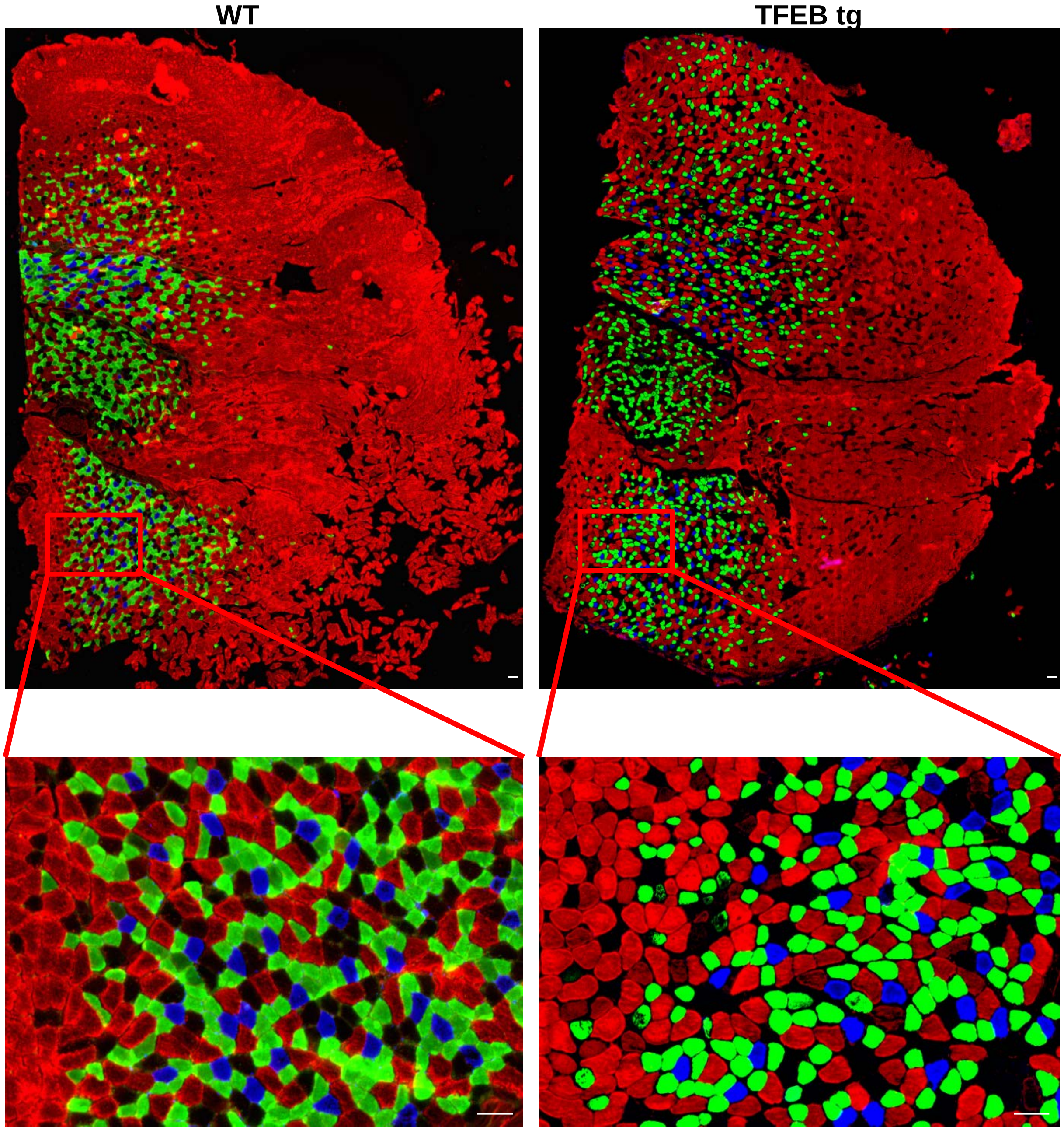

WT
TFEB tg

Figure S7. TFEB-overexpression mouse muscles modifies fiber size but does not affect MHC expression. Related to Figure 5. (A) Frequency histograms showing the distribution of myofibers cross-sectional areas (μm2) from gastrocnemius muscles of inducible muscle-specific TFEB transgenic (black bars) and control male mice (white bars). (B) Average cross-sectional area of myofibers from grastrocnemius muscles of TFEB tg and control male mice. Values are shown as means ± SE *p<0.05, from 4 muscles in each group. Percentage of type I and type II fibers in WT and TFEB tg was calculated using ImageJ 1.47 software (see Materials and Methods for details). Values are shown as means ± SE *p<0.05, from 3 muscles in each group. (c) Cryosections of Gastrocnemius muscle were immunostained for the different MHCI, MHCIIa, and MHCIIb myosins. Type I (blue), type IIA (green), type IIB (red) and type IIX (unstained) fibers are depicted. The scale bars represent 100 nm.

Supplemental Tables

Table S1. TFEB overexpression muscle dataset. Related to Figure 1. Complete list of differentially expressed genes in TFEB overexpression muscle microarray dataset (GSE62975). The genes significantly (FDR<0.05; FoldChange ≥1.5) up-regulated and down-regulated are highlighted in red and in green, respectively.
(supplied as Excel file: Supplemental Table 1.xlsx).

Table S2. TFEB-KO muscle dataset. Related to Figure 1. Complete list of differentially expressed genes in TFEB-KO muscle microarray dataset (GSE62976). The genes significantly (FDR<0.05) up-regulated and down-regulated are highlighted in red and in green, respectively.
(supplied as Excel file: Supplemental Table 2.xlsx).

Table S3. TFEB controls lipid and glucose metabolism in muscle. Related to Figure 1. List of Lipid and Glucose Metabolism related terms related to the GOEA induced by TFEB overexpression and inhibited by TFEB-KO. For each term the genes significantly induced by TFEB overexpression (Supplemental Table BPALL_UP_OverTFEB) and inhibited by TFEB-KO (Supplemental Table BPALL_DOWN_KO-TFEB) are reported in table.
(supplied as Excel file: Supplemental Table 3.xlsx).

Tables S4. Mitochondrial genes up-regulated in TFEB overexpression muscle. Related to Figure 1. List of 38 genes significantly up-regulated in TFEB overexpression muscle dataset, with a main localization in the mitochondrion.

**Gene Symbol**	**Gene Title**
Abat	4-aminobutyrate aminotransferase
Arl2bp	ADP-ribosylation factor-like 2 binding protein
Bak1	BCL2-antagonist/killer 1
Bik	BCL2-interacting killer
Bid	BH3 interacting domain death agonist
Cds2	CDP-diacylglycerol synthase (phosphatidate cytidylyltransferase) 2
Dna2	DNA replication helicase 2 homolog (yeast)
Htra2	HtrA serine peptidase 2
Rab11fip5	RAB11 family interacting protein 5 (class I)
Acsl6	acyl-CoA synthetase long-chain family member 6
Aldh18a1	aldehyde dehydrogenase 18 family, member A1
Alas1	aminolevulinic acid synthase 1
Cpt1a	carnitine palmitoyltransferase 1a, liver
Cidea	cell death-inducing DNA fragmentation factor, alpha subunit-like effector A
Ckmt1	creatine kinase, mitochondrial 1, ubiquitous
Cyb5r3	cytochrome b5 reductase 3
Cox6a1	cytochrome c oxidase, subunit VI a, polypeptide 1; predicted gene 7795
Gyk	glycerol kinase
Gatm	glycine amidinotransferase (L-arginine:glycine amidinotransferase)
Hspa1b	heat shock protein 1B; heat shock protein 1A; heat shock protein 1-like
Hk1	hexokinase 1
Me2	malic enzyme 2, NAD(+)-dependent, mitochondrial
Msto1	misato homolog 1 (Drosophila)
Mrpl39	mitochondrial ribosomal protein L39
Maoa	monoamine oxidase A
Nos1	nitric oxide synthase 1, neuronal
Ppif	peptidylprolyl isomerase F (cyclophilin F)

Pmaip1	phorbol-12-myristate-13-acetate-induced protein 1
Sfxn1	sideroflexin 1
Ugt1a9, Ugt1a2, Ugt1a6a, Ugt1a7c, Ugt1a10, Ugt1a1, Ugt1a5, Ugt1a6b	UDP glucuronosyltransferase 1 family
Cox7c	similar to cytochrome c oxidase, subunit VIIc; predicted gene 3386
Slc25a5	solute carrier family 25 (mitochondrial carrier, adenine nucleotide translocator), member 5; similar to ADP/ATP translocase 2 (Adenine nucleotide translocator 2) (ANT 2) (ADP,ATP carrier protein 2)
Slc25a24	solute carrier family 25 (mitochondrial carrier, phosphate carrier), member 24
Slc25a36	solute carrier family 25, member 36
Slc25a37	solute carrier family 25, member 37
Slc25a45	solute carrier family 25, member 45
Tmlhe	trimethyllysine hydroxylase, epsilon
Tdrd7	tudor domain containing 7

Table S5. Mitochondrial genes down-regulated in TFEB-KO muscle. Related to Figure 1. List of 73 genes significantly inhibited in the TFEB-KO muscle dataset, with a main localization in the mitochondrion.

**Gene Symbol**	**Gene Title**
Abcb8	ATP-binding cassette, sub-family B (MDR/TAP), member 8
Aco2	aconitase 2, mitochondrial
Acox1	acyl-Coenzyme A oxidase 1, palmitoyl
Acsl1	acyl-CoA synthetase long-chain family member 1
Adhfe1	alcohol dehydrogenase, iron containing, 1
Adsl	adenylosuccinate lyase
Ak3	adenylate kinase 3
Aldh2	aldehyde dehydrogenase 2, mitochondrial
Araf	v-raf murine sarcoma 3611 viral oncogene homolog
Atp5a1	ATP synthase, H+ transporting, mitochondrial F1 complex, alpha subunit, isoform 1
Bckdhb	branched chain ketoacid dehydrogenase E1, beta polypeptide; similar to 3-methyl-2-oxobutanoate dehydrogenase
Bdh1	3-hydroxybutyrate dehydrogenase, type 1
Ccdc123	similar to RIKEN cDNA 2610507L03 gene; coiled-coil domain containing 123
Coq5	coenzyme Q5 homolog, methyltransferase (yeast)
Cox15	COX15 homolog, cytochrome c oxidase assembly protein (yeast)
Cox8b	cytochrome c oxidase, subunit VIIIb
Cpt1b	carnitine palmitoyltransferase 1b, muscle
Cs	citrate synthase
Dhrs4	dehydrogenase/reductase (SDR family) member 4
Dhx30	DEAH (Asp-Glu-Ala-His) box polypeptide 30
Dnaja3	DnaJ (Hsp40) homolog, subfamily A, member 3
Dnajc11	DnaJ (Hsp40) homolog, subfamily C, member 11
Echs1	enoyl Coenzyme A hydratase, short chain, 1, mitochondrial
Ecsit	ECSIT homolog (Drosophila)

Fth1	ferritin heavy chain 1
Gatm	glycine amidinotransferase (L-arginine:glycine amidinotransferase)
Gcdh	glutaryl-Coenzyme A dehydrogenase
Gfm2	G elongation factor, mitochondrial 2
Got2	glutamate oxaloacetate transaminase 2, mitochondrial
Gpam	glycerol-3-phosphate acyltransferase, mitochondrial
Gpd1	glycerol-3-phosphate dehydrogenase 1 (soluble)
Gpd2	glycerol phosphate dehydrogenase 2, mitochondrial
Ide	insulin degrading enzyme
Idh2	isocitrate dehydrogenase 2 (NADP+), mitochondrial
Idh3a	isocitrate dehydrogenase 3 (NAD+) alpha
Immt	inner membrane protein, mitochondrial
Isca1	iron-sulfur cluster assembly 1 homolog (S. cerevisiae)
Ivd	isovaleryl coenzyme A dehydrogenase
Ldhb	lactate dehydrogenase B; predicted gene 5514
Mavs	mitochondrial antiviral signaling protein
Mccc2	methylcrotonoyl-Coenzyme A carboxylase 2 (beta)
Mdh2	malate dehydrogenase 2, NAD (mitochondrial)
Mfn1	mitofusin 1
Mrpl15	mitochondrial ribosomal protein L15
Myl10	myosin, light chain 10, regulatory
Ndufa10	NADH dehydrogenase (ubiquinone) 1 alpha
Ndufv1	NADH dehydrogenase (ubiquinone) flavoprotein 1
LOC100045796, Nfs1	nitrogen fixation gene 1 (S. cerevisiae)
Nos1	nitric oxide synthase 1, neuronal
Nudt19	nudix (nucleoside diphosphate linked moiety X)-
Oxct1	3-oxoacid CoA transferase 1
Parl	presenilin associated, rhomboid-like
Pdk2	pyruvate dehydrogenase kinase, isoenzyme 2
Pln	phospholamban
Pnkd	paroxysmal nonkinesiogenic dyskinesia
Ppif	peptidylprolyl isomerase F (cyclophilin F)
Prdx6	Peroxiredoxin-6 (Antioxidant protein 2)
Pxmp2	peroxisomal membrane protein 2
Rfk	riboflavin kinase
Rhot2	ras homolog gene family, member T2
Rtn4ip1	reticulon 4 interacting protein 1
Sdhb	succinate dehydrogenase complex, subunit B, iron
Arts (Sept4)	septin 4
Shmt1	serine hydroxymethyltransferase 1 (soluble)
Slc25a15	solute carrier family 25 (mitochondrial carrier
Suclg2	succinate-Coenzyme A ligase, GDP-forming, beta
Supv3l1	suppressor of var1, 3-like 1 (S. cerevisiae)
Tmem186	transmembrane protein 186
Tufm	predicted gene 9755; Tu translation elongation
Ung	uracil DNA glycosylase
Uqcrc1	ubiquinol-cytochrome c reductase core protein 1

Table S6. CLEAR sites on mitochondrial genes. Related to Figure 2. Position and sequence of the CLEAR sites (i.e. the consensus TFEB binding-site) in the promoter region (1Kb upstream the TSS in the human and Mouse promoters) of mitochondrial genes down-regulated in TFEB-KO muscle.
(supplied as Excel file: Supplemental Table 6.xlsx).

Table S7. CLEAR sites in promoter region of genes related to mitochondria biogenesis and glucose metabolism. Related to Figure 2 and Figure 7. Position and sequence of TFEB binding site on the GLUT1, GLUT4, nNOS, TFAM, NRF1 and NRF2 promoter.
(supplied as Excel file: Supplemental Table 7.xlsx).

Supplemental Experimental Procedures

Cell culture, Plasmids and Transfection Reagent.
C2C12 and HeLa cells were purchased from ATCC and cultured in DMEM media supplemented with 10% fetal bovine serum, 200 μM L-Glutamine, 100 μM sodium pyruvate, in 5% CO_2 at 37° C. Cells were transfected with pDsRed2-Mito (Clontech) and Human full-lenght *TFEB*-GFP or *TFEB*3XFlag, both constructs were previously described (Martina et al., 2012; Sardiello et al., 2009). Cells were transfected using TransIT®-LT1 Transfection Reagent (MirusBio) according to the protocol from the manufacturers. Cells were silenced with PGC1α siRNA oligonucleotides (Thermo Dhamacon, L-005111-00), PGC1β siRNA oligonucleotides (Thermo Dhamacon, L-008556-00) or non-targeting siRNAs (Thermo Dhamacon, D-001810-10-05), by direct or reverse transfection, using Lipofectamine RNAiMAX reagent (Invitrogen) according to the protocol from the manufacturer. siRNA-transfected cells were collected after 48 h, if not otherwise stated.

Real-time PCR.
For mtDNA content analysis, SYBR Green real-time PCR was performed using primers specific to a mouse mtDNA region in the ND1 gene and primers specific to RNaseP, a single copy gene taken as a nuclear gene reference, as described (Viscomi et al., 2009). For the analysis of transcripts, total RNA was extracted from liquid nitrogen snap frozen muscle by Trizol, according to the manufacturer's instructions (Invitrogen, Carlsbad, CA, USA). Of total RNA, 2 μg was treated with RNase free-DNase and retrotranscribed using the ''cDNA cycle'' kit (QuantiTect Reverse Transcription Kit - QIAGEN). Approximately 2–5 ng of cDNA was used for real-time PCR assay (SYBR®Green PCR Master Mix Applied Biosystem) using primers specific for amplification of several genes. Autophagic, Lipid Metabolism and Glucose Metabolism gene-specific primers are listed below. Fold change values were calculated using the ΔΔCt method. An unpaired *t*-test was used to calculate statistical significance.

List of murine primers for RT-PCR

**Gene Name**	**Primer Sequence**
TCFEB F	GCAGAAGAAAGACAATCACAA
TCFEB R	GCCTTGGGGATCAGCATT
GLUT1 F	AGCAGCAAGAAGGTGACG
GLUT1 R	CACGGAGAGAGACCAAAGC
GLUT4 F	CCGCGGCCTCCTATGAGATACT
GLUT4 R	AGGCACCCCGAAGATGAGT
NOS1 F	CCAAAGCAGAGATGAAAGACACA
NOS1 R	TCTTGGTAGGAGACTGTTTGC
PGC1alpha F	AGCCGTGACCAGTGACAACGAG
PGC1alpha R	GCTGCATGGTTCTGAGTGCTAAG
GAPDH F	TGCACCACCAACTGCTTAGC
GAPDH R	TCTTCTGGGTGGCAGTGATC
TBC1D1 F	GTCCCGGGGTAATAAAGCCA
TBC1D1 R	TTGTCACCCATGGACAGCTC
PGC1-beta F	ACTGAAAGAGGCCCAGCAGA
PGC1-beta R	ATTGGAAGGGCCTTGTCTGA
PPAR-alpha F	CCTACGCTTGGGGATGAAGA
PPAR-alpha R	CCCCATTTCGGTAGCAGGTA
DLAT F	TGTTTCATCGGTGTTGAGTCTG
DLAT R	TGCTAATGATGTGCCCTTGTG
CRAT F	GGAGAAGAGAGCCAGTCCAG
CRAT R	AGATAATCCTCCCACCGCTG

CPT1b F	GACAGTACCCTCCTTCCACC
CPT1b R	ACCAGCAAGAACAGGGTAAGA
CS F	GACCCTCGCTATTCCTGTCA
CS R	AGTTCATCTCCGTCATGCCA
COX8B F	CGAAGTTCACAGTGGTTCCC
COX8B R	GCTGGAACCATGAAGCCAAC
MDH2 F	TGAACGGGAAGGAAGGAGTC
MDH2 R	AATGCCCAGGTTCTTCTCCA
CYC1 F	GGCTCCTCCCATCTACACAG
CYC1 R	CTGACCACTTATGCCGCTTC
ATP5A1 F	GACAGTACCCTCCTTCCACC
ATP5A1 R	ACCAGCAAGAACAGGGTAAGA
SDHA F	TTACCTGCGTTTCCCCTCAT
SDHA R	AAGTCTGGCGCAACTCAATC
NRF1 F	CAGACACGTTTGCTTCGGAAA
NRF1 R	CCCACTCGCGTCGTGTACT
NRF2 F	GATCAGGCGACATGTTAACGTT
NRF2 R	AGAGCCCAGTCAAACCCTTTC
HK2 F	TGGGTTTCACCTTCTCGTTC
HK2 R	TGGACTTGAACCCCTTAGTCC
HK1 F	TGGTGGAAAAGATCCGAGAG
HK1 R	TTTGGTGCATGATTCTGGAG
COX1 F	TGCTAGCCGCAGGCATTACT
COX1 R	CGGGATCAAAGAAAGTTGTGTTT
COX2 F	CAGGCCGACTAAATCAAGCAA
COX2 R	GAGCATTGGCCATAGAATAATCCT
COX4 F	TCACTGCGCTCGTTCTGAT
COX4 R	CGATCGAAAGTATGAGGGATG
COX5a F	TCATCCAGGAACTTAGACCAACT
COX5a R	AGTCCTTAGGAAGCCCATCG
TFAM F	AGATATGGGTGTGGCCCTTG
TFAM R	AAAGCCTGGCAGCTTCTTTG
CD36 F	CCAAATGAAGATGAGCATAGGACAT
CD36 R	GTTGACCTGCAGTCGTTTTGC
ND1 F	CATAAGCCTGCGTCAGATCA
ND1 R	CCTGTGTTGGGTTGACAGTG
RNasiP F	GCCTACACTGGAGTCCGTGCTACT
RNasiP R	CTGACCACACACGAGCTGGTAGAA
PPAR gamma F	CGTGCAGCTACTGCATGTGA
PPAR gamma R	GGGTGGGACTTTCCTGCTAA
PPAR delta F	ATGGGGGACCAGAACACAC
PPAR delta R	GGAGGAATTCTGGGAGAGGT
GYS F	TTGGAAGACTGGGAGGATGA
GYS R	CATTCATCCCCTGTCACCTT
PYGM F	CTACGAAAAGGACCCCAG
PYGM R	CATGGTGTTCTGCAGTGTC
Atrogin F	GCAAACACTGCCACATTCTCTC
Atrogin R	CTTGAGGGGAAAGTGAGACG
Murf1 F	ACCTGCTGGTGGAAAACATC
Murf1 R	ACCTGCTGGTGGAAAACATC

Fbxo31 F	GTATGGCGTTTGTGAGAACC
Fbxo31 R	AGCCCCAAAATGTGTCTGTA
Fbxo21 F	TCAATAACCTCAAGGCGTTC
Fbxo21 R	GTTTTGCACACAAGCTCCA
ITCH F	CCACCCACCCCACGAAGACC
ITCH R	CTAGGGCCCGAGCCTCCAGA
p62 F	CCCAGTGTCTTGGCATTCTT
p62 R	AGGGAAAGCAGAGGAAGCTC
LC3 F	CACTGCTCTGTCTTGTGTAGGTTG
LC3 R	TCGTTGTGCCTTTATTAGTGCATC
PINK F	TGGAATATCTCGGCAGGTTC
PINK R	CGGATGATGTTAGGGTGTGG
Parkin F	CCATCTTGCTGGGACGA
Parkin R	GCCTGTTGCTGGTGATCA
BNIP3 F	TTCCACTAGCACCTTCTGATGA
BNIP3 R	GAACACCGCATTTACAGAACAA
GabarapL F	CATCGTGGAGAAGGCTCCTA
GabarapL R	ATACAGCTGGCCCATGGTAG
mTOR F	GAGAAGGGTATGAATCGAGATGA
mTOR R	CCCATGAGGTCTTTGCAGTA
S6 F	AGCCCAGGACCAAAGCACCCAA
S6 R	TCCTGGCGCTTTTCTTTGGCTTCC
MUSA1 F	TCGTGGAATGGTAATCTTGC
MUSA1 R	CCTCCCGTTTCTCTATCACG

List of human primers for RT-PCR

PGC1 alpha F	TAGTCCTTCCTCCATGCCTG
PGC1 alpha R	TGCATGGTTCTGGGTACTGA
PGC1 beta F	CTCTTCACCCTGCCACTCC
PGC1 beta R	ACCTCGCACTCCTCAATCTC
TFAM F	TTAGAAGAATTGCCCAGCGT
TFAM R	TTCTTTATATACCTGCCACTCCG
NRF1 F	CTTGTACCATCACAGACTGTAGT
NRF1 R	ACGGCAGAATAATTCACTTGGG
NRF2 F	GCCCCTGTTGATTTAGACGG
NRF2 R	GTTTGGCTTCTGGACTTGGA
COX1 F	ACCCTAGACCAAACCTACGC
COX1 R	TGTATGCATCGGGGTAGTCC
COX4 F	CAAGCGAGCAATTTCCACCT
COX4 R	GTCACGCCGATCCATATAAGC
COX5a F	TGCCTGGGAATTGCGTAAAG
COX5a R	ATTTAACCGTCTGCATGCCC
COX8a F	GAGGGGAAGCTTGGGATCAT
COX8a R	CCTGTAGGTCTCCAGGTGTG
ND1 F	CCTAAAACCCGCCACATCTA
ND1 R	GCCTAGGTTGAGGTTGACCA

Generation of muscle-specific TFEB Knockout and inducible muscle-specific transgenic mice and in vivo experimental procedures.

Mice bearing *Tcfeb*-floxed alleles (Tcfeb$^{f/f}$) (Settembre et al., 2012) were crossed with transgenic mice expressing Cre either under the control of a Myosin Light Chain 1 fast promoter (*MLC1f-Cre*) (Bothe et al., 2000). Genomic DNA isolated from Tcfeb$^{f/f}$ mice was subjected to PCR analysis. Cre-mediated recombination was confirmed by PCR with genomic DNA from gastrocnemius muscles using the primers Cre forward: 3′-CACCAGCCAGCTATCAACTCG-5′ and Cre reverse: 3′-TTACATTGGTCC AGCCACCAG-5′.

The Pgc1α Knockout mouse line was obtained from Jackson Laboratory (Bruce Spiegelman, Dana- Farber Cancer Institute) (Lin et al., 2004). The muscle-specific Pgc1α Knockout mouse were generated by crossing the Pgc1α floxed mice from Jackson Laboratory (Bruce Spiegelman, Dana-Farber Cancer Institute) with *MLC1f-Cre* transgenic mice.

To generate inducible muscle-specific TFEB transgenic animals, *Tcfeb*3XFlag cDNA was inserted after a CAGCAT cassette [chicken actin promoter (CAG) followed by chloramphenicol acetyltransferase (CAT) cDNA flanked by 2 loxP sites] and used to generate transgenic mice (Baylor College of Medicine transgenic core) (Settembre et al., 2011). Mice were then crossed with transgenic mice expressing Cre-ER driven by human skeletal actin promoter (HSA). Tamoxifen-induced Cre LoxP recombination was activated by IP injection of tamoxifen-containing for 3 days. Muscles were collected 2 weeks after the tamoxifen treatment. Cre-negative littermates, also receiving tamoxifen treatment, were used as controls. Adult mice (3 to 5 months-old) of the same sex and age were used for each individual experiment.

All mice used were males and maintained in a C57BL/6 strain background. For all experiments involving *TFEB*-KO mice, the control mice were mice Tcfeb$^{f/f}$ that did not carry the *MLC1f-Cre* transgene. Standard food and water were given ad libitum. Mice were maintained in a temperature - and humidity-controlled animal-care facility, with a 12 hr light/dark cycle and free access to water and food (Standard Diet, Mucedola, Italy). All experiments were performed on 3- to 5-month-old male (28–32*g*), mice of the same sex and age were used for each individual experiment. *In vivo TFEB* overexpression experiments were performed by i.m. injection of a total dose of 10^{11} GC of AAV2/1 vector preparation. Muscles were removed 21 days after injection and frozen in liquid nitrogen for subsequent analyses. For fasting experiments, control animals were fed *ad libitum*; food pellets were removed from the cages of the fasted animals. All procedures were formerly approved by the Italian Public Health, Animal Health, Nutrition and Food Safety, Italian Ministry of Health, (D.M. N°75/2014-B).

Western Blot Analysis and Antibodies.

For western blot the following antibodies were used: anti-GAPDH (cat. sc-32233) anti-nNOS (cat. sc-648) from Santa Cruz Biotechnology; anti-NDUFA9 (cat.459100), anti-SDHA (cat. 459200), anti-COX5a (cat. 459120), from Invitrogen; anti-Gys (cat. 3886), anti-pGys (cat. 3891) , anti-AMPK (cat.2532), anti-pAMPK (cat. 2531), anti-pACC (cat. 3661), anti-AKT (cat. 4691) from Cell Signaling; anti-GLUT1(cat. ab40084) from Abcam; anti-LC3B (cat. L7543), anti-p62 (cat. P0067) from Sigma Aldrich.

Biochemical Analysis of Mitochondrial Respiratory Chain Complex.

Complex I activity was measured using 50 μg mitochondrial protein in a reaction containing 250 mM sucrose, 2 mM EDTA, 100 mM Tris–HCl pH 7.4, between 10 and 100 μM decyl-ubiquinone and 2 mM KCN at 30° C. Reactions were started by the addition of 50 μM NADH and oxidation followed at 340 nm for 2 minutes. Rotenone was used to block any rotenone insensitive activity.

Complex II activity was measured by incubating 40 μg of mitochondrial protein at 30° C in a reaction mix containing 50 mM KH2PO4, pH 7.4, 20 mM succinate, 2 μg/mL antimycin A, 2 μg/mL rotenone, 2 mM KCN and 50 μM 2,6-dichlorophenolindophenol (DCPIP). Reactions were initiated by adding 50μM DB to the reaction and measuring the change in absorbance at 600 nm for 2 min.

Complex III activity is measured by incubating 20 μg mitochondrial proteins at 30° C in a reaction mix containing 250 mM sucrose, 50 mM Tris–HCl, 1 mM EDTA, 50 μM cytochrome c and 2 mM KCN. Decylubiquinol (DBH2) was used an electron donor. The reaction was started by the addition of 50 μM DBH2 and the change in absorbance of cytochrome c at 550 nm was followed for 2 min. 5 μg of antimycin A were added to duplicate reactions to measure any antimycin A-insensitive activity. Complex IV activity was measured by adding 10 μg mitochondria to a mix containing 10 mM KH2PO4, pH 7.2 and between 5 and 50 μM ferrocytochrome c. The rate of oxidation of ferrocytochrome c was recorded for 2 min (extinction coefficient of 27.2 mM− 1 cm− 1). Citrate synthase activity was measured at 30° C by using 10 μg mitochondria to a

reaction mixture containing 125 mM Tris–HCl, 100 μM DTNB (5,5′-dithiobis(2-nitrobenzoic acid) and 300 μM acetyl coenzyme A. The reaction is initiated by the addition of 500 μM oxaloacetate, and DTNB reduction at 412 nm measured for 2 min. The Mitochondrial respiratory chain activities were expressed as nmoles/min/mg of protein.

For the quantitative determination of Adenosine 5'triphosphate (ATP) the muscle gastrocnemius samples stored in liquid nitrogen were homogenized in sterile water and analyzed according to the manufacturer's instructions of ATP Bioluminescent Assay Kit (Sigma-Aldrich).

Protein Carbonyls Detection.

Carbonylation of muscle proteins was detected by using the OxyBlot Protein Oxidation Detection Kit from Millipore (Masiero et al., 2009). Spot detection and quantification was carried out using the UVP VisionWorksLS software.

Acute Exercise, Training and Fatigue Experiments

Mild exercise was performed by let mice run on the treadmill for 1 hour at 15 cm/sec speed for 4 days. Training was performed by increasing the speed of 5 cm/sec every week for a total of 7 weeks. The starting speed was the 50% of the maximal speed (exhaustion test) that corresponded to 25cm/sec and the final speed reached at the end of the training period was 55 cm/sec.

To measure muscle endurance, soleus muscles were dissected under a stereomicroscope and mounted between a force transducer (KG Scientific Instruments, Heidelberg, Germany) and a micromanipulator- controlled shaft in a small chamber in which oxygenated Krebs solution was continuously circulated and temperature maintained at 25°C. The stimulation voltage was optimized, and the length of the soleus was increased until force development during tetanus was maximal. The responses to a single stimulus (twitch) or to trains of stimuli at various rates producing unfused or fused tetani were recorded. In order to induce muscle fatigue, muscles were stimulated for 500ms at 100Hz every 2 seconds (0,25 duty cycle). Induction of fatigue ex-vivo is known to be performed at a duty cycle between 0,1 and 0,5 (Allen et al., 2008).

Mitochondrial Assays

Mitochondria from mouse skeletal muscle were isolated as described (Frezza et al., 2007). The rate of mitochondria oxygen consumption was measured at 3 0 ° C in an incubator chamber with a Clark/type O2 electrode filled with 2ml of incubation medium (125 mM KCl, 10 mM Pi.Tris, 20 mM Tris-HCl, 0,1 mM EGTA, ph 7,2). All measured were performed using mitochondria (0,2 mg mitochondrial protein/ml) incubated either with glutamate (5 mM)/ malate (2,5 mM) or /and succinate (5 mM) as substrates, in the presence (state 3) and in absence (state 4) of 100 mM ADP (Frezza et al., 2007).

Autophagic flux quantification

We monitored autophagic flux in fed and 24 h starving mice by using colchicine as previously described (Milan et al., 2015). Briefly muscle specific transgenic TFEB and *TFEB*-KO mice were treated with 0,4 mg/kg colchicine or vehicle by i.p. injection and starved. The treatment was repeated at 15 h prior to muscle harvesting.

Morphological Analysis.

For SDH activity, 8µm thick sections from frozen tissue were collected on coverslips. The reaction mix contained: 5 mM phosphate buffer pH 7.4, 5 mM EDTA, 1 mM KCN, 0.2 mM Phenazine methosulfate (PMS), 50 mM Succinic acid, 1.5 mM Nitro blue tetrazolium (NBT). NBT was the electron acceptor with PMS served as intermediate electron donor to NBT. Sections were incubated for 20 min at 37° C. For control sections, sodium malonate (10 mM) was added to the incubation medium. For Cytochrome-c Oxidase the sections were incubated 1 hr at 37° C. The reaction mix contained: 5 mM phosphate buffer pH 7.4, 0.1% 3,3'-Diaminobenzidine (DAB), 0.1% Cytochrome c, 0.02% catalase. Histochemical analyses were performed as described (Sciacco and Bonilla, 1996).

Cross-sectional area was measured using ImageJ software in at least 400 fibres and compared with the area of age-matched control. The fibre diameter was calculated as caliper width, perpendicular to the longest chord of each myofibre. The total myofibre number was calculated from entire muscle section based on assembled mosaic image (×20 magnification). (Fibre typing was determined by immunofluorescence using combinations of the following monoclonal antibodies: BA-D5 that recognizes type 1 MyHC isoform and SC-71 for type 2A MyHC isoform. Images were captured using a Leica DFC300-FX digital charge-coupled device camera by using Leica DC Viewer software, and morphometric analyses were made using the software ImageJ 1.47 version.

Immunohistochemistry

Frozen muscle serial sections (7 μm) were fixed in 4% paraformaldehyde in PBS for 10 min, washed twice in PBS (Sigma-Aldrich) for 5 min at room temperature (RT), and incubated in 0.03% H_2O_2 in Methanol for 10 min at RT. After washing in PBS, sections were incubated in blocking solution (10% normal goat serum, 5% BSA in PBS) for 1h at RT. Anti-TFEB mAb (Bethil Laboratories) was added separately to each serial section for 16 h at 4°C. Sections were washed twice in PBS, incubated with biotin-conjugated goat anti-rabbit IgG1, 5 μg/ml with 1% normal goat serum and 0,5% BSA in PBS) for 2 h at 4°C, washed twice, incubated with HRP-conjugated streptavidin (*Nova*RED substrate kit for peroxidase) (Vector Laboratories), following the manufacturer instructions, then rinsed in distilled water for 5 min, and counterstained with hematoxylin. Sections were prepared from independent muscle specimens from 3 mice for group; and 3–4 sections for each muscle were analyzed.

Electron microscopy.

Small pieces of muscle tissue were fixed in a mixture of 2% paraformaldehyde and 1% Glutaraldehyde prepared in 0.2 M Hepes. Then samples were post-fixed in a mixture of osmium tetroxide and potassium ferrocyanide, dehydrated in ethanol and propyleneoxide and embedded in epoxy resin as described previously (Polishchuk et al., 2014). Thin 65 nm sections were cut using a Leica EM UC7 ultramicrotome. EM images were acquired using a FEI Tecnai-12 electron microscope (FEI, Eindhoven, Netherlands) equipped with a VELETTA CCD digital camera (Soft Imaging Systems GmbH, Munster, Germany). Morphometric analysis of number and size of mitochondria was performed using iTEM software (Olympus SIS, Germany). Number of mitochondria was counted using the same magnification within 100 μm square field of view. For each experiment, between 154 and 196 individual mitochondrial diameters were measured from 3 mice per group.

Tissue metabolites quantification

Muscle free fatty acids were extracted as follows: briefly, pulverized muscle was homogenized in PBS, then extracted using chloroform/methanol (2:1), dried overnight and re-suspended in a solution of 60% butanol 40% Triton X-114/methanol (2:1). Measurements were normalized to protein content in the initial homogenate by DC protein assay (Bio-Rad). The quantitative determination of glycogen amount was performed according to the manufacture's instruction of the Glycogen Colorimetric/Fluorometric Assay Kit (BioVision). For the quantitative determination of keton bodies, the muscle gastrocnemius samples stored in liquid nitrogen were homogenized and analyzed according to the manufacturer's instructions of β-hydroxybutyrate colorimetric assay kit (Vinci-Biochem). β- Hydroxybutyrate concentration is determined by a coupled enzyme reaction, which results in a colorimetric (450 nm) product, proportional to the β-Hydroxybutyrate present in muscle.

Whole Body Indirect Calorimetry.

Mice were individually housed in the chamber with a 12-h light/12-h dark cycle in an ambient temperature of 22–24° C. VO_2 and VCO_2 rates were determined under Oxymax system settings as follows: air flow, 0.6 l/min; sample flow, 0.5 l/min; settling time, 6 min; and measuring time, 3 min. The system was calibrated against a standard gas mixture to measure O_2 consumed (VO_2, ml/kg/h) and CO_2 generated (VCO_2, ml/kg/h). Energy expenditure (EE), oxygen consumption ($\dot{V}o_2$) and carbon dioxide production ($\dot{V}co_2$) during running was determined as described by Shemesh (Shemesh et al., 2014). Briefly, the mice were acclimatized to a treadmill (Columbus Instruments) by running at 10 m/min for 15 min over three consecutive days. On the fourth day, groups of mice were run at 10 m/min, progressing to 15 m/min. Energy expenditure, oxygen consumption ($\dot{V}o_2$) and carbon dioxide production ($\dot{V}co_2$) were recorded during running interval, and the total distance run to exhaustion was determined.

In Vivo Assessment of Insulin Action and Glucose Metabolism.

The 2-h EU clamp was conducted with a prime continuous infusion of human insulin (4 mill units/kg/min) and a variable infusion of 25% glucose to maintain glucose at <150 mg/dl. Insulin stimulated whole body glucose metabolism was estimated using a prime continuous infusion of [3-^{3}H] glucose (10 μCi bolus, 0.1 μCi/min; PerkinElmer Life Sciences). To determine the rate of basal glucose turnover, [3-^{3}H] glucose (0.05 μCi/min) was infused for 2 h (basal period) before starting the EU clamp, and a blood sample was taken at the end of this basal period. To assess insulin-stimulated tissue-specific glucose uptake, 2-deoxy-d-[1-^{14}C] glucose (PerkinElmer Life

Sciences) was administered as a bolus (10 μCi) 75 min after the start of the clamp. Blood samples were taken at 80, 90, 100, 110, and 120 min after the start of the EU clamp. At the end of the EU clamp, different muscle groups, adipose tissue, and liver were rapidly dissected and frozen at -80° C for analysis. For the determination of plasma [3-^{3}H]glucose and 2-deoxy-d-[1-^{14}C]glucose concentrations, plasma was deproteinized with $ZnSO_4$ and $Ba(OH)_2$, dried to remove $^{3}H_2O$, resuspended in water, and counted in scintillation fluid (Ultima Gold; Packard Instrument Co.). For the determination of tissue 2-deoxy-d-[1-^{14}C]glucose (2-DG)-6-phosphate (2-DG-6-P) content, tissue samples were homogenized, and the supernatants were subjected to an ion-exchange column to separate 2-DG-6-P from 2-DG, as described previously. The radioactivity of ^{3}H in tissue glycogen was determined by digesting tissue samples in KOH and precipitating glycogen with ethanol as previously described. Muscle glycogen synthesis was calculated as muscle [^{3}H] glycogen content divided by the area under the plasma [^{3}H] glucose-specific activity profile.

Microarray hybridization.
Total RNA (3 μg) was reverse transcribed to single-stranded cDNA with a special oligo (dT) 24 primer containing a T7 RNA promoter site, added 3' to the poly-T tract, prior to second strand synthesis (One Cycle cDNA Synthesis Kit by Affymetrix, Fremont, CA, USA). Biotinylated cRNAs were then generated, using the GeneChip IVT Labeling Kit (Affymetrix). Twenty micrograms of biotinylated cRNA was fragmented and 10 μg hybridized to the Affymetrix GeneChip Mouse 430A-2 microarrays for 16 hours at 45° C using an Affymetrix GeneChip Fluidics Station 450 according to the manufacturer's standard protocols.
For determine the changes in muscle transcriptome induced by *TFEB* overexpression, total RNA was extracted from muscle from three mice injected with an adenoviral vector that expresses mouse *Tcfeb* (AAV2.1-*TFEB*) and muscle from three mice injected with AAV2.1-*GFP* used as control.
For the analysis of *Tcfeb* knock-out (KO) mice muscle-specific total RNA was extracted from the muscle of three *TFEB*-KO mice 3 month old as compared to three WT mice used as control.

Microarray data processing.
The data discussed in this publication have been deposited in NCBIs Gene Expression Omnibus (GEO)(Edgar et al., 2002) and are accessible through GEO Series accession number GSE62975 (*TFEB*_overexpression muscle dataset) and GSE62976 (*TFEB*-KO_muscle dataset). The two muscle studies (GSE62975 and GSE62976) are part of the SuperSeries GSE62980.
The SuperSeries has been named: Expression data from mice after knockout or overexpression of *Tcfeb* in muscle. Low-level analysis to convert probe level data to gene level expression was performed using Robust Multiarray Average (RMA) implemented using the RMA function of the Bioconductor project (Gentleman et al., 2004a, b).

Statistical analysis of differential gene expression.
For each gene, a Bayesian t-test (Cyber-t) (Baldi and Long, 2001) was used on RMA normalized data to determine if there was a significant difference in expression between mice overexpressing *TFEB* (*TFEB* overexpression muscle mice) versus not-injected mice used as control and *Tcfeb* knock-down (KO) mice muscle-specific versus WT mice. P-value adjustment for multiple comparisons was done with the False Discovery Rate (FDR) of Benjamini-Hochberg (Klipper-Aurbach et al., 1995). The threshold for statistical significance chosen for both muscle microarray datasets analysis was FDR<0.05; a further filtering was performed for *TFEB* overexpression muscle dataset (GSE62975) by selection genes with an absolute Fold Change ≥1.5 for both increased (up-regulated genes) and decreased (down-regulated genes) expression levels.

Microarray data analysis.
Gene Ontology Enrichment Analysis (GOEA) (Dennis et al., 2003) was performed for each microarray dataset on the up-regulated and down-regulated gene lists, separately, by using the DAVID online tool (DAVID Bioinformatics Resources 6.7) restricting the output to all Biological Process terms (BP_ALL) and to all Cellular Compartments terms (CC_ALL). The threshold for statistical significance of GOEA was FDR≤10% and Enrichment Score ≥1.5. 73 out of 461 genes significantly inhibited in the *TFEB*-KO_muscle dataset were mainly localized in the mitochondrion (GO:0005739) as shown in the Table S5. Promoter analysis to isolate potential TFEB binding sites (i.e. CLEAR motifs) was also performed on a region of 1kb up-stream the TSS of the human and mouse promoter sequences of the 73 Mitochondrial genes; 52 out of the total 73 Mitochondrial genes contain at least one CLEAR motif in the human or mouse promoter, or in both (Table S6, 73 Mitochondrial_DW_*TFEB*-KO_CLEAR). 38 out of 1512 genes significantly induced in the *TFEB* overexpression muscle dataset were localized in the

mitochondrial part (GO:0044429; refer to Table S4). In Figure 1A the Lipid and Glucose Metabolism related terms induced by *TFEB* overexpression and inhibited by *TFEB*-KO were selected from BPALL_UP in *TFEB* overexpression and from BPALL_DOWN in *TFEB*-KO, respectively. For each term the genes significantly induced by *TFEB* overexpression and inhibited by *TFEB*-KO are reported in table Table S3 Lipid and Glucose Metabolism.

Accession codes:
Primary accessions Gene Expression Omnibus
The two muscle studies (GSE62975 and GSE62976) are part of the SuperSeries **GSE62980**. The SuperSeries has been named: Expression data from mice after knockout or overexpression of *Tcfeb* in muscle.
GSE62975: Expression data from injected mice overexpressing *Tcfeb* specifically in muscle
GSE62976: Expression data from *Tcfeb* KO mice specifically in muscle

Supplemental References

Allen, D.G., Lamb, G.D., and Westerblad, H. (2008). Skeletal muscle fatigue: cellular mechanisms. Physiological reviews *88*, 287-332.

Baldi, P., and Long, A.D. (2001). A Bayesian framework for the analysis of microarray expression data: regularized t -test and statistical inferences of gene changes. Bioinformatics *17*, 509-519.

Bothe, G.W., Haspel, J.A., Smith, C.L., Wiener, H.H., and Burden, S.J. (2000). Selective expression of Cre recombinase in skeletal muscle fibers. Genesis *26*, 165-166.

Dennis, G., Jr., Sherman, B.T., Hosack, D.A., Yang, J., Gao, W., Lane, H.C., and Lempicki, R.A. (2003). DAVID: Database for Annotation, Visualization, and Integrated Discovery. Genome Biol *4*, P3.

Edgar, R., Domrachev, M., and Lash, A.E. (2002). Gene Expression Omnibus: NCBI gene expression and hybridization array data repository. Nucleic Acids Res *30*, 207-210.

Frezza, C., Cipolat, S., and Scorrano, L. (2007). Organelle isolation: functional mitochondria from mouse liver, muscle and cultured fibroblasts. Nature protocols *2*, 287-295.

Gentleman, R.C., Carey, V.J., Bates, D.M., Bolstad, B., Dettling, M., Dudoit, S., Ellis, B., Gautier, L., Ge, Y., Gentry, J., et al. (2004a). Bioconductor: open software development for computational biology and bioinformatics. Genome Biol *5*, R80.

Gentleman, R.C., Carey, V.J., Bates, D.M., Bolstad, B., Dettling, M., Dudoit, S., Ellis, B., Gautier, L., Ge, Y., Gentry, J., et al. (2004b). Bioconductor: open software development for computational biology and bioinformatics. Genome biology *5*, R80.

Klipper-Aurbach, Y., Wasserman, M., Braunspiegel-Weintrob, N., Borstein, D., Peleg, S., Assa, S., Karp, M., Benjamini, Y., Hochberg, Y., and Laron, Z. (1995). Mathematical formulae for the prediction of the residual beta cell function during the first two years of disease in children and adolescents with insulin-dependent diabetes mellitus. Medical hypotheses *45*, 486-490.

Lin, J., Wu, P.H., Tarr, P.T., Lindenberg, K.S., St-Pierre, J., Zhang, C.Y., Mootha, V.K., Jager, S., Vianna, C.R., Reznick, R.M., et al. (2004). Defects in adaptive energy metabolism with CNS -linked hyperactivity in PGC-1alpha null mice. Cell *119*, 121-135.

Martina, J.A., Chen, Y., Gucek, M., and Puertollano, R. (2012). MTORC1 functions as a transcriptional regulator of autophagy by preventing nuclear transport of TFEB. Autophagy *8*, 903-914.

Masiero, E., Agatea, L., Mammucari, C., Blaauw, B., Loro, E., Komatsu, M., Metzger, D., Reggiani, C., Schiaffino, S., and Sandri, M. (2009). Autophagy is required to maintain muscle mass. Cell metabolism *10*, 507-515.

Milan, G., Romanello, V., Pescatore, F., Armani, A., Paik, J.H., Frasson, L., Seydel, A., Zhao, J., Abraham, R., Goldberg, A.L., et al. (2015). Regulation of autophagy and the ubiquitin-proteasome system by the FoxO transcriptional network during muscle atrophy. Nature communications *6*, 6670.

Polishchuk, E.V., Concilli, M., Iacobacci, S., Chesi, G., Pastore, N., Piccolo, P., Paladino, S., Baldantoni, D., van, I.S.C., Chan, J., et al. (2014). Wilson disease protein ATP7B utilizes lysosomal exocytosis to maintain copper homeostasis. Developmental cell *29*, 686-700.

Sardiello, M., Palmieri, M., di Ronza, A., Medina, D.L., Valenza, M., Gennarino, V.A., Di Malta, C., Donaudy, F., Embrione, V., Polishchuk, R.S., et al. (2009). A gene network regulating lysosomal biogenesis and function. Science *325*, 473-477.

Sciacco, M., and Bonilla, E. (1996). Cytochemistry and immunocytochemistry of mitochondria in tissue sections. Methods in enzymology *264*, 509-521.

Settembre, C., Di Malta, C., Polito, V.A., Garcia Arencibia, M., Vetrini, F., Erdin, S., Erdin, S.U., Huynh, T., Medina, D., Colella, P., et al. (2011). TFEB links autophagy to lysosomal biogenesis. Science *332*, 1429-1433.

Settembre, C., Zoncu, R., Medina, D.L., Vetrini, F., Erdin, S., Erdin, S., Huynh, T., Ferron, M., Karsenty, G., Vellard, M.C., et al. (2012). A lysosome-to-nucleus signalling mechanism senses and regulates the lysosome via mTOR and TFEB. The EMBO journal *31*, 1095-1108.

Shemesh, A., Wang, Y., Yang, Y., Yang, G.S., Johnson, D.E., Backer, J.M., Pessin, J.E., and Zong, H. (2014). Suppression of mTORC1 activation in acid-alpha-glucosidase-deficient cells and mice is ameliorated by leucine supplementation. American journal of physiology. Regulatory, integrative and comparative physiology *307*, R1251-1259.

Viscomi, C., Spinazzola, A., Maggioni, M., Fernandez-Vizarra, E., Massa, V., Pagano, C., Vettor, R., Mora, M., and Zeviani, M. (2009). Early-onset liver mtDNA depletion and late-onset proteinuric nephropathy in Mpv17 knockout mice. Human molecular genetics *18*, 12-26.
